# Morphometric Assessment of Occipital Condyles and Foramen Magnum Reveals Enhanced Sexual Dimorphism Detection via 3D Imaging: A Systematic Review and Meta-Analysis Utilizing Classification and Regression Trees

**DOI:** 10.3390/diagnostics15111359

**Published:** 2025-05-28

**Authors:** Christos Tsiouris, George Triantafyllou, Nektaria Karangeli, George G. Botis, Panagiotis Papadopoulos-Manolarakis, Theodosis Kalamatianos, George Tsakotos, Maria Piagkou

**Affiliations:** 1 Department of Anatomy, School of Medicine, Faculty of Health Sciences, National and Kapodistrian University of Athens, 11527 Athens, Greece; tb.christos@gmail.com (C.T.); georgerose406@gmail.com (G.T.); nekkarangeli@gmail.com (N.K.); botis_g@biomed.ntua.gr (G.G.B.); p.papado89@gmail.com (P.P.-M.); gtsakotos@gmail.com (G.T.); 2 Biomedical Engineering Laboratory, School of Electrical and Computer Engineering, National Technical University of Athens, 15773 Zografou, Greece; 3 Department of Neurosurgery, General Hospital of Nikaia-Piraeus, 18454 Athens, Greece; 4 Department of Biomedical Engineering, University of West Attica, 12243 Athens, Greece; tkalamatianos@uniwa.gr; 5 Department of Neurosurgery, Evangelismos Hospital, School of Medicine, National and Kapodistrian University of Athens, 10676 Athens, Greece

**Keywords:** foramen magnum, occipital condyles, morphometry, meta-analysis, meta-CART analysis

## Abstract

**Background:** The morphology of the occipital condyles (OCs) and foramen magnum (FM) is critical for neurosurgical planning and forensic identification. However, pooled reference values and the impact of study-level moderators on morphometric estimates remain underexplored. **Methods:** A systematic review and meta-analysis were conducted to estimate pooled morphometric values of the OCs and FM. Databases were searched for studies reporting relevant data in adult human subjects. A random-effects model was used to calculate pooled means and mean differences (MDs) by sex and side (left vs. right). Risk of bias and study quality were assessed. Subgroup analyses were conducted based on study design (osteological vs. imaging) and geographical region. Meta-CART (classification and regression trees) was used to explore moderator interactions and identify data-driven subgroups contributing to heterogeneity. **Results:** A total of 61 studies comprising 8010 adult skulls met the inclusion criteria. Substantial heterogeneity was observed across studies; most were assessed as having low-to-moderate methodological quality and a high risk of bias. The pooled mean values were as follows: OC length (OCL): 21.51 mm, OC width (OCW): 11.23 mm, OC thickness (OCT): 9.11 mm, FM length (FML): 35.02 mm, and FM width (FMW): 28.94 mm. Morphometric values reported in imaging-based studies were consistently lower than those from osteological studies. Evident sexual dimorphism was identified, with males exhibiting larger dimensions than females. The most pronounced sex-based mean differences (MDs)—approximately 2 mm—were found in OCL, FML, and FMW. In contrast, differences in OCT and OCW were under 1 mm. No significant side-related asymmetries were observed overall. Subgroup analysis revealed that sex-related MDs were more prominent in imaging studies, particularly for the right OCL and OCW. Meta-CART analysis identified study design as the strongest moderator for OCL, OCW, and FML. Sexual dimorphism was more pronounced in imaging studies but statistically insignificant in osteological samples. Furthermore, sex emerged as a stronger predictor for OCL than OCW, while geographical region had a greater impact on OCW. For OCT, geographical region was the main influencing factor, whereas sex was the primary moderator for FMW. **Conclusions:** OC and FM morphometry exhibit substantial heterogeneity across studies. Imaging-based methods more effectively detect sex-related differences, underscoring their utility in forensic identification and neurosurgical planning. These findings emphasize the need for more standardized, high-quality morphometric research to support population-specific anatomical reference data.

## 1. Introduction

**Figure 1:**
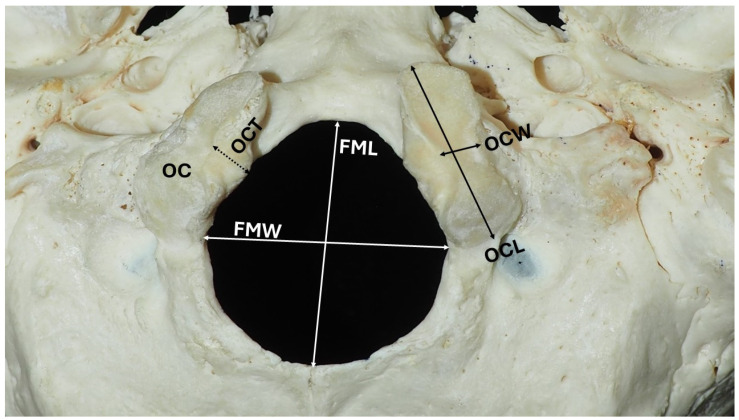
Osteological specimen depicting the measurements. OCW, OCL, OCT—occipital condyle width, length, and thickness; FMW, FML—foramen magnum width and length; and OC—occipital condyle. The specimen is part of the osteological collection of the Anatomy Department, School of Medicine, National and Kapodistrian University of Athens, and is used with the department’s permission.

The morphometry of the foramen magnum (FM) and occipital condyles (OCs) has been extensively investigated due to their anatomical, clinical, and forensic relevance. Figure 1 illustrates the key osteological landmarks and dimensions of the skull base, highlighting the FM and OCs. The occipital bone forms the posterior and basal parts of the cranium, enclosing the FM—an oval, anteromedially positioned opening flanked by the paired OCs. These oval-to-kidney-shaped condyles articulate with the atlas vertebra; their long axes converge anteromedially, with the hypoglossal canal superior to each condyle. Numerous studies have examined the length, width, and thickness of the FM and OCs, assessing their variation by sex, side, and population. Morphometric analysis of these structures plays a vital role in neurosurgery, forensic anthropology, and the assessment of craniovertebral junction abnormalities. In neurosurgical planning, accurate FM measurements are critical for procedures involving pathologies such as Chiari malformation. In forensic contexts, FM dimensions are often used for sex estimation, particularly due to the FM’s structural preservation in extreme conditions such as fires and explosions [1]. Kamath et al. [1] demonstrated that FM dimensions exhibit measurable sexual dimorphism. Using binary logistic regression and receiver operating characteristic (ROC) analysis, they reported predictive accuracies of 69.6% for sagittal diameter (SD) or anteroposterior diameter (APD) and 66.4% for transverse diameter (TD) or laterolateral diameter (LLD), supporting the utility of FM morphometry in biological profiling.

Babu et al. [2] assessed the sexing potential of FM measurements. They reported a predictive accuracy of 65.4% for the TD (also called the LLD) and 86.5% for the SD, also called the APD. When both APD and TD were combined in a binary logistic regression model, the accuracy increased to 88%. Despite this improvement, the authors cautioned that due to the considerable overlap in male and female FM measurements, the application of these dimensions for sex estimation should be limited to cases involving fragmentary remains, particularly when only the skull base is available. In such scenarios, APD and FM areas outperformed TD in distinguishing sex. Nevertheless, given the relatively high accuracy rates reported for FM dimensions, these measurements can still provide valuable supplementary evidence in a multidisciplinary forensic assessment. Ajharaj et al. [3] further explored the utility of FM and OC measurements through univariate and multivariate analyses. Individually, the FM area (66.1%), FM length (FML, 62.5%), FM width (FMW, 62.5%), and right OC length (OCL, 62.1%) demonstrated moderate accuracy in sex estimation. When all eight variables related to FM and OCs were analyzed in a multivariate model, the overall accuracy increased to 71.6%, with classification success rates of 73.3% for males and 69.9% for females. These findings underscore the potential value of combining multiple cranial base measurements for improved sex prediction accuracy.

Although numerous studies have assessed the morphometry of the FM and OCs, a comprehensive meta-analysis that integrates advanced moderator analysis remains absent. Specifically, no prior research has combined conventional meta-analytic methods with classification and regression trees (meta-CART) to investigate how multiple moderators—and their interactions—influence cranial base morphometric estimates. To address this gap, the present systematic review and meta-analysis aims to provide pooled reference values for FM and OC dimensions while exploring the influence of key moderators, including sex, side (left vs. right), geographical region, and study design (imaging-based vs. osteological). By employing traditional meta-analytic techniques and meta-CART, this study offers a novel, data-driven framework for understanding the sources of variability in FM and OC morphometry. The findings directly affect neurosurgical planning and forensic sex estimation, particularly in contexts involving incomplete cranial remains.

## 2. Materials and Methods

This meta-analysis was conducted following the recommendations of the Evidence-Based Anatomy (EBA) Workgroup [4] and the PRISMA 2020 guidelines [5]. To assess the risk of bias in the included studies, we applied the Anatomical Quality Assurance (AQUA) Tool [6], which consists of 25 items across five domains: (1) study objectives and participant characteristics, (2) study design, (3) methodology characterization, (4) descriptive anatomy, and (5) results reporting. A domain was rated as “low” risk of bias only when all corresponding items were answered “yes”. If any item received a “no”, the entire domain was classified as “high” risk of bias. Two authors (C.T. and N.K.) independently assessed the risk of bias. Disagreements were resolved through discussion, with a third author (G. Tr.) consulted in cases of major discrepancies. The overall quality of each study was graded using the classification proposed by Zappalá et al. [7]: high quality: all five AQUA domains scored as low-risk; moderate quality: three or four domains scored as low-risk; and low quality: fewer than three domains scored as low-risk. The risk of bias across studies was visualized using a weighted bar plot based on sample size. This was generated using the R programming language (version 4.3.3) and RStudio (version 2023.12.1+402) with the robvis package.

Literature Search and Data Extraction. A systematic search was conducted in PubMed, Scopus, Web of Science, and Google Scholar for relevant literature published up to September 2024. Two reviewers (G.Tr. and N.K.) independently screened titles and abstracts, assessed full-text articles, and extracted data. Discrepancies were resolved by consensus with the involvement of other authors where necessary.

Search terms were applied in various combinations and included the following: “foramen magnum”, “occipital condyles”, “skull base”, “cranial base”, “variations”, “morphometry”, “anatomical study”, “osteological study”, “radiological study”, “imaging study”, “Computed Tomography”, and “sexual dimorphism”. A representative PubMed search string is provided below: (“foramen magnum”[MeSH Terms] OR “foramen magnum”[Title/Abstract] OR “occipital bone”[MeSH Terms] OR “occipital bone”[Title/Abstract] OR “occipital condyles”[Title/Abstract] OR “occipital condyle”[Title/Abstract] OR “skull base”[MeSH Terms] OR “skull base”[Title/Abstract] OR “cranial base”[Title/Abstract]) AND (“morphometry”[Title/Abstract] OR “morphometrics”[Title/Abstract] OR “morphometric characteristics”[Title/Abstract] OR “anatomy”[MeSH Terms] OR “anatomy”[Title/Abstract] OR “anatomic variation”[MeSH Terms] OR “anatomical”[Title/Abstract] OR “osteological”[Title/Abstract] OR “radiological”[Title/Abstract] OR “imaging”[Title/Abstract] OR “Multidetector Computed Tomography”[MeSH Terms] OR “Computed Tomography”[Title/Abstract] OR “Sex Characteristics”[MeSH Terms] OR “sexual dimorphism”[Title/Abstract]).

Eligibility Criteria. Studies were included if they reported original morphometric data on the FM and/or OCs in adult human subjects. Inclusion was not restricted by language, geographic origin, or publication date. Both male and female participants were considered. Studies were excluded if they provided insufficient data to compute pooled estimates (i.e., lacking sample size, mean values, or standard deviations). Additional exclusion criteria included case reports, case series, reviews, animal studies, letters to the editor, and conference abstracts. Studies focused on pediatric populations or individuals with cranial anomalies were also excluded to maintain anatomical consistency in adult reference values.

Data Collection and Statistical Analysis. Besides database searches, other sources were explored to identify eligible studies. Grey literature was reviewed, and a manual search of key anatomical journals was performed, including *Annals of Anatomy*, *Journal of Anatomy*, *Anatomical Record*, *Clinical Anatomy*, *Surgical and Radiologic Anatomy*, *Anatomical Science International*, *Morphologie*, *Folia Morphologica*, and *Anatomy & Cell Biology*. Reference lists of all included studies were also screened to identify additional relevant articles. Data extraction was organized using Microsoft Excel. Extracted variables included the following: first author; year of publication; study type (osteological or imaging-based); sample size; participant sex; geographical region (continent); foramen magnum length (FML) and width (FMW); occipital condyle length (OCL), width (OCW), thickness (OCT), and laterality (left or right). Statistical analysis was conducted by a single author (C.T.) using R (version 4.3.3) and RStudio (version 2023.12.1+402). The R packages meta, metafor, and dmetar were employed [8,9,10]. Meta-analyses of untransformed means were performed to estimate pooled values for FML, FMW, OCL, OCW, and OCT. Mean differences (MDs) were analyzed to assess bilateral asymmetry and sex-based differences in OC and FM measurements.

The meta-analyses were conducted using the inverse variance method under a random-effects model, with the restricted maximum-likelihood estimator (REML) used to estimate between-study variance (τ^2^). The Q-profile method calculated confidence intervals for τ and τ^2^ [10]. Heterogeneity was quantified using the Higgins I^2^ statistic and classified as follows: minor (0–24%), low (25–49%), moderate (50–74%), and high (≥75%).

Funnel plot asymmetry was assessed to detect small-study effects, using the linear regression test proposed by Thompson and Sharp [11,12]. Outlier and influence analyses were conducted to identify influential outlier studies (IOSs) [10]. Following the exclusion of these IOSs, meta-analyses were repeated, and the percentage change in pooled estimates was calculated to evaluate their influence.

Subgroup analyses assessed whether study design (osteological vs. imaging) and geographical region influenced pooled estimates or MDs. A meta-CART (classification and regression trees) approach was applied to examine interactions between multiple moderators using the metacart R package [13]. The algorithm included four moderators: sex, side, study design, and geographical region. Only studies with complete data for all four moderators (sample size, mean, and standard deviation) were included in the meta-CART analysis. A pruning parameter of c = 0.5 was used. Statistical significance was set at *p* < 0.05 unless otherwise specified.

## 3. Results and Discussion

### 3.1. Study Identification and Selection

**Figure 2:**
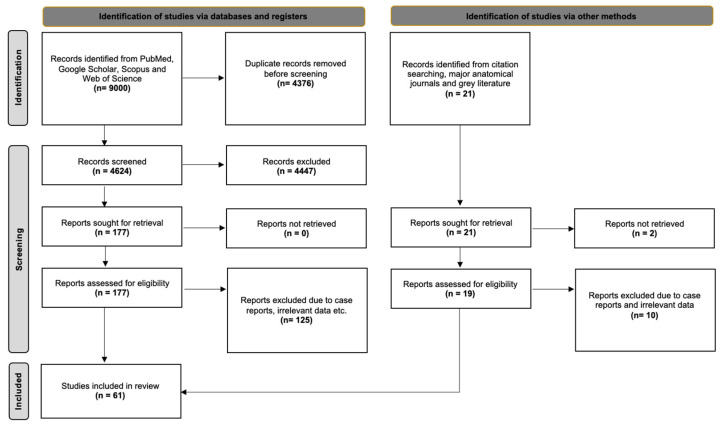
PRISMA 2020 flow diagram illustrating the study selection process. A total of 9000 records were identified through database searches (PubMed, Scopus, Web of Science, and Google Scholar), and 21 additional records were identified via other methods (*citation tracking*, *anatomical journals*, and *grey literature*). After duplication removal and screening, 61 studies met the inclusion criteria and were included in the final systematic review and meta-analysis.

Initial searches in PubMed, Scopus, and Web of Science retrieved over 10,000 records. To maintain feasibility and methodological rigor, only the top 1000 records from each database, ranked by relevance algorithms (e.g., “Best Match” in PubMed), were screened, yielding 3000 articles. Google Scholar also produced high volumes. Given multiple queries, only the first 500 results per search were considered, focusing on the most relevant literature. This process yielded an additional 6000 records. All 9000 records were exported to Mendeley (version 2.10.9, Elsevier, London, UK) for duplicate removal. After deduplication, 4624 unique articles remained and were screened in three stages: *(1) title screening for relevance*, *(2) abstract screening for eligibility*, and *(3) full-text assessment based on predefined inclusion criteria*. Following full-text review, 54 studies met all criteria and were included in the meta-analysis. An additional 21 potentially relevant studies were identified through reference lists, grey literature, and key anatomical journals; seven fulfilled all inclusion criteria. In total, 61 studies were included in the final systematic review and meta-analysis. The study selection process is presented in Figure 2, following the PRISMA 2020 flow diagram.

### 3.2. Studies’ Characteristics

The main characteristics of the included studies are summarized in Table 1. Sixty-one studies, published between 1975 and 2024, were included in the present meta-analysis. Among these, 43 studies were osteological, 16 employed imaging techniques, and 2 [14,15] utilized both imaging and osteological methods. Regarding morphometric focus, 22 studies assessed OCs exclusively, 17 focused on the FM, and 22 analyzed both structures. The sample consisted of 8010 skulls, of which 5496 FMs and 11,006 OCs (from 5503 skulls) were measured. Guidotti’s study [16] notably represented the largest OC sample, measuring 741 skulls (1482 condyles).

**Figure 3:**
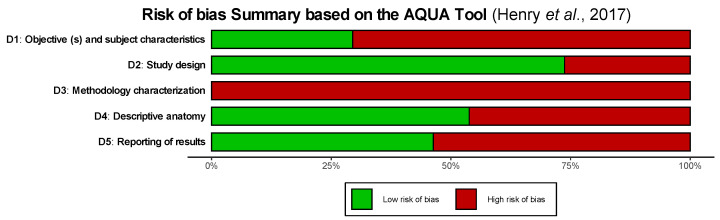
The risk of bias assessment [5].

Geographically, most studies originated from Asia (*n* = 31), followed by Africa (*n* = 12), America (*n* = 8), and Europe (*n* = 8). Two studies (*n* = 2) did not report their country of origin. None of the included studies specified the subjects’ genetic ethnicity or ancestral background. This limitation should be acknowledged, as regional origin may influence morphometric variability; future research should explore the potential association between geography and pooled means or MDs. In terms of data reporting, 33 studies presented overall mean values without distinguishing by side or sex. A total of 25 studies reported sex-specific means using combined bilateral values, 21 studies disaggregated data by both sex and side, and 26 reported side-specific means. Several studies used multiple reporting formats. Regarding methodological quality, 39 studies were rated as low-quality and 22 as moderate-quality; none met the criteria for high quality. This aligns with existing literature, which reports that the Anatomical Quality Assessment (AQUA) Tool tends to identify a high risk of bias in anatomical studies [73,74]. Figure 3 summarizes the risk of bias evaluation using the AQUA Tool [6], weighted by the number of skulls in each study. The figure presents the distribution of risk across the five AQUA domains: Domain 3 (Methodology Characterization): 100% high risk, reflecting a universal lack of transparency in methodology, including absent details on reproducibility, observer variability, and operator expertise. Domain 1 (Objectives and Subject Characteristics): ~70% high risk, indicating poorly described study objectives and subject characteristics, which may limit generalizability. Domain 5 (Reporting of Results): Slightly >50% high risk, due to inconsistencies in statistical reporting and inclusion criteria. Domain 2 (Study Design): ~75% low risk, suggesting that most studies had structurally sound designs. Domain 4 (Descriptive Anatomy): Mixed risk profile, with low risk slightly prevailing, though many studies lacked clarity in anatomical definitions and visual documentation. The analysis highlights major weaknesses in methodological transparency and reporting standards across morphometric studies on OCs and the FM. A high risk of bias was prevalent, particularly in the methodological and reporting domains, highlighting the need for greater transparency and standardization in anatomical morphometric research.

### 3.3. Outcomes of the Statistical Analysis

#### 3.3.1. Pooled Morphometric Means

A significant and high degree of heterogeneity was observed (I^2^ > 75%, *p* < 0.01). The overall results of the pooled means are summarized in Table 2, and the plots are presented in the Supplementary Materials (Supplementary Figures S1–S11).

The bilateral (left and right combined) pooled mean values for the OCs were estimated as follows: OCL 21.51 mm [95% CI: 20.22–22.80], OCW 11.23 mm [10.43–12.03], and OCT 9.11 mm [8.33–9.88]. The regression test for funnel plot asymmetry indicated no small-study effect.

For the left OC, the pooled means were OCL 22.40 mm [21.50–23.30], OCW 12.37 mm [11.81–12.94], and OCT 9.33 mm [8.74–9.91]. A small-study effect was identified only for the pooled mean width (*p* = 0.0467), with no influential outlier detected.

For the right OC, the pooled means were OCL 22.32 mm [21.45–23.19], OCW 12.27 mm [11.72–12.83], and OCT 9.62 mm [8.90–10.34] with no evidence of a small-study effect or the presence of influential outliers.

Based on the test for subgroup differences, study design (osteological vs. imaging-based) was identified as a significant moderator of OC morphometry. Osteological studies consistently reported higher pooled mean values compared to imaging-based studies. Specifically, the mean OCL was 22.82 mm [21.90–23.74] in osteological studies versus 18.55 mm [17.62–19.49] in imaging studies (*p* < 0.0001), and the mean OCW was 11.71 mm [10.93–12.49] and 10.15 mm [9.38–10.92], respectively (*p* = 0.0052). For the left OC, the mean length was 23.13 mm [22.17–24.09] in osteological versus 20.22 mm [18.85–21.59] in imaging studies (*p* = 0.0045), and the mean width was 12.73 mm [11.81–13.64] versus 11.31 mm [10.35–12.27] (*p* = 0.0294). Similarly, the right OCL was greater in osteological studies (23.07 mm [22.19–23.95]) compared to imaging-based studies (19.66 mm [18.37–20.95], *p* = 0.0001). Although the right OCW did not reach conventional significance, a trend was observed at the level of 0.1 (*p*-value < 0.1) (osteological: 12.57 mm [11.52–13.62]; imaging: 11.37 mm [10.22–12.52]; *p* = 0.0861). These findings suggest that the measurement method significantly influences the reported OC dimensions, emphasizing the need to account for study design when interpreting morphometric data.

Subgroup analysis indicated that the geographical region of origin (continent) significantly moderates the OC morphometric outcomes, including overall dimensions and side-specific measurements (OCL, OCW, and OCT of both left and right OCs). However, the number of studies within each subgroup did not meet the minimum threshold of four per subgroup, as recommended for categorical subgroup analysis [75]. Consequently, while these findings point to potential geographic variation in OC morphometry, further studies are needed to validate these observations.

The pooled means for FM dimensions were estimated at 35.02 mm [34.34–35.70] for FML and 28.94 mm [27.52–30.35] for FMW. Heterogeneity was statistically significant and of a high degree, although no small-study effects were detected. IOSs were identified for both FML [28] and FMW [51], potentially biasing the pooled estimates. After excluding these studies, the recalculated means were 35.30 mm [34.82–35.77] for FML and 29.53 mm [28.64–30.43] for FMW, corresponding to a +0.8% and +2.0% increase, respectively.

Subgroup analyses for FML and FMW did not meet the minimum requirement of four studies per subgroup [75], limiting the reliability of these comparisons. Although statistically significant associations were observed between FML and study design and between FMW and geographical region, further research is needed. Study design influenced FML (*p* = 0.0204), with slightly higher means reported in osteological studies than imaging-based ones. After excluding the IOS [28], this association remained significant (*p* < 0.001), strengthening the potential validity of the observed correlation.

#### 3.3.2. Pooled Morphometric Mean Differences (MDs)

The overall results are summarized in Table 3, and the plots are presented as Supplementary Materials (Supplementary Figures S12–S21). Overall, statistically significant (*p*-value < 0.01) MDs between males and females were estimated, with the OC and FM dimensions being greater in males than in females. The most pronounced sex differences were found in the length of both structures. The only comparison that did not reach statistical significance at the 0.05 level was the bilateral estimation of MD for OCW (*p* = 0.0767), although it was significant at the 0.1 level. However, when analyzed separately by side (left and right), the MDs for OCW were significant (*p* < 0.01). The study by Rai et al. [54] was identified as an IOS for the MDs of FML and FMW, OCL (right), and OCW (left and right). After excluding this IOS [54], the re-MDs confirmed the existence of significant differences between sexes. Subgroup analyses revealed a slight tendency toward greater MDs in imaging studies compared to osteological studies for both the OCL and OCW, suggesting a possible superiority of imaging techniques in detecting morphometric sex differences in these structures. The results of the MDs are presented as follows. 

The pooled MDs between left and right OC dimensions (OCL, OCW, and OCT) were not statistically significant. However, after excluding the IOS [69] a significant MD in OCT was observed: −0.11 mm [−0.20 to −0.03], *p* = 0.0060, with no detected heterogeneity (I^2^ = 0.0%). The results suggest a potential difference in the OCT between the left and right sides, with the left OC slightly thinner than the right. However, this difference was not present when the IOS was included.

Sex-based differences in the dimensions of the OCs were evaluated through pooled MDs.

For OCL, significant differences were observed in all analyses. The pooled MDs between males and females were 1.71 mm [1.41–2.01], *p* < 0.0001, I^2^ = 14.6% for the bilateral estimation; 1.91 mm [1.44–2.37], *p* < 0.0001, I^2^ = 97.3% for the left OC; and 2.10 mm [1.57–2.62], *p* < 0.0001, I^2^ = 98.1% for the right OC. After excluding the IOS [54], the right OC MD was recalculated as 1.89 mm [1.53–2.26], *p* < 0.0001, I^2^ = 94.0%, corresponding to a 9.6% decrease. No small-study effects were detected in these comparisons. These findings indicate that males tend to have approximately 2 mm-longer OCs than females.

Regarding OCT, sex differences were also identified. The pooled MDs were 0.71 mm [0.26–1.16], *p* = 0.0018, I^2^ = 82.3% for the bilateral thickness; 0.63 mm [0.31–0.94], *p* < 0.0001, I^2^ = 73.4% for the left OC; and 0.37 mm [0.19–0.55], *p* < 0.0001, I^2^ = 32.9% for the right OC. No small-study effects were detected in thickness-related analyses. The results suggest that males exhibit greater OCT than females, mainly on the left OC, with the difference being less than 1 mm.

In contrast, for OCW, the sex-based difference in the bilateral estimation was not significant (MD: 0.33 mm [–0.04–0.70], *p* = 0.0767, I^2^ = 75.5%). For the left OC, the MD was 0.67 mm [0.20–1.13], *p* = 0.0052, I^2^ = 97.1%, while the re-MD after exclusion of the IOS was 0.51 mm [0.27–0.74], *p* < 0.0001, I^2^ = 89.1%, indicating a 24.1% decrease. For the right OC, the MD was 0.68 mm [0.19–1.17], *p* = 0.0067, I^2^ = 98.9%, and the re-MD was 0.51 mm [0.26–0.77], *p* < 0.0001, I^2^ = 87.5%, representing a 24.9% decrease after excluding the IOS. Statistically significant small-study effects were detected for the right OC width in the MDs and re-MDs (*p* = 0.0050 and *p* = 0.0045, respectively), and for the re-MD of the left OCW (*p* = 0.0133). A marginally non-significant small-study effect was also observed for the MD of the left OCW (*p* = 0.0566). Although the bilateral estimations were not statistically significant, the side-specific analyses revealed sex-based differences in OCW, with males exhibiting wider OCs than females, with the difference being less than 1 mm. While the MDs decreased after excluding IOS, they remained statistically significant. Small-study effects underscore the need for further research with larger sample sizes.

The MD for FML between males and females was 2.21 mm [1.38–3.05], with a re-MD of 1.82 mm [1.33–2.32], showing a 17.77% decrease after excluding the IOS (*p* < 0.0001 for both). For FMW, the MD was 2.02 mm [1.35–2.69], with a re-MD of 1.75 mm [1.25–2.24], representing a 13.29% decrease after excluding the IOS (*p* < 0.0001 for both). MDs and re-MDs were statistically significant, close to 2 mm, with the length estimations slightly higher than those for width. High heterogeneity was observed (I^2^ = 99.3% for length MD, 94.8% for re-MD; I^2^ = 98% for width MD, 92.2% for re-MD). No small-study effect was detected.

The subgroup analyses revealed significant results for the estimated MD between males and females, with study design (osteological vs. imaging) as a moderator, for both the right OCL and right OCW. The significant results of the subgroup analyses with at least four studies per subgroup are reported as follows: for the right OCL, the estimated MD for the subgroup of imaging studies was approximately 2.5735 mm, while for the subgroup of osteological studies, the MD was approximately 1.5398 mm (*p* = 0.0268). For the right OCW, the imaging studies subgroup yielded an MD of approximately 1.1159 mm, and for the osteological studies subgroup, an MD of roughly 0.2019 mm (*p* = 0.0353). The statistical significance of the study’s design as a moderator of the estimated MD between males and females was also confirmed after excluding the IOS [54] and re-conducting the subgroup analyses.

Based on the subgroup analyses, imaging studies are possibly associated with a greater estimation of the MD between males and females for both OCL and OCW compared to osteological studies. These results were limited to the right condyle. However, this correlation was also found to be significant for the bilateral estimation of the OCW (imaging MD = 0.6415 mm, osteological MD = −0.1095 mm, *p* = 0.0002), but without reaching the minimum of four studies per group [75] In addition, this correlation was also found for the left OCL (imaging MD = 2.2590 mm, osteological MD = 1.5114 mm, *p* = 0.0913 < 0.1) and for the left OCW (imaging MD = 0.9990 mm, osteological MD = 0.2732 mm, *p* = 0.0819 < 0.1) but at the significant level of 0.1. Overall, the results suggest a slight trend for a greater MD between males and females for the OCL and OCW in imaging studies compared to osteological studies. However, further research is required to confirm this correlation.

Subgroup analyses of MDs for FML and FMW based on study design yielded no statistically significant results. Regarding evaluation of the subjects’ geographical region (continent of origin) as a moderator of the estimated MD, none of the subgroup analyses met the minimum of four studies per subgroup [75]. Therefore, although significant results were obtained, further studies are required.

#### 3.3.3. Multiple Moderator Analysis with Meta-CART

**Figure 4:**
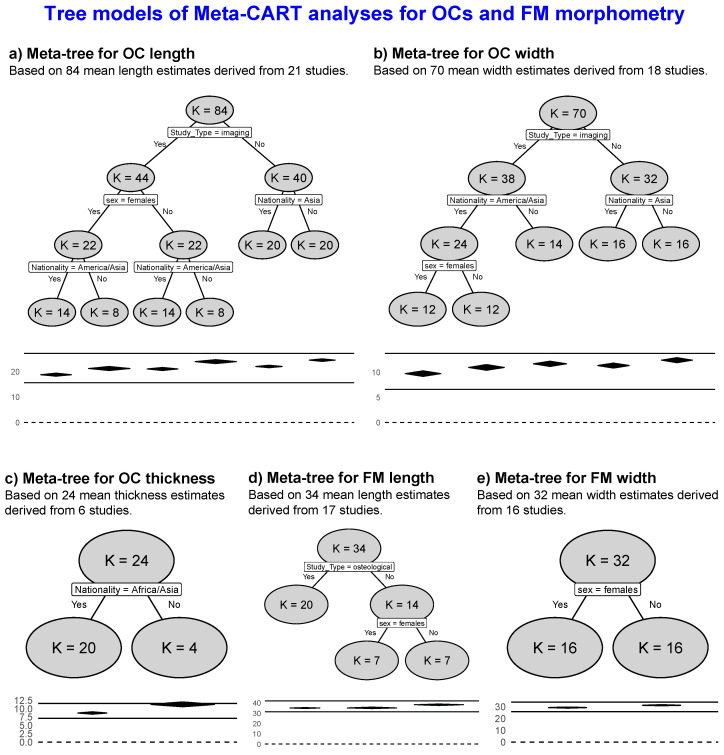
Tree models of the meta-CART analysis of the current meta-analysis.

Meta-CART analysis identified key moderators affecting OCL variability across studies. Eighty-four mean OCL estimates derived from 21 studies were included, resulting in a meta-tree with six terminal nodes (Figure 4a). The analysis detected three statistically significant moderators: study design (imaging vs. osteological), sex, and geographical region (Africa, Asia, America, and Europe), while the anatomical side (left/right) did not contribute significantly to heterogeneity. The test for between-subgroups heterogeneity under the random-effects model was significant (*p* < 0.001), indicating that these moderators account for a substantial portion of the observed variability.

The first and most influential split occurred at the level of study design, suggesting OCL differences between imaging-based and osteological studies. Further divisions by sex and geographical origin revealed distinct subgroup patterns. Notably, sex emerged as a significant moderator only within imaging studies. Pooled means OCLs varied across terminal nodes, ranging from 18.76 mm to 24.46 mm. The highest pooled mean OCL was observed in osteological studies focusing on non-Asian populations, while the lowest was found in imaging studies of American/Asian females. The analysis grouped imaging studies from America and Asia due to similar mean values, suggesting a potentially typical pattern in OCL morphometry across these regions.

Based on 70 mean OCW estimates from 18 studies, the analysis revealed a meta-tree with five terminal nodes (Figure 4b). It detected three significant moderators: study design (imaging vs. osteological), geographical region (Africa, Asia, America, and Europe), and sex. Similar to the findings for OCL, the study design was identified as the primary moderator in the meta-CART analysis of OCW estimates. However, among imaging-based studies, the geographical region was the most influential moderator for OCW, followed by sex, whereas, for OCL, sex was the most critical moderator. These findings align with results from the estimated MDs, where sex-related morphometric differences were more pronounced in length. Within imaging studies, populations from America and Asia clustered separately from other regions, while in osteological studies, the main distinction was between Asian and non-Asian populations. This pattern was consistent with the meta-CART analysis for OCL, highlighting similar regional grouping across both dimensions and suggesting that American and Asian populations share morphometric traits that differ from other groups. Sex did not emerge as a significant moderator of OCW in osteological studies. In contrast, it followed geographical region in importance in imaging studies, possibly reflecting the enhanced sensitivity of imaging techniques in capturing subtle morphometric differences. Overall, based on the meta-trees, sex appeared to have a more minor impact on OCW than on OCL. The test for between-subgroups heterogeneity was statistically significant (*p* < 0.001), indicating that these moderators explained a substantial portion of the observed variability.

Based on 24 mean OCT estimates from six studies, a meta-tree with two terminal nodes was identified (Figure 4c). The geographical region (*Africa, Asia,* and *America*) emerged as the only statistically significant moderator. In the present analysis, which included data from three continents (*Africa, Asia,* and *America*), the meta-tree grouped Africa and Asia separately from America, indicating regional differences in OCT. The test for between-subgroups heterogeneity under the random-effects model was significant (*p* < 0.001), confirming that the two identified clusters (*Africa/Asia* vs. *America*) differed significantly. The estimated mean OCT was 8.71 mm [8.39–9.03] for the Africa/Asia cluster and 11.3 mm [10.6–12] for the American group. These findings suggest that individuals from the American region exhibit greater OCT compared to those from African and Asian populations (*p* < 0.001). Study design, anatomical side, and sex were not significant moderators for OCT.

Based on 34 mean FML estimates derived from 17 studies, a meta-tree with three terminal nodes was identified (Figure 4d), with study design (imaging vs. osteological) and sex emerging as significant moderators (*p* < 0.001). The primary split in the meta-tree was determined by study design, indicating it as the most influential moderator. Among imaging-based studies, sex was found to be a significant moderator, whereas no such effect was observed in osteological studies. This pattern highlights the potential superiority of imaging techniques in detecting subtle morphometric differences compared to osteological methods. In imaging studies, FML was significantly shorter in females compared to males: 35.205 mm [34.467–35.944] in females and 38.259 mm [37.525–38.994] in males, with the difference being significant (*p* < 0.001).

Based on 32 mean FMW estimates derived from 16 studies, a meta-tree with two terminal nodes was detected (Figure 4e), with sex emerging as the only significant moderator (*p* < 0.001). Accordingly, the split in the meta-tree was determined by sex. The results indicated that males have significantly greater FMW compared to females, with mean values of 31.246 mm [30.844–31.647] in males and 29.290 mm [28.888–29.693] in females (*p* < 0.001).

### 3.4. Neurosurgical and Forensic Implications

The pooled morphometric analyses revealed substantial heterogeneity in OC and FM dimensions across populations, underscoring the need for neurosurgeons to exercise caution when operating in this anatomically complex region. The pooled mean values were estimated as follows: 21.51 mm for OCL, 11.23 mm for OCW, 9.11 mm for OCT, 35.02 mm for FML, and 28.94 mm for FMW. The moderator analyses suggest that the study methodology significantly influences morphometric measurements. Notably, osteological studies report higher mean values than imaging-based studies, indicating that the measurement technique substantially affects the observed morphometric outcomes. Despite using relatively stable anatomical landmarks, inherent anatomical variations of the craniovertebral junction, such as differences in FM shape or OC configuration, may have contributed to the observed heterogeneity in morphometric estimates across studies. However, the influence of anatomical variability on the estimated pooled morphometric means could not be directly quantified due to insufficient corresponding data reported in the included studies.

No significant difference in morphometry was found between the left and right OCs. However, a trend toward a slightly thinner left OC than the right was detected, suggesting a potential asymmetry in OCT.

Sex-based differences were also observed, with males exhibiting larger OC and FM dimensions than females. The most pronounced differences were close to 2 mm and were found in the OCL, FML, and FMW. Males also exhibited greater OCT than females, particularly on the left side, with a sex-based mean difference of less than 1 mm. The results indicate that although the left OC appears slightly thinner compared to the right, it shows the most pronounced sex-based difference in thickness, with males exhibiting a greater thickness than females. Similarly, OCW was greater in males, with a difference of under 1 mm. These findings emphasize the importance of sex as a key determinant in craniovertebral morphometry. The association between sex and the morphometric dimensions of the OCs and FM is of particular forensic significance, especially when cranial remains are partially preserved or highly fragmented [3].

Subgroup analyses revealed a slight tendency toward larger MDs between males and females in imaging studies compared to osteological studies, particularly in OCL and OCW. This suggests a possible superiority of imaging techniques in detecting sex-related morphometric differences in these structures. The results of multiple moderator analyses further supported the superiority of imaging modalities. Specifically, the meta-CART analysis confirmed the study design, imaging versus osteological morphometry, as a key moderator influencing the measurement of OC and FM dimensions. Sex emerged as a significant moderator within imaging studies but not in osteological studies, indicating that imaging techniques may be more sensitive in capturing subtle morphometric variations.

This advantage of imaging-based studies may be attributed to the higher measurement precision provided by high-resolution 3D reconstruction imaging, which allows for accurate landmark identification, automated alignment, and measurement within the native anatomical context. These techniques minimize errors caused by bone degradation and suboptimal positioning, which can obscure subtle sex differences in osteological samples. Future advances in imaging analysis, such as incorporating artificial intelligence (AI)-assisted techniques, may further enhance the detection of morphometric differences. To that end, AI models, such as convolutional neural networks (CNNs) and transformer-based architectures, offer a promising avenue for morphometric analysis. These models can outperform traditional manual or semi-automated methods by providing highly accurate, consistent, and reproducible measurements while minimizing observer-dependent variability. CNNs can automatically learn complex spatial hierarchies and localized anatomical features from imaging data, whereas transformers can capture broader spatial relationships and long-range dependencies across structures. As a result, AI models are more sensitive to minute morphological differences that might otherwise go undetected and can also systematically extract patterns linked to sex, ancestry, or pathological conditions. These tools could become particularly valuable in neurosurgical contexts, where even minor anatomical variations can have significant clinical implications, especially in craniovertebral surgery [76,77,78,79].

Geographical differences were evident, suggesting distinct population-specific morphometric profiles. Further research involving larger sample sizes from a broader range of regions is necessary to improve our understanding of the influence of geographical origin on OC and FM morphometry. The investigation of the influence of geographical origin and sex on morphometric values has significant applications in forensic science, particularly in identifying individuals in cases involving skeletal remains [3].

The recent literature demonstrates a growing interest in morphometric variations of the FM and OCs, focusing on their relevance to inter-population differences, sex estimation, forensic identification, and clinical implications. Muley and Muley [80] conducted a comparative study on FM dimensions, underscoring its significance in forensic identification and biomechanics. Misra and Bateja [81] employed CBCT imaging to investigate sex-related differences in FM size, confirming its potential utility in sex determination. Similarly, Femi-Akinlosotu et al. [82] conducted a population-based study on Nigerian skulls, highlighting inter-population differences in FM dimensions. In a clinical context, Chuang et al. [83] performed a multidimensional analysis of FM dimensions in patients with Chiari Malformation Type I, revealing morphometric and volumetric differences associated with clinical symptoms. Thornton et al. [84] employed geometric morphometric analysis to assess FM shape variants and their implications for growth patterns and evolutionary changes. In addition, Alpergin et al. [85] analyzed FM size concerning posterior cranial fossa abnormalities, particularly in pediatric patients.

Scadorwa and Wierzbieniec [86] noted that the FM morphology is altered in craniosynostoses such as brachycephaly and Crouzon syndrome. However, no studies have specifically examined FM morphology and morphometry in scaphocephaly, the most common cranial deformity caused by premature sagittal suture fusion. In scaphocephaly, the dolichotrematous FM type was predominant in 58.9% of cases. The mean FM area was 519.64 mm^2^, significantly smaller than that of the control groups. The TD and APD were also markedly reduced. This reduction is primarily linked to a considerable decrease in FM width in children with sagittal craniosynostosis. Interestingly, the FM in scaphocephaly appears larger than in previously reported cases of brachycephaly or Crouzon syndrome [86].

Neurosurgeons should remember that FM dimensions are shorter in females than in males; thus, a shorter operating field is offered, and extensive bone extraction might be essential [49]. Nevertheless, OC is more frequently implicated in surgical procedures. Posterior surgical approaches (*transcondylar*, *supracondylar*, and *paracondylar*) are used for tumor removal. A key step in these approaches is drilling around and/or at the OC area [61]. Partial condylectomy could lead to greater atlanto-occipital instability postoperatively, especially in cases with a short OC length [61]. Elongated OCs may need extensive resection to achieve better intraoperative visualization. Nevertheless, OCT is essential for vertical drilling and the safety of critical surrounding structures, such as the hypoglossal nerve [61].

## 4. Limitations

A key limitation of this meta-analysis was the high volume of initial search results, necessitating algorithmic relevance rankings to screen only the top records from each database. While this approach improved feasibility, it may have resulted in the exclusion of relevant studies. To address this, supplementary methods, such as backward reference searching, enhanced coverage, and reduced selection bias could be applied in future studies. Another limitation was the absence of data on participants’ genetic ethnicity or ancestral background across the included studies. As such, further research is needed to explore potential associations between geographical origin and the pooled morphometric means and mean differences (MDs). The most critical limitations were the substantial heterogeneity across studies and the high risk of bias identified by the AQUA Tool. These factors limit the certainty of the pooled estimates and underscore the need for more standardized, high-quality morphometric research.

## 5. Conclusions

This meta-analysis underscores the substantial morphometric variability of the OCs and FM, shaped by measurement technique, sex, geographic origin, and anatomical side. Imaging-based studies exhibited greater sensitivity in detecting subtle anatomical differences, particularly sex-based, than osteological methods. These findings have important clinical and forensic implications, notably in neurosurgical planning and identifying fragmented cranial remains. Future research should prioritize large-scale, cross-population comparisons, incorporate 3D morphometric modeling, and explore AI-assisted imaging analysis for more robust and automated morphometric assessments to advance precision and applicability.

## Figures and Tables

**Table 1:** The meta-analysis includes the studies’ main characteristics, risk of bias, quality assessment, and outcome data.

#	Study	Year	Risk of Bias * D1/D2/D3/D4/D5	Quality	Morphometry	Nationality	Study Type	No. of Skulls	Estimated Mean	No. of OCs	Νο. of FMs
1	Abdel-Karim et al. [17]	2015	✖/✔/✖/✔/✖	Low	OCs and FM	Africa	Imaging	70	per sex and side	140	
									per sex	140	70
2	Ads et al. [18]	2021	✖/✔/✖/✔/✔	Moderate	OCs	Africa	Imaging	48	per sex and side	96	
3	Aljarrah et al. [3]	2021	✖/✔/✖/✔/✔	Moderate	OCs and FM	Asia	Imaging	472	per sex	854	427
									per sex and side	854	
4	Anjum et al. [19]	2021	✖/✔/✖/✔/✔	Moderate	OCs and FM	Asia	Osteological	100	per sex	200	100
									per sex and side	200	
5	Aristotle et al. [14]	2020	✖/✔/✖/✔/✖	Low	OCs and FM	Asia	Osteological	70	οverall		70
									per side	140	
							Imaging	70	οverall		70
									per side	140	
6	Avci et al. [15]	2011	✖/✔/✖/✔/✖	Low	OCs and FM	Asia	Osteological	30	οverall		30
									per side	60	
							Imaging	30	per side	60	
7	Bayat et al. [20]	2014	✖/✖/✖/✖/✖	Low	OCs	Asia	Osteological	50	οverall	95	
									per side	95	
8	Berge and Bergman [21]	2001	✖/✔/✖/✖/✖	Low	FM	Unknown	Osteological	100	οverall		100
9	Bernstein et al. [22]	2022	✔/✔/✖/✔/✔	Moderate	OCs	America	Imaging	250	οverall	500	
									per side	500	
									per sex and side	500	
10	Bosco et al. [23]	2018	✔/✔/✖/✔/✔	Moderate	OCs	Asia	Imaging	70	οverall	140	
									per sex	140	
11	Bozbuga et al. [24]	1999	✖/✔/✖/✖/✖	Low	OCs	Asia	Osteological	84	οverall	168	
12	Burdan et al. [25]	2012	✔/✔/✖/✔/✔	Moderate	FM	Europe	Imaging	313	per sex		313
13	Catalina-Herrera [26]	1987	✖/✖/✖/✖/✖	Low	FM	Europe	Osteological	100	per sex		100
14	Cheruiyot et al. [27]	2018	✖/✔/✖/✔/✔	Moderate	OCs	Africa	Osteological	52	οverall	104	
									per side	104	
									per sex	104	
15	Chetnan et al. [28]	2012	✖/✔/✖/✔/✖	Low	FM	Asia	Osteological	53	οverall		53
16	Degno et al. [29]	2019	✖/✔/✖/✖/✖	Low	OCs and FM	Africa	Osteological	54	οverall		54
									per side	108	
17	Dubey et al. [30]	2017	✖/✔/✖/✔/✖	Low	OCs and FM	Asia	Osteological	80	per sex		80
									per sex and side	160	
18	El-Barrany et al. [31]	2016	✖/✔/✖/✔/✔	Moderate	OCs and FM	Africa	Imaging	400	per sex		400
									per sex and side	800	
19	El-Gaidi et al. [32]	2014	✖/✔/✖/✔/✖	Low	OCs	Africa	Osteological	50	οverall	100	
									per side	100	
20	Espinoza et al. [33]	2011	✖/✖/✖/✔/✖	Low	FM	America	Imaging	100	per sex		100
21	Farid and Fattah [34]	2018	✖/✔/✖/✔/✖	Low	OCs and FM	Africa	Osteological	75	οverall	150	75
									per side	150	
22	Fetouh and Awadalla [35]	2009	✖/✔/✖/✔/✔	Moderate	OCs and FM	Africa	Osteological	100	οverall		100
									per side	200	
23	Gapert et al. [36]	2009	✔ / ✔ / ✖ / ✔ / ✔	Moderate	OCs	Europe	Osteological	146	per sex and side	292	
24	George et al. [37]	2019	✖ / ✔ / ✖ / ✖ / ✖	Low	OCs	Asia	Osteological	30	per side	60	
25	Govsa et al. [38]	2011	✖/✔/✖/✔/✔	Moderate	FM	Asia	Osteological	144	οverall		144
26	Guidotti [16]	1984	✖ / ✖ / ✖ / ✖ / ✖	Low	OCs	Europe	Osteological	741	per sex and side	1482	
27	Gummusoy and Duman [39]	2019	✔/✔/✖/✔/✔	Moderate	OCs	Asia	Imaging	100	οverall	200	
									per side	200	
									per sex	200	
									per sex and side	200	
28	Hendricks et al. [40]	2024	✖ / ✖ / ✖ / ✖ / ✖	Low	OCs and FM	Africa	Osteological	50	οverall	100	50
									per side	100	
29	Kalthur et al. [41]	2014	✔/✔/✖/✔/✔	Moderate	OCs	Asia	Osteological	71	οverall	142	
									per sex and side	142	
30	Kavitha et al. [42]	2013	✖/✔/✖/✖/✖	Low	OCs	Asia	Osteological	145	per side	290	
31	Kizilkanat et al. [43]	2006	✖ / ✖ / ✖ / ✖ / ✖	Low	OCs and FM	Asia	Osteological	59	οverall	118	59
									per side	118	
32	Lyrtzis et al. [44]	2017	✔/✔/✖/✔/✔	Moderate	OCs and FM	Europe	Osteological	141	οverall		141
									per side	282	
									per sex and side	282	
33	Manoel et al. [45]	2009	✔/✔/✖/✔/✔	Moderate	FM	America	Osteological	215	per sex		215
34	Murshed et al. [46]	2003	✔/✔/✖/✔/✔	Moderate	FM	Asia	Imaging	110	per sex		110
35	Muthukumar et al. [47]	2005	✖/✔/✖/✖/✖	Low	OCs and FM	Asia	Osteological	50	οverall	100	50
36	Naderi et al. [48]	2005	✔ / ✔ / ✖ / ✔ / ✔	Moderate	OCs and FM	Asia	Osteological	202	οverall	404	202
									per side	404	
37	Natsis et al. [49]	2013	✔/✔/✖/✔/✔	Moderate	OCs and FM	Europe	Osteological	143	οverall		143
									per side	286	
									per sex		143
									per sex and side	286	
38	Oliveira et al. [50]	2013	✖ / ✔ / ✖ / ✖ / ✖	Low	OCs	America	Osteological	100	per sex and side	200	
39	Olivier [51]	1975	✖ / ✖ / ✖ / ✖ / ✖	Low	OCs and FM	Europe	Osteological	125	οverall	250	125
40	Osunwoke et al. [52]	2012	✖ / ✔ / ✖ / ✔ / ✔	Moderate	FM	Africa	Osteological	120	οverall		120
41	Pal et al. [53]	2019	✖ / ✔ / ✖ / ✖ / ✖	Low	OCs	Asia	Osteological	150	per side	300	
42	RaghavendraBabu et al. [2]	2012	✖/✔/✖/✔/✔	Moderate	FM	Asia	Osteological	90	per sex		90
43	Rai et al. [54]	2017	✖/✔/✖/✖/✖	Low	OCs and FM	Asia	Imaging	200	per sex		200
									per sex and side	400	
44	Routal et al. [55]	1984	✖ / ✖ / ✖ / ✖ / ✖	Low	FM	Asia	Osteological	141	per sex		141
45	Salih et al. [56]	2014	✔ / ✔ / ✖ / ✖ / ✖	Low	OCs and FM	Africa	Imaging	123	οverall		123
									per side	246	
									per sex and side	246	
46	Saluja et al. [57]	2016	✖/✔/✖/✖/✖	Low	OCs	Asia	Osteological	114	οverall	228	
									per side	228	
47	Saralaya et al. [58]	2012	✖ / ✖ / ✖ / ✖ / ✖	Low	OCs	Asia	Osteological	70	οverall	140	
									per side	140	
48	Sayee et al. [59]	1987	✖ / ✖ / ✖ / ✖ / ✖	Low	FM	Asia	Osteological	350	per sex		350
49	Sholapurkar et al. [60]	2017	✖ / ✖ / ✖ / ✖ / ✖	Low	OCs	Asia	Osteological	100	per sex and side	200	
50	Siddiqui et al. [61]	2022	✖/✔/✖/✖/✖	Low	OCs and FM	America	Osteological	30	οverall	60	30
									per side	60	
51	Srivastava et al. [62]	2017	✔/✔/✖/✖/✖	Low	OCs	Asia	Imaging	41	οverall	82	
									per side	82	
									per sex	82	
									per sex and side	82	
52	Suazo et al. [63]	2009	✖ / ✖ / ✖ / ✖ / ✖	Low	FM	America	Osteological	211	per sex		211
53	Thintharua and Chentanez [64]	2023	✔/✔/✖/✔/✔	Moderate	OCs	Asia	Osteological	100	οverall	200	
									per sex	200	
									per sex and side	200	
54	Tubbs et al. [65]	2010	✖/✔/✖/✖/✖	Low	FM	Europe	Osteological	72	οverall		72
55	Ukoha et al. [66]	2011	✖/✔/✖/✖/✖	Low	FM	Africa	Osteological	100	per sex		100
56	Uthman et al. [67]	2011	✖/✔/✖/✖/✖	Low	FM	Unknown	Imaging	88	per sex		88
57	Uysal et al. [68]	2005	✖/✔/✖/✖/✖	Low	FM	Asia	Osteological	100	per sex		100
58	Verma et al. [69]	2016	✖/✔/✖/✔/✖	Low	OCs	Asia	Osteological	50	per side	100	
59	Wanebo et al. [70]	2001	✖/✔/✖/✖/✖	Low	OCs and FM	America	Osteological	38	οverall	76	38
60	Yu et al. [71]	2015	✔ / ✔ / ✖ / ✖ / ✖	Low	OCs	Asia	Imaging	20	per sex and side	40	
61	Zanutto et al. [72]	2020	✔ / ✔ / ✖ / ✔ / ✔	Moderate	OCs and FM	America	Imaging	309	per sex		309
									per sex and side	618	

* Risk of bias based on the AQUA Tool [5]; D1, Domain 1: objective(s) and subject characteristics; D2, Domain 2: study design; D3, Domain 3: methodology characterization; D4, Domain 4: descriptive anatomy; D5, Domain 5: reporting of results; ✔, Low risk; ✖, High risk; per sex, male and female; per side, right and left.

**Table 2:** Meta-analysis results for the morphometric mean values of the occipital condyles (OCs) and the foramen magnum (FM). All morphometric mean values are expressed in millimeters (mm). Statistically significant results are depicted with bold letters.

Overall Estimation		Subgroup Analyses					
#	Mean [95%-CI] Heterogeneity: I^2^ Small-Study Effect (SSE) Influential Outlier Study (IOS)	Moderator	Subgroups	k	Mean [95%-CI]	Heterogeneity: I^2^	*p*-Value of Test for Subgroup Differences
1	OC Length 21.5081 [20.2170; 22.7991] I^2^ = 99.5% [99.5%; 99.6%] SSE: *p*-value = 0.1081 IOS: none	Continent of origin	America	2	20.7872 [16.4754; 25.0990]	99.4%	0.0158
			Asia	8	21.1932 [19.4751; 22.9113]	99.5%	
			Africa	2	22.3826 [18.8450; 25.9203]	98.7%	
			Europe	1	23.7500 [23.4104; 24.0896]	--	
		**Study’s design**	**imaging**	**4**	**18.5543 [17.5809; 19.5276]**	**97.2%**	**<0.0001**
			**osteological**	**9**	**22.8223 [21.9595; 23.6851]**	**97.7%**	
2	OC Width 11.2299 [10.4276; 12.0322] I^2^ = 99.2% [99.0%; 99.3%] SSE: *p*-value = 0.4199 IOS: none	Continent of origin	Asia	9	10.8433 [9.9630; 11.7236]	99.2%	<0.0001
			America	1	10.5000 [10.3948; 10.6052]	--	
			Africa	2	13.2098 [11.2793; 15.1404]	98.7%	
			Europe	1	11.5000 [11.3512; 11.6488]	--	
		**Study’s design**	**osteological**	**9**	**11.7117 [10.7091; 12.7144]**	**99.2%**	**0.0052**
			**imaging**	**4**	**10.1543 [9.7205; 10.5880]**	**95.5**	
3	OC Thickness 9.1061 [8.3275; 9.8848] I^2^ = 99.6% [99.5%; 99.6%] SSE: *p*-value = 0.5070 IOS: none	Continent of origin	Asia	7	8.6229 [7.9836; 9.2621]	98.6%	<0.0001
			America	1	11.4000 [11.2861; 11.5139]	--	
			Africa	2	9.6680 [7.6591; 11.6769]	98.8%	
		Study’s design	osteological	7	8.7771 [7.9163; 9.6380]	98.8%	0.2169
			imaging	3	9.8697 [8.3641; 11.3754]	99.6%	
4	FM Length 35.0221 [34.3424; 35.7018] I^2^ = 94.8% [93.0%; 96.2%] SSE: *p*-value = 0.3637 IOS: “Chethan_2012”	Continent of origin	Asia	7	34.6175 [32.9976; 36.2373]	97.7%	0.3221
			Africa	6	35.0909 [34.5327; 35.6490]	84.1%	
			Europe	3	35.4117 [35.0140; 35.8093]	54.4%	
			America	1	36.0000 [35.0462; 36.9538]	--	
		Study’s design	osteological	15	35.1419 [34.3901; 35.8938]	95.1%	0.0204
			imaging	2	34.1199 [33.6946; 34.5452]	0.0%	
	*Re-estimation after excluding the IOS: “Chethan_2012”*						
	FM Length *35.2959 [34.8175; 35.7744]* *I^2^ = 89.9% [85.2%; 93.1%]* *SSE: p-value = 0.2837*	*Continent* *of origin*	*Asia*	*6*	*35.2616 [33.9997; 36.5234]*	*95.4%*	*0.4390*
			*Africa*	*6*	*35.0909 [34.5327; 35.6490]*	*84.1%*	
			*Europe*	*3*	*35.4117 [35.0140; 35.8093]*	*54.4%*	
			*America*	*1*	*36.0000 [35.0462; 36.9538]*	*--*	
		*Study’s design*	*osteological*	*14*	*35.4730 [34.9970; 35.9491]*	*88.9%*	*<0.0001*
			*imaging*	*2*	*34.1199 [33.6946; 34.5452]*	*0.0%*	
*5*	FM Width 28.9364 [27.5202; 30.3526] I^2^ = 99.4% [99.3%; 99.5%] SSE: *p*-value = 0.9082 IOS: “Olivier_1975”	Continent of origin	Asia	6	29.2409 [27.2463; 31.2356]	98.6%	0.0022
			Africa	5	29.3470 [28.7042; 29.9898]	72.5%	
			Europe	3	27.1122 [20.9630; 33.2614]	99.9%	
			America	1	31.0000 [30.3641; 31.6359]	--	
		Study’s design	osteological	13	28.9621 [27.3258; 30.5983]	99.5%	0.8631
			imaging	2	28.7817 [27.5471; 30.0162]	91.2%	
	*Re-estimation after excluding the IOS: “Olivier_1975”*						
	FM Width *29.5317 [28.6352; 30.4282]* *I^2^ = 96.8% [95.7%; 97.6%]* *SSE: p-value = 0.2929*	*Continent* *of origin*	*Asia*	*6*	*29.2409 [27.2463; 31.2356]*	*98.6%*	*0.0032*
			*Africa*	*5*	*29.3470 [28.7042; 29.9898]*	*72.5%*	
			*Europe*	*2*	*30.2482 [29.9297; 30.5668]*	*0.0%*	
			*America*	*1*	*31.0000 [30.3641; 31.6359]*	*--*	
		*Study’s design*	*osteological*	*12*	*29.6587 [28.6352; 30.6821]*	*96.9%*	*0.2838*
			*imaging*	*2*	*28.7817 [27.5471; 30.0162]*	*91.2%*	
6	OC Length (Left) 22.3982 [21.4997; 23.2967] I^2^ = 99.5% [99.4%; 99.5%] SSE: *p*-value = 0.1321 IOS: none	Continent of origin	Asia	14	22.0987 [21.0366; 23.1607]	99.1%	<0.0001
			America	1	18.6000 [18.4510; 18.7490]	--	
			Africa	7	22.9035 [21.1630; 24.6440]	99.0%	
			Europe	2	24.6299 [22.7288; 26.5311]	98.7%	
		**Study’s design**	**osteological**	**18**	**23.1296 [22.3342; 23.9251]**	**97.8%**	**0.0045**
			**imaging**	**6**	**20.2217 [18.3783; 22.0652]**	**99.2%**	
7	OC Length (Right) 22.3209 [21.4481; 23.1937] I^2^ = 99.4% [99.4%; 99.5%] SSE: *p*-value = 0.2777 IOS: none	Continent of origin	Asia	13	21.9779 [20.8525; 23.1034]	99.1%	<0.0001
			America	1	18.7000 [18.5510; 18.8490]	--	
			Africa	7	22.8120 [21.4691; 24.1548]	98.2%	
			Europe	2	24.6297 [22.7286; 26.5309]	98.5%	
		**Study’s design**	**osteological**	**18**	**23.0680 [22.3494; 23.7865]**	**97.7%**	**0.0001**
			**imaging**	**5**	**19.6593 [18.0760; 21.2426]**	**99.1%**	
8	OC Width (Left) 12.3730 [11.8102; 12.9358] I^2 = 99.3% [99.3%; 99.4%] SSE: *p*-value = 0.0467 IOS: none	Continent of origin	Asia	14	11.9937 [11.2558; 12.7316]	99.3%	<0.0001
			America	1	10.4000 [10.3036; 10.4964]	--	
			Africa	7	13.3975 [12.6732; 14.1219]	97.8%	
			Europe	2	12.4282 [11.2914; 13.5649]	98.3%	
		**Study’s design**	**osteological**	**18**	**12.7265 [12.1511; 13.3020]**	**98.9%**	**0.0294**
			**imaging**	**6**	**11.3146 [10.1821; 12.4470]**	**99.4%**	
9	OC Width (Right) 12.2715 [11.7169; 12.8260] I^2^ = 99.4% [99.3%; 99.5%] SSE: *p*-value = 0.2743 IOS: none	Continent of origin	Asia	14	11.8729 [11.1402; 12.6056]	99.3%	<0.0001
			America	1	10.5000 [10.3948; 10.6052]	--	
			Africa	7	13.2796 [12.5813; 13.9779]	96.8%	
			Europe	2	12.4318 [11.1382; 13.7254]	99.3%	
		Study’s design	osteological	18	12.5728 [12.0085; 13.1372]	98.9%	0.0861
			imaging	6	11.3721 [10.1227; 12.6216]	99.6%	
10	OC Thickness (Left) 9.3255 [8.7375; 9.9136] I^2^ = 99.1% [98.9%; 99.3%] SSE: *p*-value = 0.1359 IOS: none	Continent of origin	Asia	6	8.8000 [8.2886; 9.3114]	88.8%	<0.0001
			America	1	11.2000 [11.0861; 11.3139]	--	
			Africa	3	9.5411 [8.5053; 10.5769]	97.2%	
			Europe	1	10.0300 [9.8608; 10.1992]	--	
		Study’s design	osteological	9	9.1529 [8.5956; 9.7102]	96.2%	0.4041
			imaging	2	10.1009 [7.9449; 12.2568]	99.8%	
11	OC Thickness (Right) 9.6186 [8.9022; 10.3350] I^2^ = 99.3% [99.1%; 99.4%] SSE: *p*-value = 0.4233 IOS: none	Continent of origin	Asia	6	9.2055 [8.1967; 10.2144]	98.4%	<0.0001
			America	1	11.4000 [11.2861; 11.5139]	--	
			Africa	3	9.6856 [8.4679; 10.9033]	98.2%	
			Europe	1	10.0900 [9.8963; 10.2837]	--	
		Study’s design	osteological	9	9.4767 [8.7094; 10.2440]	98.3%	0.5233
			imaging	2	10.2520 [7.9981; 12.5060]	99.7%	

k, Number of studies combined; 95%-CI, 95% confidence interval; I^2^, Higgins I^2^ statistic; SSE, Small-Study Effect (test of funnel plot asymmetry); IOS, Influential outlier study; bold font indicates the statistically significant results of subgroup analyses with at least four studies per subgroup; italic font indicates the results of re-estimation after excluding the Influential outlier studies.

**Table 3:** Meta-analysis results for the morphometric mean differences (MDs) of the occipital condyles (OCs) and foramen magnum (FM). All morphometric MDs are expressed in millimeters (mm). Statistically significant results are depicted with bold letters.

Overall Estimation		Subgroup Analyses					
#	Mean Difference [95%-CI] *p*-Value of Mean Difference Heterogeneity: I^2^ Small-Study Effect (SSE) Influential Outlier Study (IOS)	Moderator	Subgroups	k	Mean Difference [95%-CI]	Heterogeneity: I^2^	*p*-Value of the Test for Subgroup Differences
1	OC Length: Left vs. Right −0.0326 [−0.2146; 0.1494] *p*-value = 0.7254 I^2^ = 61.8% [40.0%; 75.7%] SSE: *p*-value = 0.2154 IOS: “Salih_2014”	Continent of origin	Asia	13	−0.0227 [−0.2211; 0.1757]	24.9%	0.9261
			America	1	−0.1000 [−0.3107; 0.1107]	--	
			Africa	7	0.0333 [−0.5021; 0.5687]	84.4%	
			Europe	2	0.0000 [−0.3235; 0.3235]	0.0%	
		Study’s design	osteological	18	0.0130 [−0.1524; 0.1785]	22.8%	0.4734
			imaging	5	−0.1811 [−0.6852; 0.3231]	87.6%	
	Re-estimation after excluding the IOS: “Salih_2014”						
	*OC Length: Left vs. Right* *0.0185 [−0.1136; 0.1506]* *p-value = 0.7836* *I^2^ = 16.9% [0.0%; 50.3%]* *SSE: p-value = 0.3144*	*Continent* *of origin*	*Asia*	*13*	*−0.0227 [−0.2211; 0.1757]*	*24.9%*	*0.2817*
			*America*	*1*	*−0.1000 [−0.3107; 0.1107]*	*--*	
			*Africa*	*6*	*0.2422 [−0.0362; 0.5207]*	*7.4%*	
			*Europe*	*2*	*0.0000 [−0.3235; 0.3235]*	*0.0%*	
		*Study’s design*	*osteological*	*18*	*0.0130 [−0.1524; 0.1785]*	*22.8%*	*0.8593*
			*imaging*	*4*	*0.0390 [−0.1958; 0.2738]*	*8.0%*	
2	OC Width: Left vs. Right 0.0598 [−0.0580; 0.1775] *p*-value = 0.3198 I^2^ = 68.8% [52.5%; 79.5%] SSE: *p*-value = 0.0118 IOS: none	Continent of origin	Asia	14	0.0988 [ 0.0088; 0.1888]	0.0%	0.1340
			America	1	−0.1000 [−0.2427; 0.0427]	--	
			Africa	7	0.0867 [−0.3157; 0.4892]	88.3%	
			Europe	2	−0.0011 [−0.1838; 0.1816]	0.0%	
		Study’s design	osteological	18	0.0807 [−0.0011; 0.1625]	21.0%	0.3157
			imaging	6	−0.0747 [−0.3671; 0.2177]	87.3%	
3	OC Thickness: Left vs. Right −0.3008 [−0.6837; 0.0821] *p*-value = 0.1236 I^2^ = 91.9% [87.5%; 94.7%] SSE: *p*-value = 0.8154 IOS: “Verma_2016”	Continent of origin	Asia	6	−0.4422 [−1.1539; 0.2696]	95.7%	0.6033
			America	1	−0.2000 [−0.3611; −0.0389]	--	
			Africa	3	−0.0879 [−0.2866; 0.1107]	0.0%	
			Europe	1	−0.0600 [−0.3173; 0.1973]	--	
		Study’s design	osteological	9	−0.3361 [−0.8112; 0.1389]	93.5%	0.5229
			imaging	2	−0.1747 [−0.3141; −0.0354]	0.0%	
	Re-estimation after excluding the IOS: “Verma_2016”						
	*OC Thickness: Left vs. Right* *−0.1149 [−0.1969; −0.0330]* *p-value = 0.0060* *I^2^ = 0.0% [0.0%; 62.4%]* *SSE: p-value = 0.8255*	*Continent* *of origin*	*Asia*	*5*	*−0.0897 [−0.2093; 0.0299]*	0.0%	*0.6851*
			*America*	*1*	*−0.2000 [−0.3611; −0.0389]*	*--*	
			*Africa*	*3*	*−0.0879 [−0.2866; 0.1107]*	*0.0%*	
			*Europe*	*1*	*−0.0600 [−0.3173; 0.1973]*	*--*	
		*Study’s design*	*osteological*	*8*	*−0.0833 [−0.1846; 0.0181]*	*0.0%*	*0.2980*
			*imaging*	*2*	*−0.1747 [−0.3141; −0.0354]*	*0.0%*	
4	OC Length: Males vs. Females 1.7063 [1.4052; 2.0074] *p*-value < 0.0001 I^2^ = 14.6% [0.0%; 82.2%] SSE: k* = 5 < 10 (k.min = 10) IOS: none	Continent of origin	Asia	4	1.6071 [1.2952; 1.9190]	0.0%	0.1257
			Africa	1	2.1800 [1.5164; 2.8436]	--	
		Study’s design	imaging	3	1.5902 [1.1800; 2.0003]	14.5%	0.4127
			osteological	2	1.8831 [1.3149; 2.4513]	34.2%	
5	OC Width: Males vs. Females 0.3339 [−0.0358; 0.7037] *p*-value = 0.0767 I^2^ = 75.5% [40.0%; 90.0%] SSE: k* = 5 < 10 (k.min = 10) IOS: none	Continent of origin	Asia	4	0.4000 [−0.0344; 0.8343]	79.4%	0.2869
			Africa	1	0.0400 [−0.4601; 0.5401]	--	
		Study’s design	imaging	3	0.6415 [ 0.4023; 0.8808]	0.0%	0.0002
			osteological	2	−0.1095 [−0.4166; 0.1976]	0.0%	
6	OC Thickness: Males vs. Females 0.7107 [0.2647; 1.1567] *p*-value = 0.0018 I^2^ = 82.3% [54.5%; 93.1%] SSE: k* = 4 < 10 (k.min = 10) IOS: none	Continent of origin	Asia	3	0.7184 [0.0900; 1.3468]	88.1%	0.9822
			Africa	1	0.7100 [0.3198; 1.1002]	--	
		Study’s design	imaging	2	0.9980 [ 0.4100; 1.5859]	73.2%	0.1561
			osteological	2	0.4399 [−0.0590; 0.9388]	75.5%	
7	FM Length: Males vs. Females 2.2145 [1.3813; 3.0477] *p*-value < 0.0001 I^2^ = 99.3% [99.1%; 99.4%] SSE: *p*-value = 0.9388 IOS: “Rai_2017”	Continent of origin	Africa	3	2.1933 [0.6144; 3.7722]	50.5%	0.4315
			Asia	8	2.8364 [1.1942; 4.4786]	98.9%	
			Europe	3	1.8321 [1.5953; 2.0689]	0.0%	
			America	3	1.1800 [0.2815; 2.0785]	94.8%	
			Unknown	1	2.0000 [1.1641; 2.8359]	--	
		Study’s design	imaging	8	2.8564 [1.4335; 4.2793]	99.2%	0.1490
			osteological	10	1.6417 [0.8074; 2.4761]	96.3%	
	Re-estimation after excluding the IOS: “Rai_2017”						
	*FM Length: Males vs. Females* *1.8209 [1.3266; 2.3152]* *p-value < 0.0001* *I^2^ = 94.8% [93.0%; 96.2%]* *SSE: p-value = 0.2193*	*Continent* *of origin*	*Africa*	*3*	*2.1933 [0.6144; 3.7722]*	*50.5%*	*0.6131*
			*Asia*	*7*	*2.1295 [1.0710; 3.1880]*	*86.0%*	
			*Europe*	*3*	*1.8321 [1.5953; 2.0689]*	*0.0%*	
			*America*	*3*	*1.1800 [0.2815; 2.0785]*	*94.8%*	
			*Unknown*	*1*	*2.0000 [1.1641; 2.8359]*	*--*	
		*Study’s design*	*imaging*	*7*	*1.9514 [1.6853; 2.2175]*	*23.6%*	*0.4883*
			*osteological*	*10*	*1.6417 [0.8074; 2.4761]*	*96.3%*	
8	FM Width: Males vs. Females 2.0167 [1.3484; 2.6850] *p*-value < 0.0001 I^2^ = 98.0% [97.4%; 98.4%] SSE: *p*-value = 0.6425 IOS: “Rai_2017”	Continent of origin	Africa	3	1.7473 [0.9057; 2.5889]	52.4%	< 0.0001
			Asia	6	2.7994 [1.2816; 4.3172]	97.6%	
			Europe	3	1.5824 [1.2711; 1.8937]	33.0%	
			America	3	0.9022 [0.8415; 0.9629]	0.0%	
			Unknown	1	2.2000 [1.2143; 3.1857]	--	
		Study’s design	imaging	8	2.3961 [1.4066; 3.3856]	97.7%	0.2519
			osteological	8	1.6294 [0.7685; 2.4903]	94.5%	
	Re-estimation after excluding the IOS: “Rai_2017”						
	*FM Width: Males vs. Females* *1.7486 [1.2524; 2.2447]* *p-value < 0.0001* *I^2^ = 92.2% [88.8%; 94.6%]* *SSE: p-value = 0.0956*	*Continent* *of origin*	*Africa*	*3*	*1.7473 [0.9057; 2.5889]*	*52.4%*	*<0.0001*
			*Asia*	*5*	*2.2536 [0.9137; 3.5934]*	*92.0%*	
			*Europe*	*3*	*1.5824 [1.2711; 1.8937]*	*33.0%*	
			*America*	*3*	*0.9022 [0.8415; 0.9629]*	*0.0%*	
			*Unknown*	*1*	*2.2000 [1.2143; 3.1857]*	*--*	
		*Study’s design*	*imaging*	*7*	*1.8686 [1.3577; 2.3794]*	*75.6%*	*0.6396*
			*osteological*	*8*	*1.6294 [0.7685; 2.4903]*	*94.5%*	
9	OC Length (Left): Males vs. Females 1.9085 [1.4429; 2.3742] *p*-value < 0.0001 I^2^ = 97.3% [96.6%; 97.8%] SSE: *p*-value = 0.1582 IOS: none	Continent of origin	Africa	4	2.7115 [1.5484; 3.8746]	95.3%	0.1599
			Asia	10	1.7444 [0.9768; 2.5120]	98.2%	
			America	3	2.0307 [1.0915; 2.9699]	97.0%	
			Europe	4	1.4413 [1.0823; 1.8002]	52.5%	
		Study’s design	imaging	11	2.2590 [1.5636; 2.9544]	98.3%	0.0913
			osteological	10	1.5114 [0.9923; 2.0305]	82.1%	
10	OC Length (Right): Males vs. Females 2.0960 [1.5687; 2.6232] *p*-value < 0.0001 I^2^ = 98.1% [97.7%; 98.5%] SSE: *p*-value = 0.1357 IOS: “Rai_2017”	Continent of origin	Africa	4	2.7650 [1.6842; 3.8457]	95.9%	0.0585
			Asia	10	2.1949 [1.2589; 3.1308]	98.8%	
			America	3	1.7341 [0.6807; 2.7876]	97.3%	
			Europe	4	1.4283 [1.1458; 1.7108]	0.0%	
		**Study’s design**	**imaging**	**11**	**2.5735 [1.6979; 3.4491]**	**98.9%**	**0.0268**
			**osteological**	**10**	**1.5398 [1.2746; 1.8051]**	**31.5%**	
	Re-estimation after excluding the IOS: “Rai_2017”						
	*OC Length (Right): Males vs. Females* *1.8948 [1.5266; 2.2630]* *p-value < 0.0001* *I^2^ = 94.0% [92.1%; 95.5%]* *SSE: p-value = 0.5164*	*Continent* *of origin*	*Africa*	*4*	*2.7650 [1.6842; 3.8457]*	*95.9%*	*0.0935*
			*Asia*	*9*	*1.7422 [1.3343; 2.1501]*	*77.7%*	
			*America*	*3*	*1.7341 [0.6807; 2.7876]*	*97.3%*	
			*Europe*	*4*	*1.4283 [1.1458; 1.7108]*	*0.0%*	
		***Study’s design***	***imaging***	***10***	***2.2191 [1.6087; 2.8296]***	***96.8%***	***0.0454***
			**osteological**	***10***	***1.5398 [1.2746; 1.8051]***	***31.5%***	
11	OC Width (Left): Males vs. Females 0.6660 [0.1992; 1.1328] *p*-value = 0.0052 I^2^ = 97.1% [96.2%; 97.7%] SSE: *p*-value = 0.0566 IOS: “Rai_2017”	Continent of origin	Africa	4	0.7610 [ 0.4237; 1.0983]	75.4%	0.2270
			Asia	8	0.4979 [−0.5777; 1.5735]	98.7%	
			America	3	0.9961 [ 0.6507; 1.3415]	88.3%	
			Europe	3	0.5765 [ 0.3607; 0.7923]	0.0%	
		Study’s design	imaging	10	0.9990 [ 0.3050; 1.6930]	98.2%	0.0819
			osteological	8	0.2732 [−0.1593; 0.7058]	83.4%	
	Re-estimation after excluding the IOS: “Rai_2017”						
	*OC Width (Left): Males vs. Females* *0.5054 [0.2685; 0.7423]* *p-value < 0.0001* *I^2^ = 89.1% [84.2%; 92.5%]* *SSE: p-value = 0.0133*	*Continent* *of origin*	*Africa*	*4*	*0.7610 [ 0.4237; 1.0983]*	*75.4%*	*0.0027*
			*Asia*	*7*	*0.0793 [−0.2769; 0.4354]*	*76.4%*	
			*America*	*3*	*0.9961 [ 0.6507; 1.3415]*	*88.3%*	
			*Europe*	*3*	*0.5765 [ 0.3607; 0.7923]*	*0.0%*	
		*Study’s design*	*imaging*	*9*	*0.6748 [ 0.4156; 0.9340]*	*90.8%*	*0.1186*
			osteological	*8*	*0.2732 [−0.1593; 0.7058]*	*83.4%*	
12	OC Width (Right): Males vs. Females 0.6800 [0.1887; 1.1714] *p*-value = 0.0067 I^2^ = 98.9% [98.7%; 99.1%] SSE: *p*-value = 0.0050 IOS: “Rai_2017”	Continent of origin	Africa	3	0.7238 [ 0.3357; 1.1120]	76.6%	0.1596
			Asia	8	0.6190 [−0.4468; 1.6849]	99.4%	
			America	3	0.9595 [ 0.6905; 1.2286]	77.7%	
			Europe	3	0.4889 [ 0.1814; 0.7964]	52.5%	
		**Study’s design**	**imaging**	**9**	**1.1159 [ 0.4003; 1.8314]**	**99.4%**	**0.0353**
			**osteological**	**8**	**0.2019 [−0.2589; 0.6627]**	**83.0%**	
	Re-estimation after excluding the IOS: “Rai_2017”						
	*OC Width (Right): Males vs. Females* *0.5107 [0.2560; 0.7653]* *p-value < 0.0001* *I^2^ = 87.5% [81.3%; 91.6%]* *SSE: p-value = 0.0045*	*Continent* *of origin*	*Africa*	*3*	*0.7238 [ 0.3357; 1.1120]*	*76.6%*	*0.0296*
			*Asia*	*7*	*0.1813 [−0.3557; 0.7184]*	*85.2%*	
			*America*	*3*	*0.9595 [ 0.6905; 1.2286]*	*77.7%*	
			*Europe*	*3*	*0.4889 [ 0.1814; 0.7964]*	*52.5%*	
		***Study’s design***	***imaging***	***8***	***0.7573 [ 0.5347; 0.9799]***	***88.1%***	***0.0334***
			**osteological**	***8***	***0.2019 [−0.2589; 0.6627]***	***83.0%***	
13	OC Thickness (Left): Males vs. Females 0.6261 [0.3134; 0.9388] *p*-value < 0.0001 I^2^ = 73.4% [39.0%; 88.4%] SSE: k* = 6 < 10 (k.min = 10) IOS: none	Continent of origin	Africa	1	0.9700 [0.4859; 1.4541]	--	0.3747
			Asia	4	0.5729 [0.1016; 1.0442]	79.9%	
			America	1	0.6000 [0.3706; 0.8294]	--	
		Study’s design	imaging	3	0.6759 [ 0.4966; 0.8553]	0.0%	0.7118
			osteological	3	0.5442 [−0.1311; 1.2196]	84.2%	
14	OC Thickness (Right): Males vs. Females 0.3680 [0.1856; 0.5505] *p*-value < 0.0001 I^2^ = 32.9% [0.0%; 72.9%] SSE: k* = 6 < 10 (k.min = 10) IOS: none	Continent of origin	Africa	1	0.6100 [0.1469; 1.0731]	--	0.5984
			Asia	4	0.3275 [0.0367; 0.6184]	45.3%	
			America	1	0.4000 [0.1642; 0.6358]	--	
		Study’s design	imaging	3	0.4674 [ 0.2741; 0.6606]	0.0%	0.0796
			osteological	3	0.2031 [−0.0205; 0.4266]	41.5%	

k, Number of studies combined; 95%-CI, 95% confidence interval; I^2^, Higgins I^2^ statistic; SSE, Small-Study Effect (test of funnel plot asymmetry); IOS, Influential outlier study; bold font indicates the statistically significant results of subgroup analyses with at least four studies per subgroup; italic font indicates the results of re-estimation after excluding the Influential outlier studies; k*, Number of studies (k < 10) too small to test for small-study effects (k.min = 10).

## Supplementary Materials

The following supporting information can be downloaded at: https://www.mdpi.com/article/10.3390/diagnostics15111359/s1, Meta-analytic plots of pooled morphometric means (Figures S1–S11) and pooled morphometric mean differences (Figures S12–S21): Forest plots evaluating the means, Forest plots of Subgroup analyses based on nationality, Forest plots of Subgroup analyses based on study’s type, Funnel plots for the assessment of small-study effect, Influence analyses (Influence Diagnostics), Outlier analyses (Identified outliers and Forest plots with outliers removed).

## Abbreviations

FM	Foramen Magnum
OC	Occipital Condyle
OCL	Occipital Condyle Length
OCW	Occipital Condyle Width
OCT	Occipital Condyle Thickness
FML	Foramen Magnum Length
FMW	Foramen Magnum Width
SD	Sagittal Diameter
TD	Transverse Diameter
APD	Anteroposterior Diameter
ROC	Receiver Operating Characteristic
AQUA	Anatomical Quality Assurance Tool
PRISMA	Preferred Reporting Items for Systematic Reviews and Meta-Analyses
IOS	Influential Outlier Study
MD	Mean Difference
CI	Confidence Interval
SSE	Small Study Effect
I^2^	Higgins’ I-squared Statistic
CART	Classification and Regression Trees
meta-CART	Meta-analysis with Classification and Regression Trees
