# Supplementary material for: Morphometric Assessment of Occipital Condyles and Foramen Magnum Reveals Enhanced Sexual Dimorphism Detection via 3D Imaging: A Systematic Review and Meta-Analysis Utilizing Classification and Regression Trees
Source: Diagnostics (Basel). 2025 May 28;15(11):1359. doi: 10.3390/diagnostics15111359 (PMC12155437; doi:10.3390/diagnostics15111359)
Supplement: Supplementary file 1 [file diagnostics-15-01359-s001.zip › diagnostics-3632235-supplementary.pdf]

Appendix: Supplemental Figure 1: OC Length

Forest plot evaluating the mean length

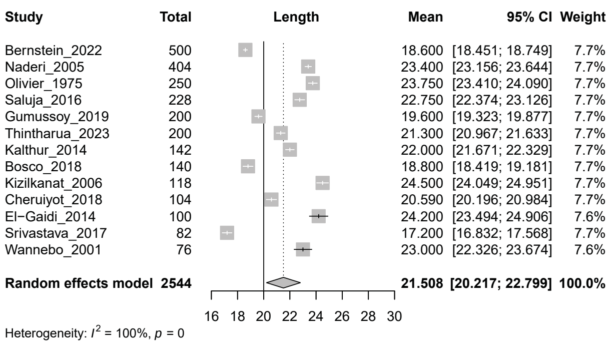

Funnel plot for the assessment of small-study effect

(Test of funnel plot asymmetry: p-value = 0.1081)

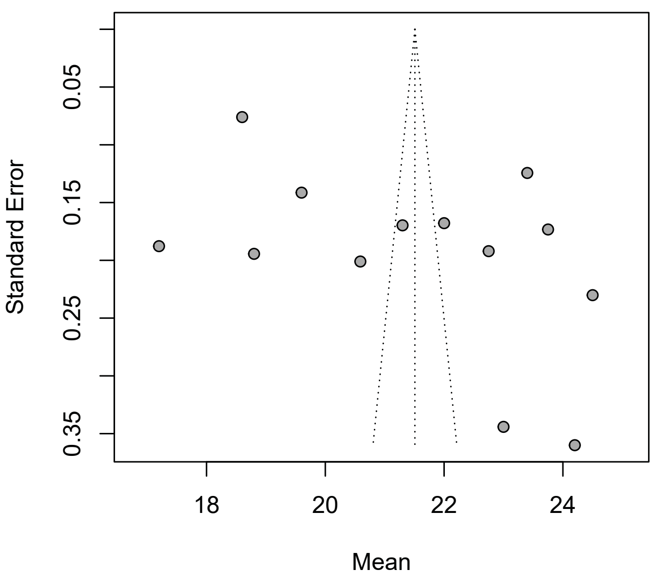

Forest plot of Subgroup analysis based on nationality

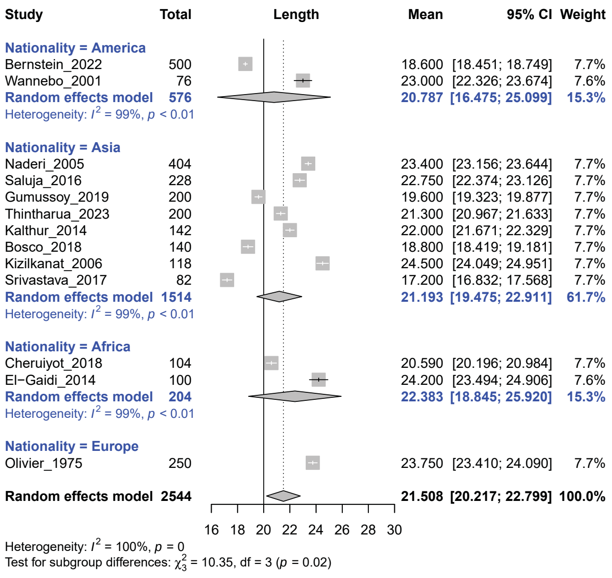

Influence analysis: Influence Diagnostics

(Identified influential studies: none)

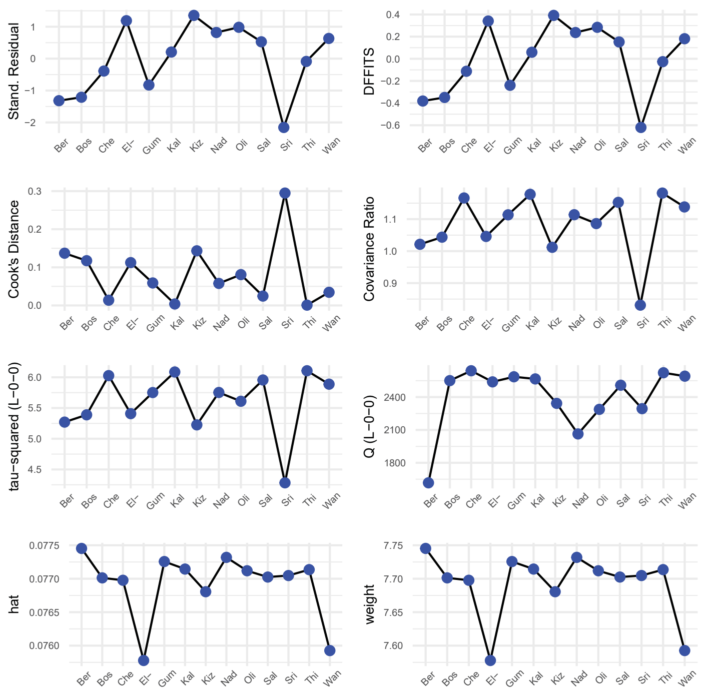

Forest plot of Subgroup analysis based on study's type

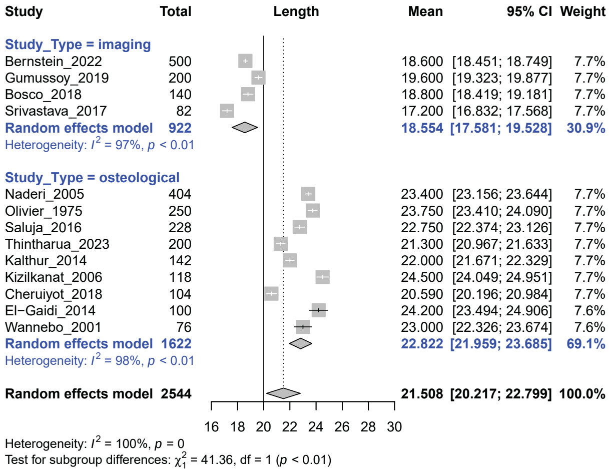

Outlier analysis: Identified outliers

Outlier studies:

"Bernstein_2022", "Bosco_2018", "El-Gaidi_2014", "Gumussoy_2019", "Kizilkanat_2006", "Naderi_2005", "Olivier_1975", "Srivastava_2017"

Forest plot with outliers removed

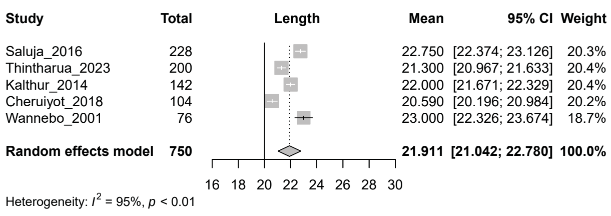

Appendix: Supplemental Figure 2: OC Width

Forest plot evaluating the mean width

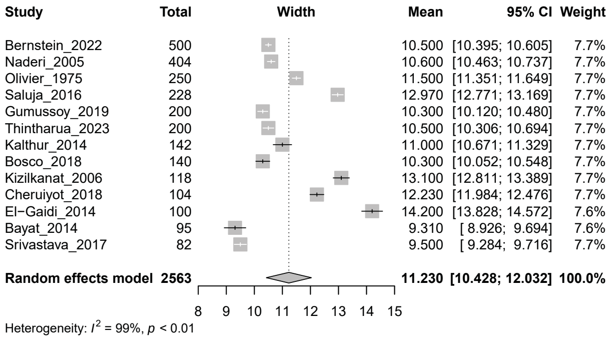

Funnel plot for the assessment of small-study effect

(Test of funnel plot asymmetry: p-value = 0.1081)

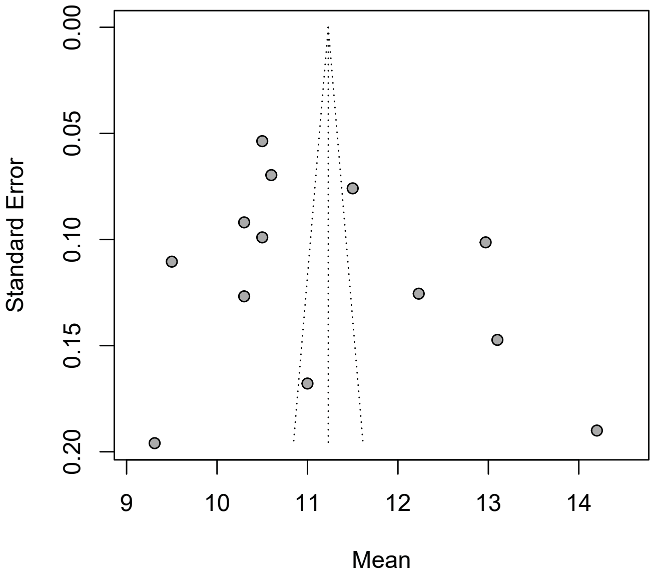

Forest plot of Subgroup analysis based on nationality

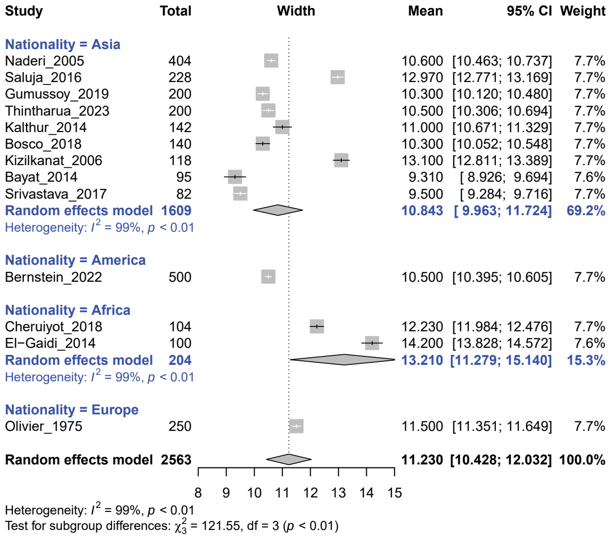

Influence analysis: Influence Diagnostics

(Identified influential studies: none)

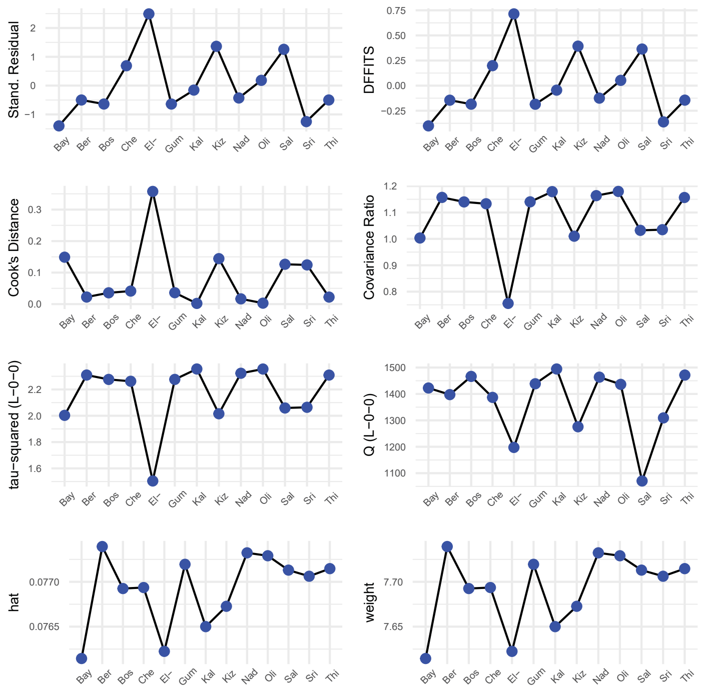

Forest plot of Subgroup analysis based on study's type

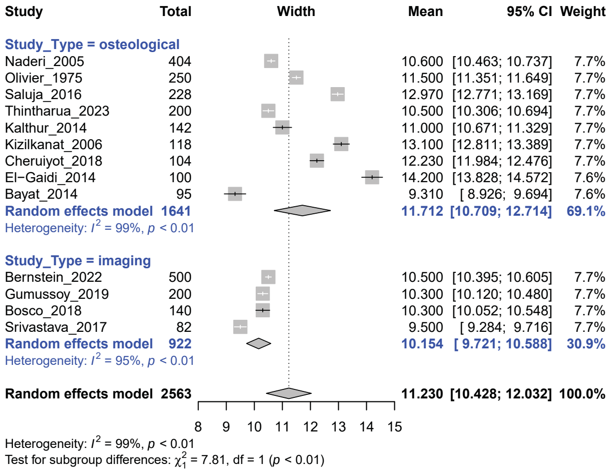

Outlier analysis: Identified outliers

Outlier studies:

"Bayat_2014", "El-Gaidi_2014", "Kizilkanat_2006", "Saluja_2016", "Srivastava_2017"

Forest plot with outliers removed

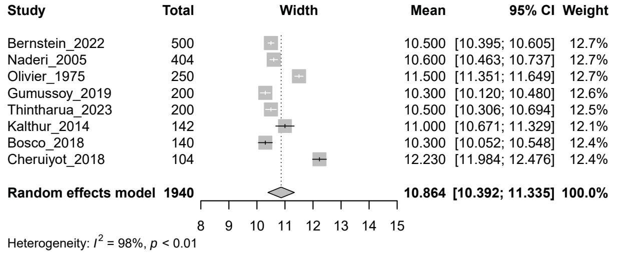

Appendix: Supplemental Figure 3: OC Thickness

Forest plot evaluating the mean thickness

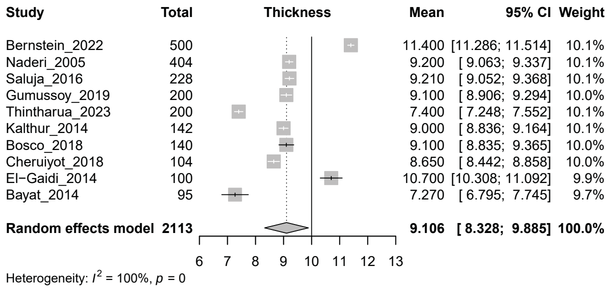

Funnel plot for the assessment of small-study effect

(Test of funnel plot asymmetry: p-value = 0.5070)

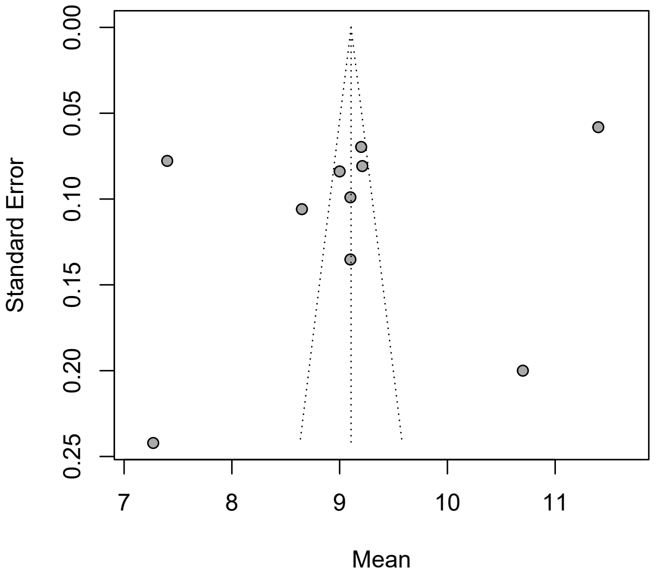

Forest plot of Subgroup analysis based on nationality

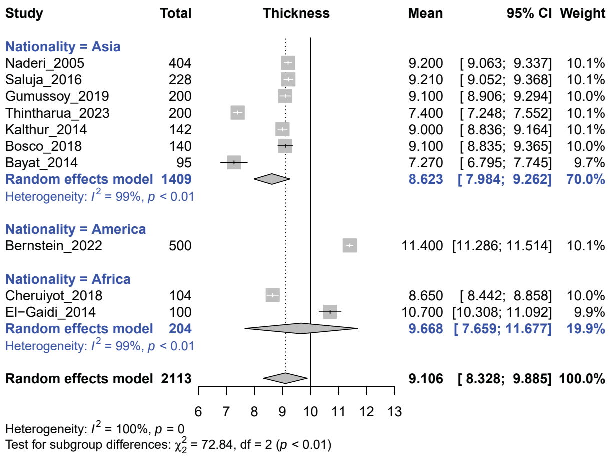

Influence analysis: Influence Diagnostics

(Identified influential studies: none)

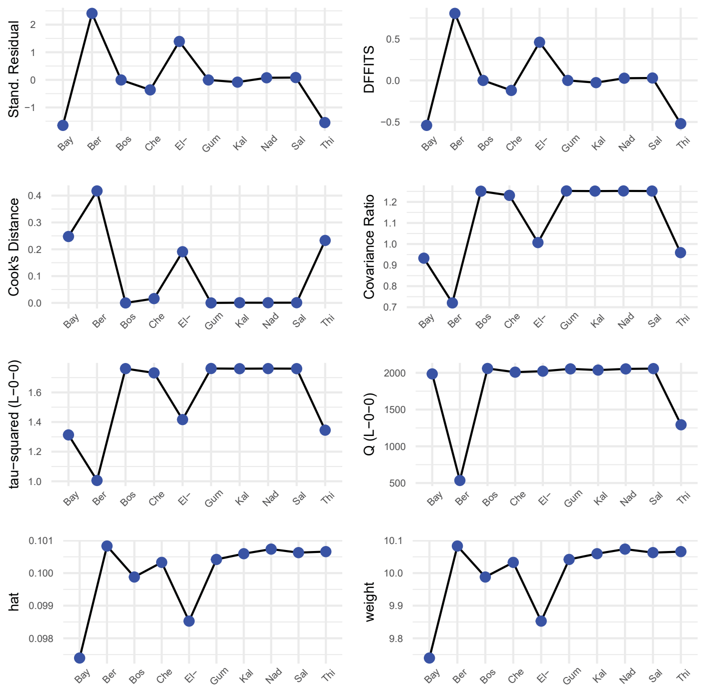

Forest plot of Subgroup analysis based on study's type

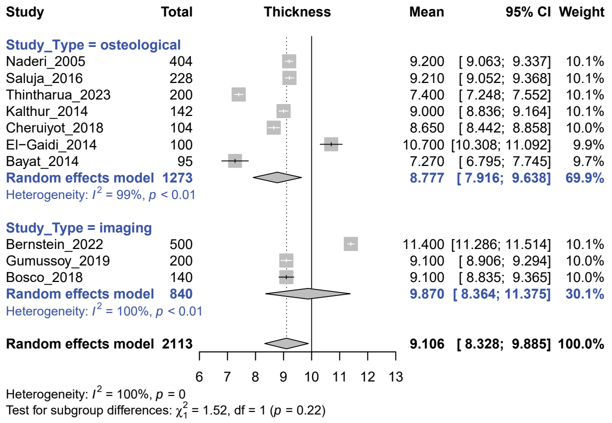

Outlier analysis: Identified outliers

Outlier studies:

"Bayat_2014", "Bernstein_2022", "El-Gaidi_2014", "Thintharua_2023"

Forest plot with outliers removed

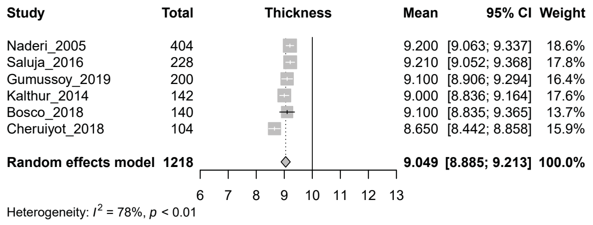

Appendix: Supplemental Figure 4: FM Length

Forest plot evaluating the mean length

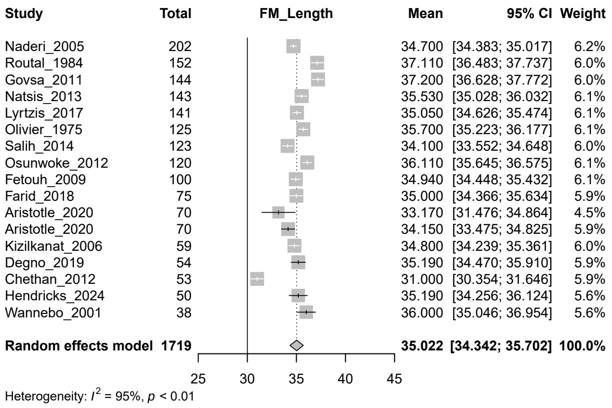

Funnel plot for the assessment of small-study effect

(Test of funnel plot asymmetry: p-value = 0.3637)

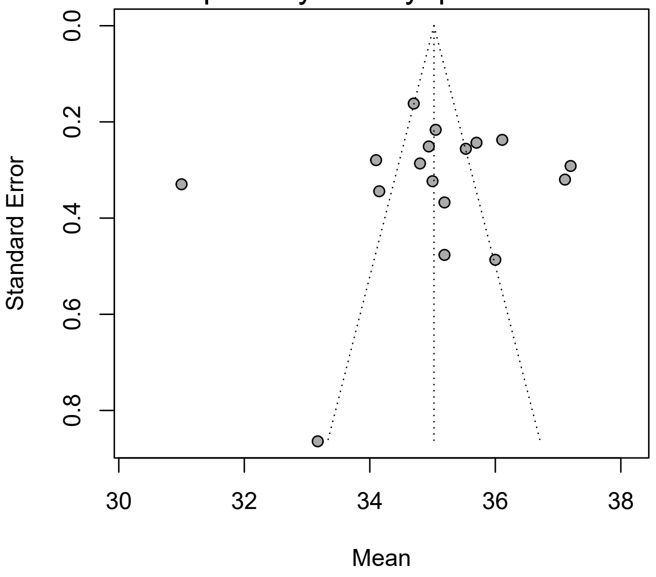

Forest plot of Subgroup analysis based on nationality

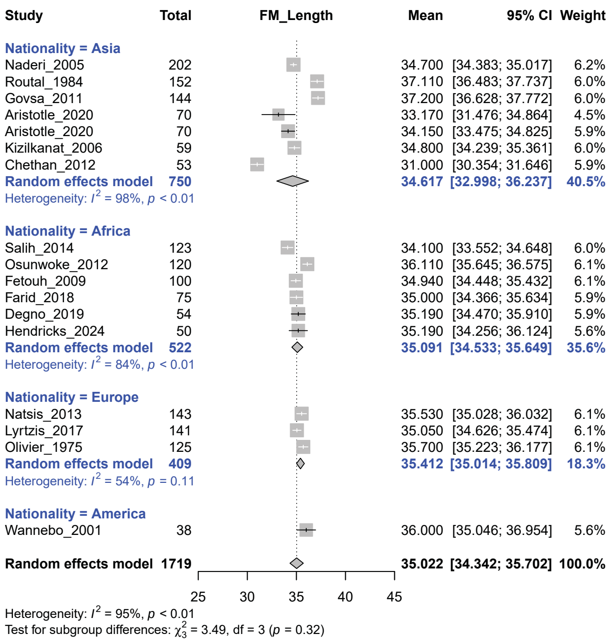

Influence analysis: Influence Diagnostics

(Identified influential studies: "Chethan_2012")

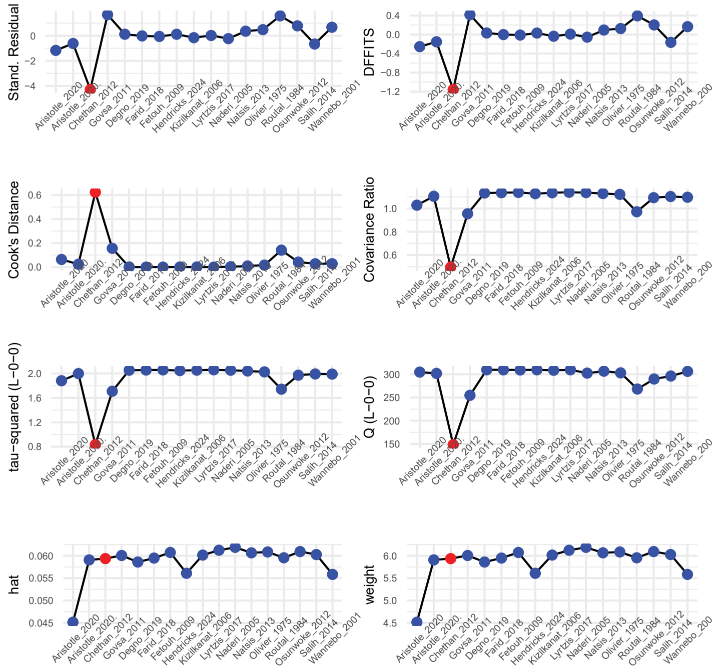

Forest plot of Subgroup analysis based on study's type

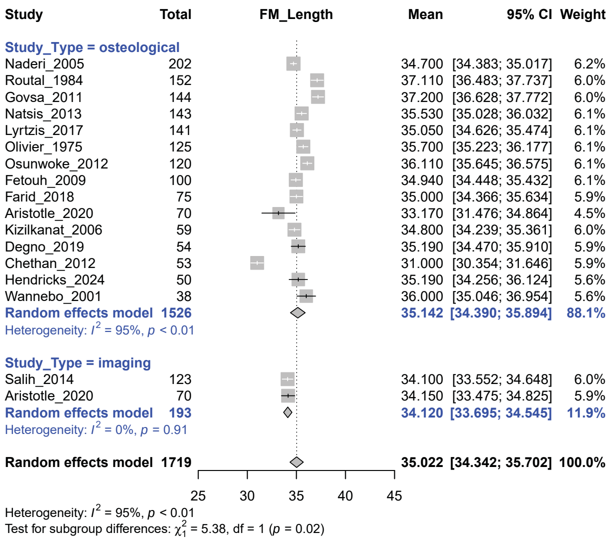

Outlier analysis: Identified outliers

Outlier studies:

"Chethan_2012", "Govsa_2011", "Routal_1984"

Forest plot with outliers removed

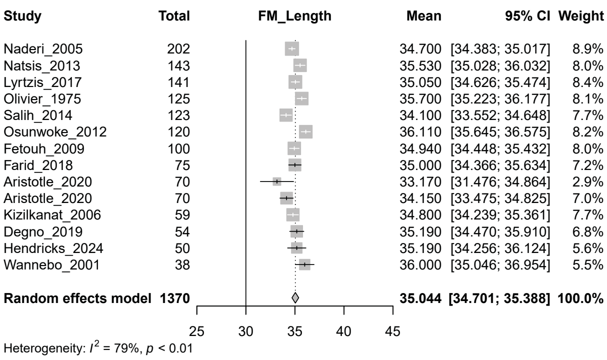

Appendix: Supplemental Figure 5: FM Width

Forest plot evaluating the mean width

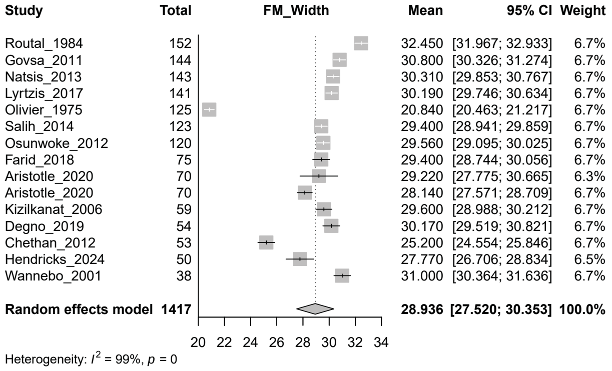

Funnel plot for the assessment of small-study effect

(Test of funnel plot asymmetry: p-value = 0.9082)

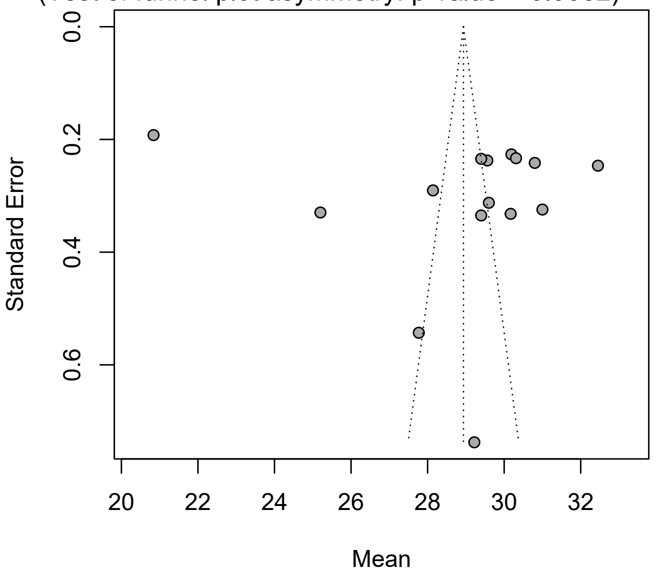

Forest plot of Subgroup analysis based on nationality

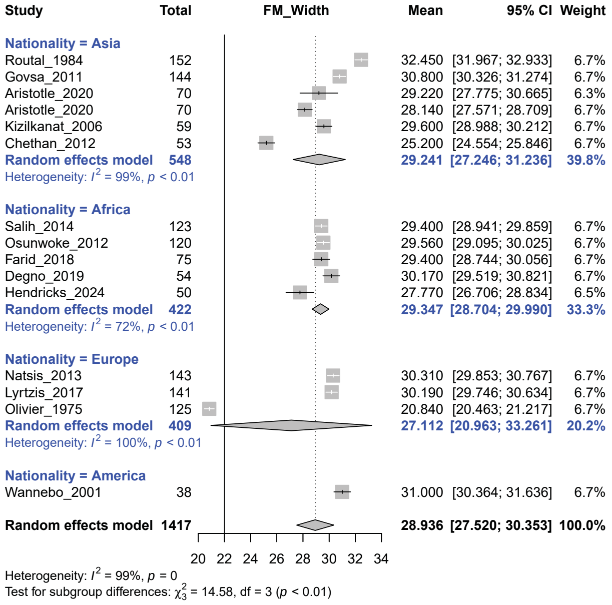

Influence analysis: Influence Diagnostics

(Identified influential studies: "Olivier_1975")

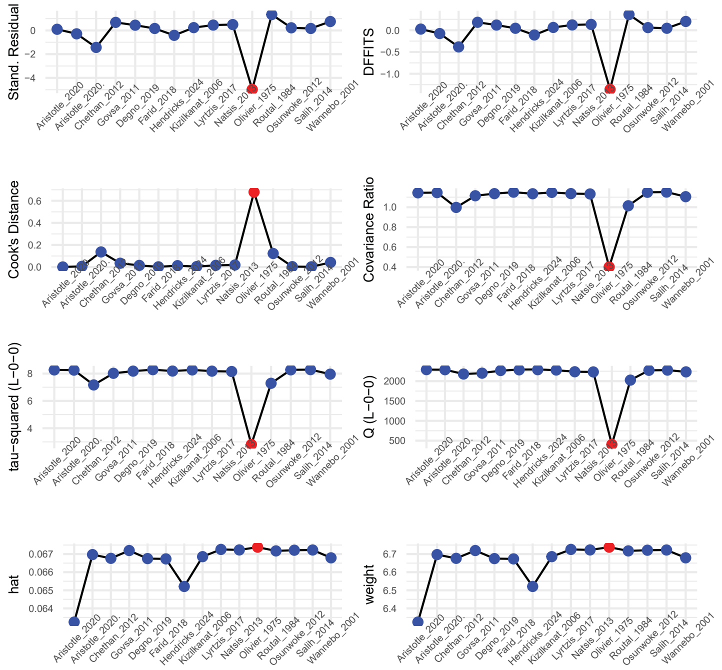

Forest plot of Subgroup analysis based on study's type

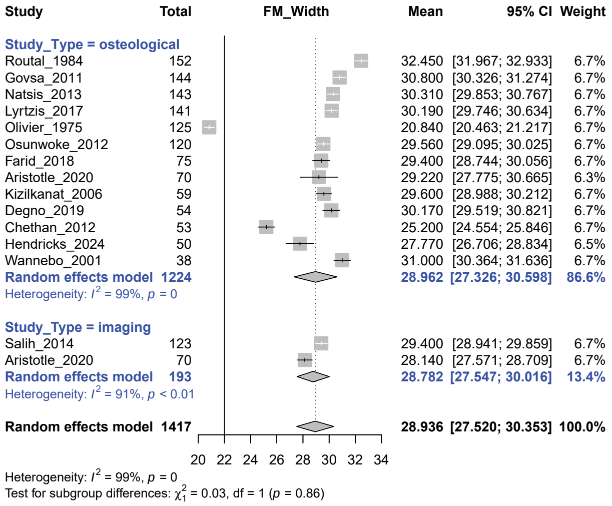

Outlier analysis: Identified outliers

Outlier studies:
"Chethan_2012", "Olivier_1975", "Routal_1984", "Wannebo_2001"

Forest plot with outliers removed

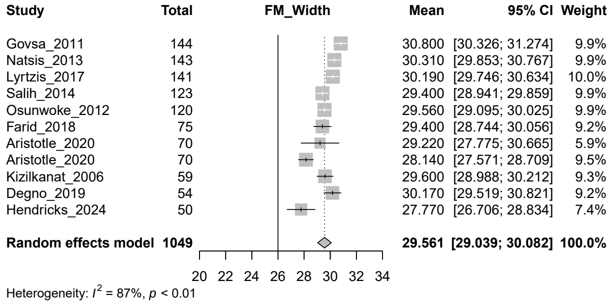

Appendix: Supplemental Figure 6: OC Length (Left)

Forest plot evaluating the mean length

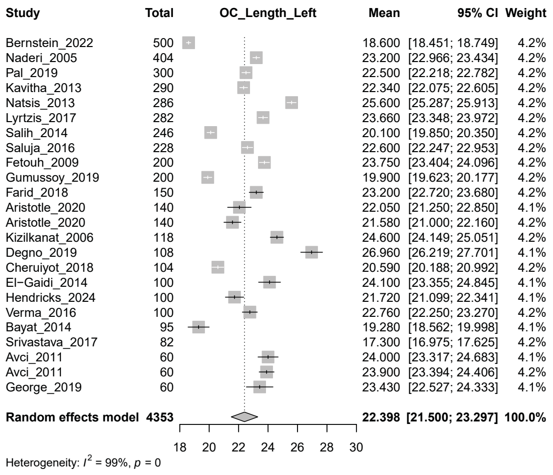

Funnel plot for the assessment of small-study effect

(Test of funnel plot asymmetry: p-value = 0.1321)

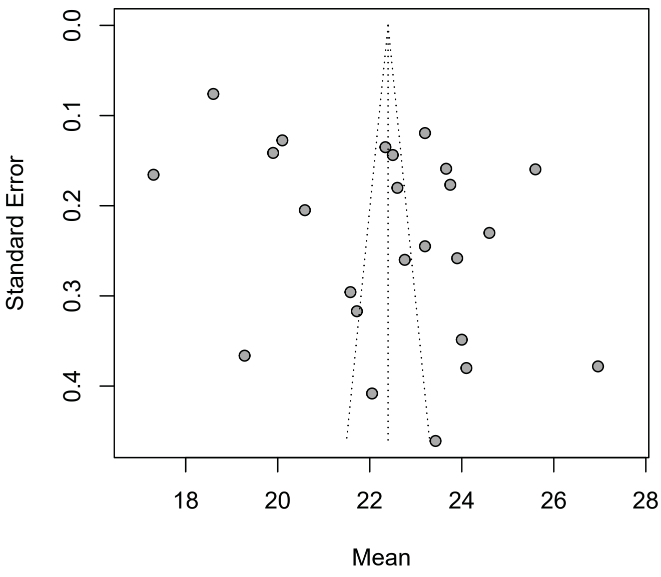

Forest plot of Subgroup analysis based on nationality

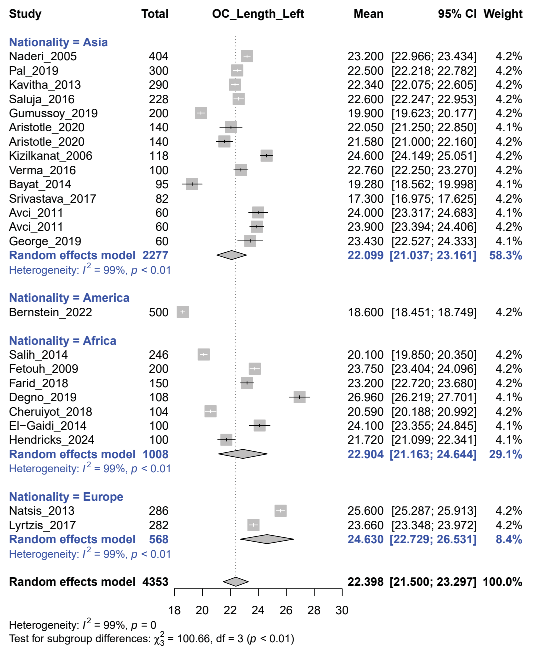

Influence analysis: Influence Diagnostics

(Identified influential studies: none)

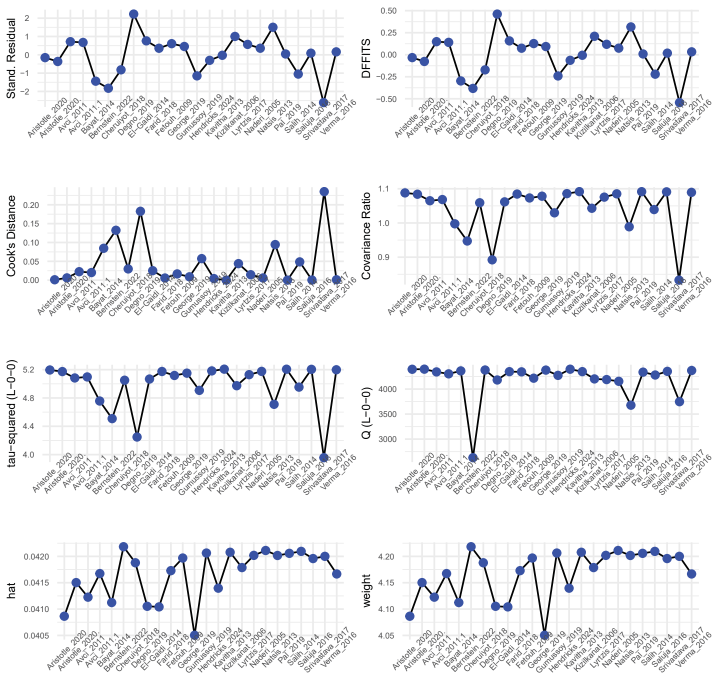

Forest plot of Subgroup analysis based on study's type

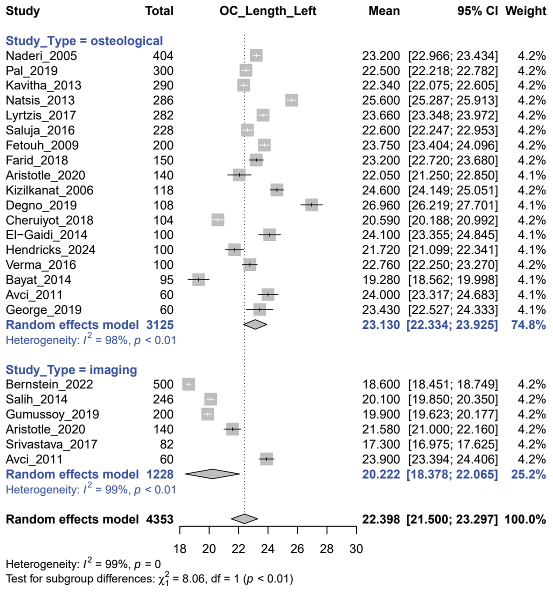

Outlier analysis: Identified outliers

Outlier studies:

"Avci_2011", "Avci_2011", "Bayat_2014", "Bernstein_2022", "Cheruiyot_2018", "Degno_2019", "El-Gaidi_2014", "Fetouh_2009", "Gumussoy_2019", "Kizilkanat_2006", "Lyrtzis_2017", "Natsis_2013", "Salih_2014", "Srivastava_2017"

Forest plot with outliers removed

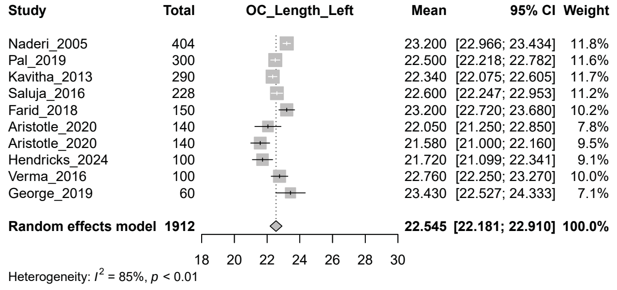

Appendix: Supplemental Figure 7: OC Length (Right)

Forest plot evaluating the mean length

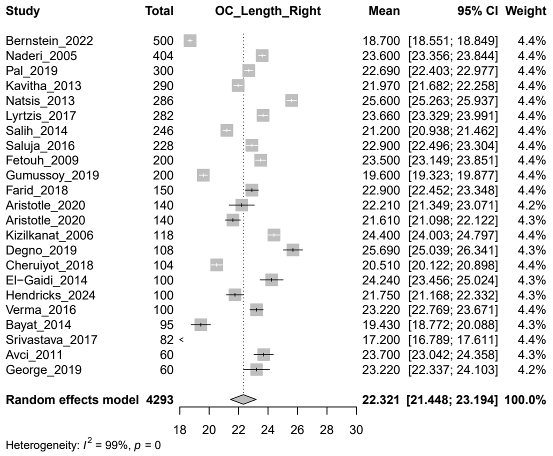

Funnel plot for the assessment of small-study effect

(Test of funnel plot asymmetry: p-value = 0.2777)

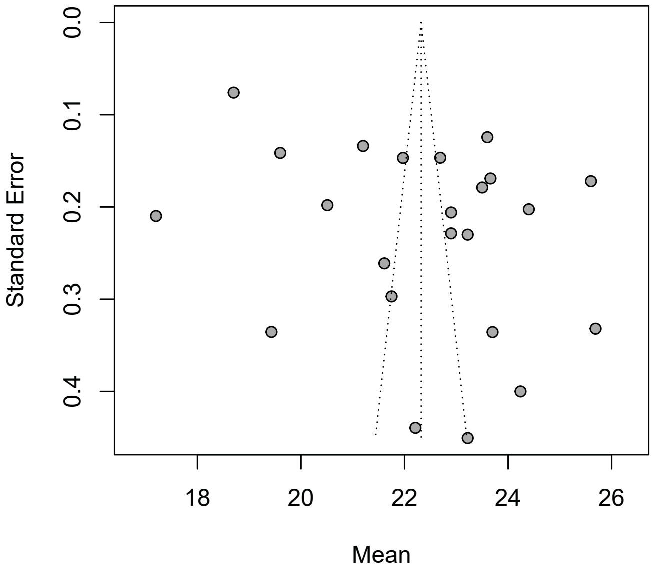

Forest plot of Subgroup analysis based on nationality

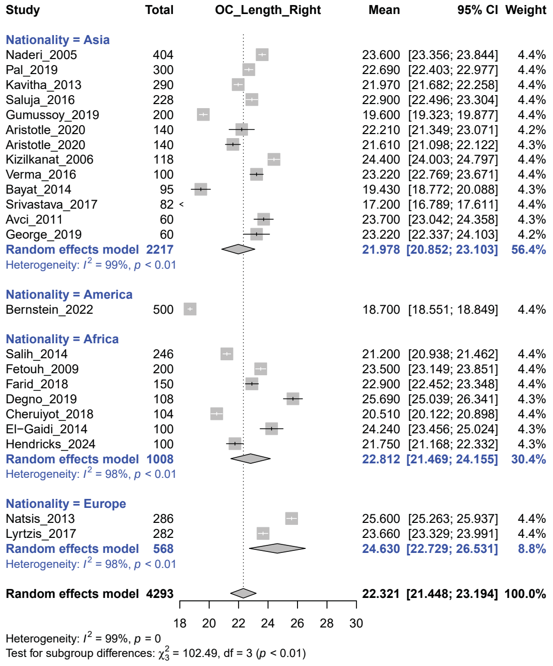

Influence analysis: Influence Diagnostics

(Identified influential studies: none)

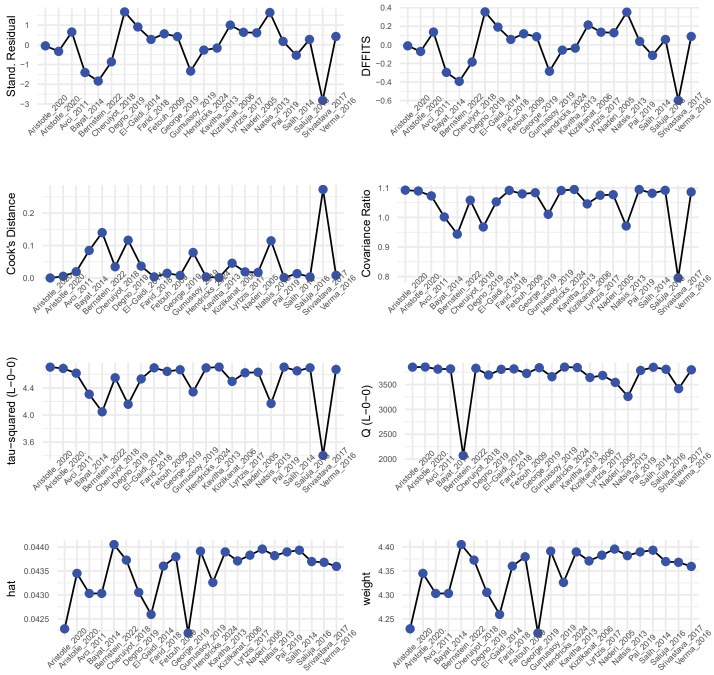

Forest plot of Subgroup analysis based on study's type

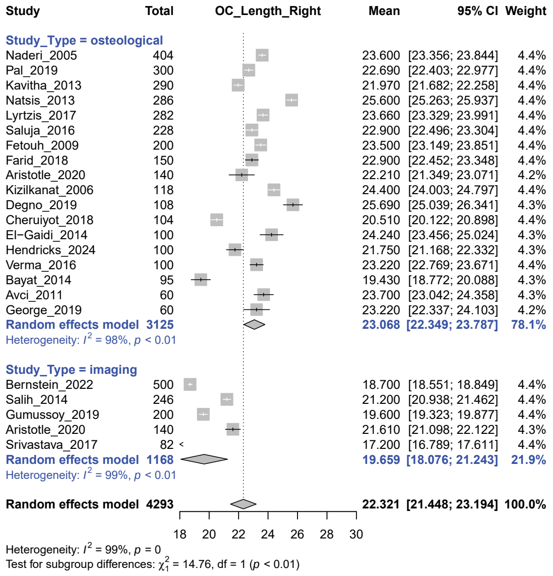

Outlier analysis: Identified outliers

Outlier studies:

"Bayat_2014", "Bernstein_2022", "Cheruiyot_2018", "Degno_2019", "El-Gaidi_2014", "Gumussoy_2019", "Kizilkanat_2006", "Lyrtzis_2017", "Naderi_2005", "Natsis_2013", "Srivastava_2017"

Forest plot with outliers removed

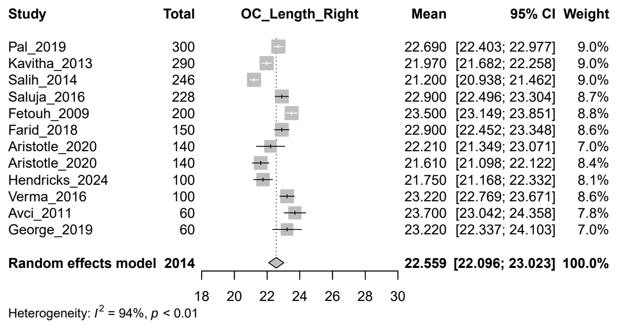

Appendix: Supplemental Figure 8: OC Width (Left)

Forest plot evaluating the mean width

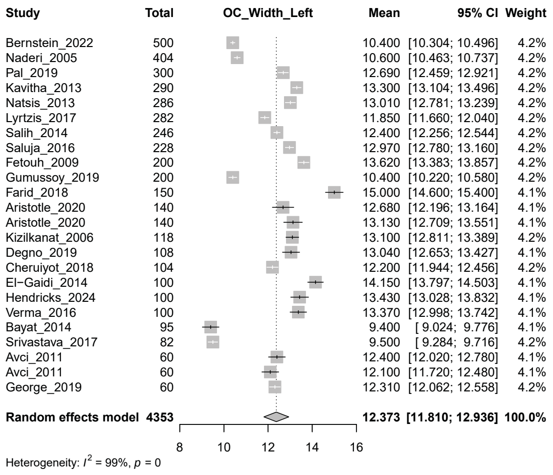

Funnel plot for the assessment of small-study effect

(Test of funnel plot asymmetry: p-value = 0.0467)

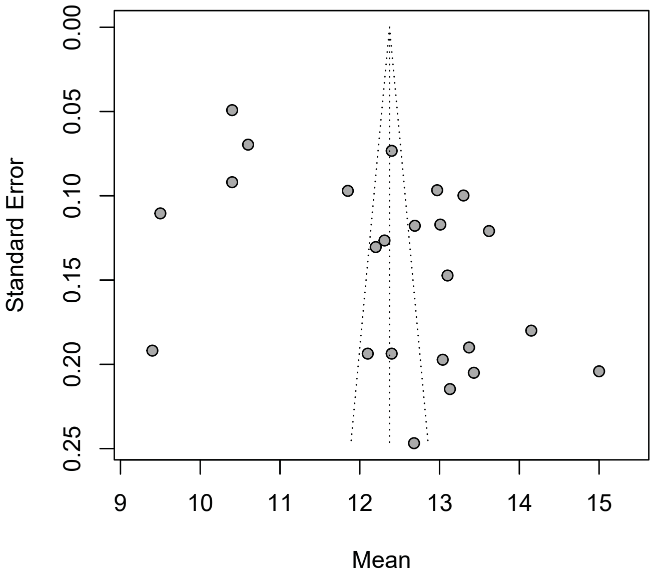

Forest plot of Subgroup analysis based on nationality

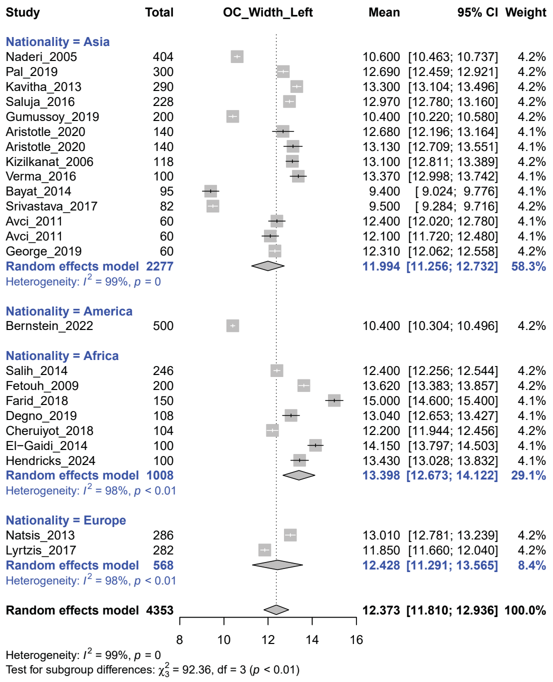

Influence analysis: Influence Diagnostics

(Identified influential studies: none)

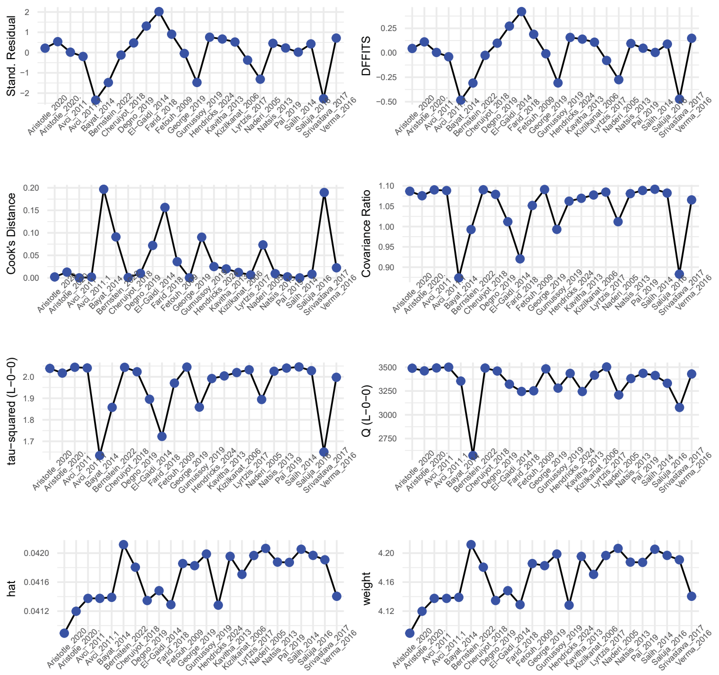

Forest plot of Subgroup analysis based on study's type

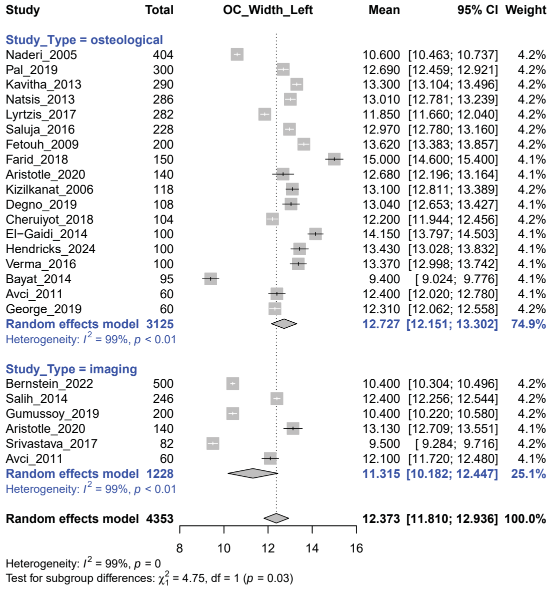

Outlier analysis: Identified outliers

Outlier studies:

"Bayat_2014", "Bernstein_2022", "El-Gaidi_2014", "Farid_2018", "Fetouh_2009", "Gumussoy_2019", "Hendricks_2024", "Kavitha_2013", "Naderi_2005", "Srivastava_2017", "Verma_2016"

Forest plot with outliers removed

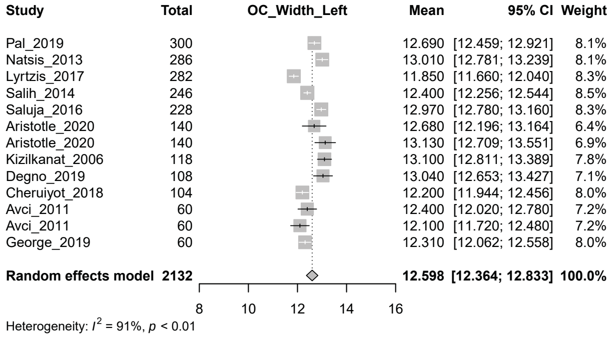

Appendix: Supplemental Figure 9: OC Width (Right)

Forest plot evaluating the mean width

Study	Total	OC_Width_Right	Mean	95% CI	Weight
Bernstein_2022	500		10.500	[10.395; 10.605]	4.2%
Naderi_2005	404		10.600	[10.463; 10.737]	4.2%
Pal_2019	300		12.650	[12.426; 12.874]	4.2%
Kavitha_2013	290		13.050	[12.854; 13.246]	4.2%
Natsis_2013	286		13.090	[12.975; 13.205]	4.2%
Lyrtzis_2017	282		11.770	[11.593; 11.947]	4.2%
Salih_2014	246		13.100	[12.950; 13.250]	4.2%
Saluja_2016	228		12.980	[12.770; 13.190]	4.2%
Fetouh_2009	200		13.580	[13.328; 13.832]	4.2%
Gumussoy_2019	200		10.200	[10.034; 10.366]	4.2%
Farid_2018	150		14.800	[14.432; 15.168]	4.1%
Aristotle_2020	140		12.570	[12.156; 12.984]	4.1%
Aristotle_2020	140		13.040	[12.778; 13.302]	4.2%
Kizilkanat_2006	118		13.000	[12.729; 13.271]	4.2%
Degno_2019	108		12.760	[12.404; 13.116]	4.1%
Cheruiyot_2018	104		12.270	[12.030; 12.510]	4.2%
El−Gaidi_2014	100		14.150	[13.758; 14.542]	4.1%
Hendricks_2024	100		12.320	[11.924; 12.716]	4.1%
Verma_2016	100		12.930	[12.597; 13.263]	4.1%
Bayat_2014	95		9.210	[8.814; 9.606]	4.1%
Srivastava_2017	82		9.400	[9.162; 9.638]	4.2%
Avci_2011	60		12.200	[11.896; 12.504]	4.2%
Avci_2011	60		12.000	[11.671; 12.329]	4.1%
George_2019	60		12.390	[12.094; 12.686]	4.2%
**Random effects model**	**4353**		**12.271**	**[11.717; 12.826]**	**100.0%**

8 10 12 14 16

Heterogeneity: I^2 = 99%, p = 0

Funnel plot for the assessment of small-study effect

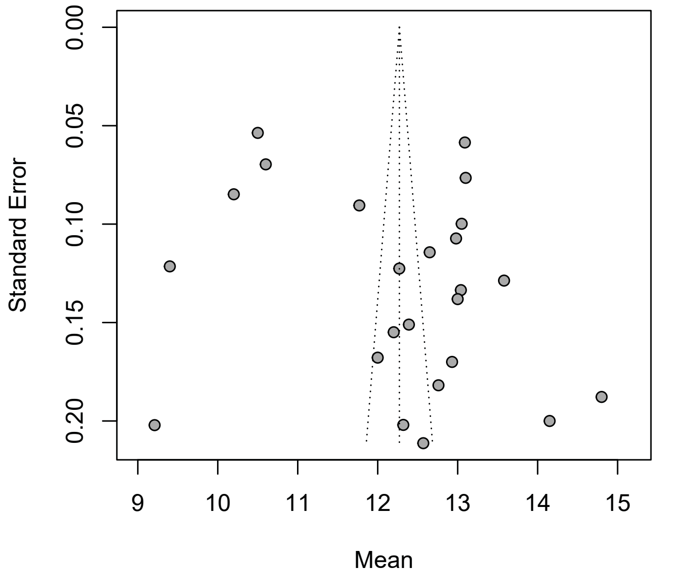

Forest plot of Subgroup analysis based on nationality

Study	Total	OC_Width_Right	Mean	95% CI	Weight
**Nationality = Asia**					
Naderi_2005	404		10.600	[10.463; 10.737]	4.2%
Pal_2019	300		12.650	[12.426; 12.874]	4.2%
Kavitha_2013	290		13.050	[12.854; 13.246]	4.2%
Saluja_2016	228		12.980	[12.770; 13.190]	4.2%
Gumussoy_2019	200		10.200	[10.034; 10.366]	4.2%
Aristotle_2020	140		12.570	[12.156; 12.984]	4.1%
Aristotle_2020	140		13.040	[12.778; 13.302]	4.2%
Kizilkanat_2006	118		13.000	[12.729; 13.271]	4.2%
Verma_2016	100		12.930	[12.597; 13.263]	4.1%
Bayat_2014	95		9.210	[8.814; 9.606]	4.1%
Srivastava_2017	82		9.400	[9.162; 9.638]	4.2%
Avci_2011	60		12.200	[11.896; 12.504]	4.2%
Avci_2011	60		12.000	[11.671; 12.329]	4.1%
George_2019	60		12.390	[12.094; 12.686]	4.2%
**Random effects model**	**2277**		**11.873**	**[11.140; 12.606]**	**58.3%**
Heterogeneity: I^2 = 99%, p = 0					
**Nationality = America**					
Bernstein_2022	500		10.500	[10.395; 10.605]	4.2%
**Nationality = Africa**					
Salih_2014	246		13.100	[12.950; 13.250]	4.2%
Fetouh_2009	200		13.580	[13.328; 13.832]	4.2%
Farid_2018	150		14.800	[14.432; 15.168]	4.1%
Degno_2019	108		12.760	[12.404; 13.116]	4.1%
Cheruiyot_2018	104		12.270	[12.030; 12.510]	4.2%
El−Gaidi_2014	100		14.150	[13.758; 14.542]	4.1%
Hendricks_2024	100		12.320	[11.924; 12.716]	4.1%
**Random effects model**	**1008**		**13.280**	**[12.581; 13.978]**	**29.1%**
Heterogeneity: I^2 = 97%, p < 0.01					
**Nationality = Europe**					
Natsis_2013	286		13.090	[12.975; 13.205]	4.2%
Lyrtzis_2017	282		11.770	[11.593; 11.947]	4.2%
**Random effects model**	**568**		**12.432**	**[11.138; 13.725]**	**8.4%**
Heterogeneity: I^2 = 99%, p < 0.01					
**Random effects model**	**4353**		**12.271**	**[11.717; 12.826]**	**100.0%**

8 10 12 14 16

Heterogeneity: I^2 = 99%, p = 0
Test for subgroup differences: χ^2_3 = 79.33, df = 3 (p < 0.01)

Influence analysis: Influence Diagnostics

(Identified influential studies: none)

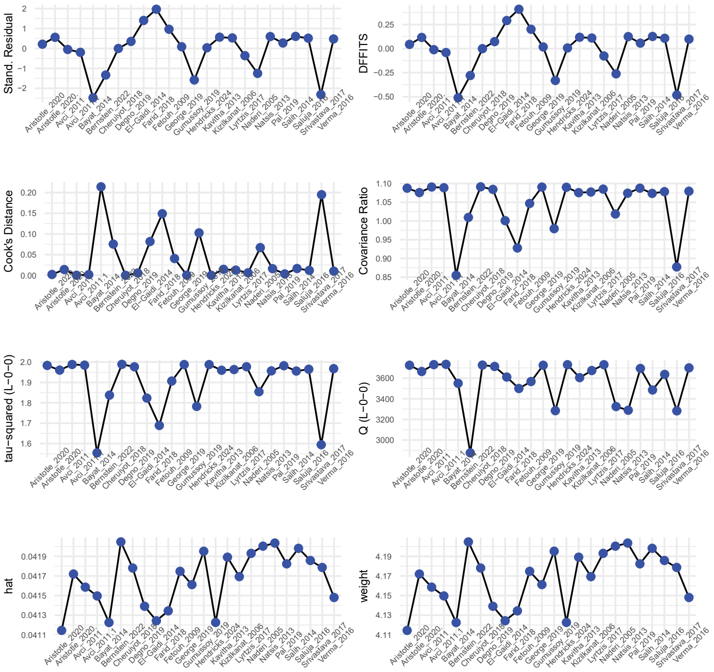

Forest plot of Subgroup analysis based on study's type

Study	Total	OC_Width_Right	Mean	95% CI	Weight
**Study_Type = osteological**					
Naderi_2005	404		10.600	[10.463; 10.737]	4.2%
Pal_2019	300		12.650	[12.426; 12.874]	4.2%
Kavitha_2013	290		13.050	[12.854; 13.246]	4.2%
Natsis_2013	286		13.090	[12.975; 13.205]	4.2%
Lyrtzis_2017	282		11.770	[11.593; 11.947]	4.2%
Saluja_2016	228		12.980	[12.770; 13.190]	4.2%
Fetouh_2009	200		13.580	[13.328; 13.832]	4.2%
Farid_2018	150		14.800	[14.432; 15.168]	4.1%
Aristotle_2020	140		12.570	[12.156; 12.984]	4.1%
Kizilkanat_2006	118		13.000	[12.729; 13.271]	4.2%
Degno_2019	108		12.760	[12.404; 13.116]	4.1%
Cheruiyot_2018	104		12.270	[12.030; 12.510]	4.2%
El−Gaidi_2014	100		14.150	[13.758; 14.542]	4.1%
Hendricks_2024	100		12.320	[11.924; 12.716]	4.1%
Verma_2016	100		12.930	[12.597; 13.263]	4.1%
Bayat_2014	95		9.210	[8.814; 9.606]	4.1%
Avci_2011	60		12.200	[11.896; 12.504]	4.2%
George_2019	60		12.390	[12.094; 12.686]	4.2%
**Random effects model**	**3125**		**12.573**	**[12.008; 13.137]**	**74.9%**
Heterogeneity: I^2 = 99%, p < 0.01					
**Study_Type = imaging**					
Bernstein_2022	500		10.500	[10.395; 10.605]	4.2%
Salih_2014	246		13.100	[12.950; 13.250]	4.2%
Gumussoy_2019	200		10.200	[10.034; 10.366]	4.2%
Aristotle_2020	140		13.040	[12.778; 13.302]	4.2%
Srivastava_2017	82		9.400	[9.162; 9.638]	4.2%
Avci_2011	60		12.000	[11.671; 12.329]	4.1%
**Random effects model**	**1228**		**11.372**	**[10.123; 12.622]**	**25.1%**
Heterogeneity: I^2 = 100%, p < 0.01					
**Random effects model**	**4353**		**12.271**	**[11.717; 12.826]**	**100.0%**

8 10 12 14 16

Heterogeneity: I^2 = 99%, p = 0
Test for subgroup differences: χ^2_1 = 2.95, df = 1 (p = 0.09)

Outlier analysis: Identified outliers

Outlier studies:

"Bayat_2014", "Bernstein_2022", "El-Gaidi_2014", "Farid_2018", "Fetouh_2009", "Gumussoy_2019", "Kavitha_2013", "Naderi_2005", "Natsis_2013", "Salih_2014", "Srivastava_2017"

Forest plot with outliers removed

Study	Total	OC_Width_Right	Mean	95% CI	Weight
Pal_2019	300		12.650	[12.426; 12.874]	8.1%
Lyrtzis_2017	282		11.770	[11.593; 11.947]	8.4%
Saluja_2016	228		12.980	[12.770; 13.190]	8.2%
Aristotle_2020	140		12.570	[12.156; 12.984]	6.9%
Aristotle_2020	140		13.040	[12.778; 13.302]	7.9%
Kizilkanat_2006	118		13.000	[12.729; 13.271]	7.9%
Degno_2019	108		12.760	[12.404; 13.116]	7.3%
Cheruiyot_2018	104		12.270	[12.030; 12.510]	8.1%
Hendricks_2024	100		12.320	[11.924; 12.716]	7.0%
Verma_2016	100		12.930	[12.597; 13.263]	7.4%
Avci_2011	60		12.200	[11.896; 12.504]	7.6%
Avci_2011	60		12.000	[11.671; 12.329]	7.5%
George_2019	60		12.390	[12.094; 12.686]	7.7%
**Random effects model**	**1800**		**12.528**	**[12.299; 12.758]**	**100.0%**

8 10 12 14 16

Heterogeneity: I^2 = 92%, p < 0.01

Appendix: Supplemental Figure 10: OC Thickness (Left)

Forest plot evaluating the mean thickness

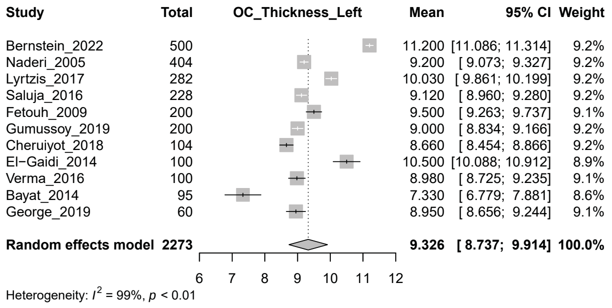

Funnel plot for the assessment of small-study effect

(Test of funnel plot asymmetry: p-value = 0.1359)

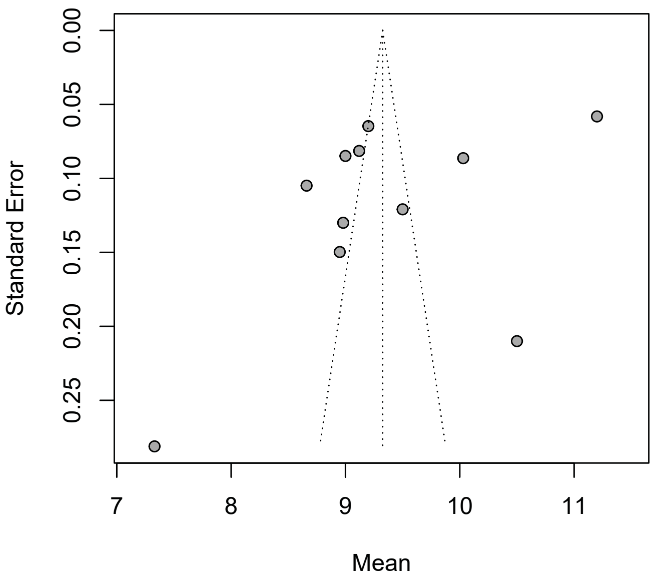

Forest plot of Subgroup analysis based on nationality

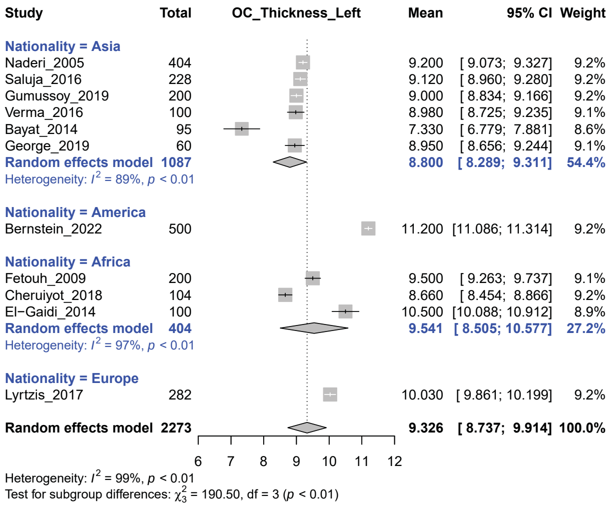

Influence analysis: Influence Diagnostics

(Identified influential studies: none)

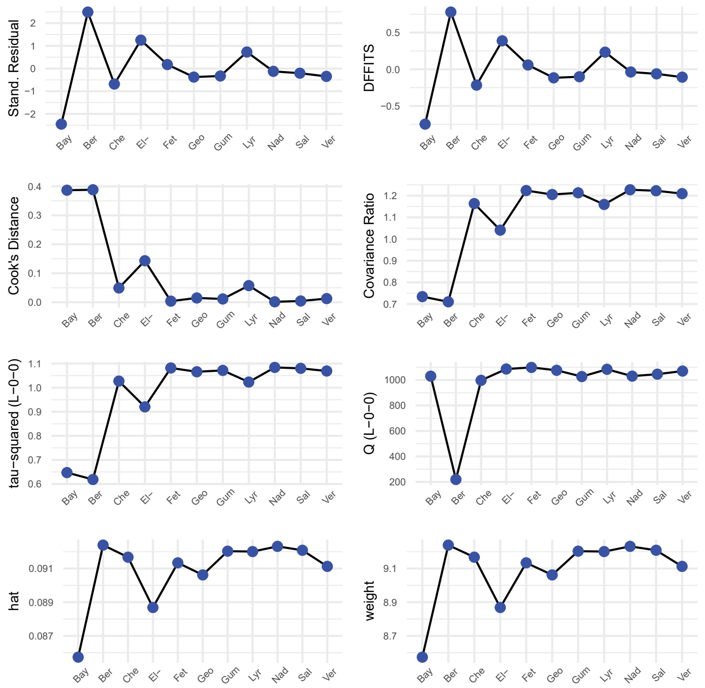

Forest plot of Subgroup analysis based on study's type

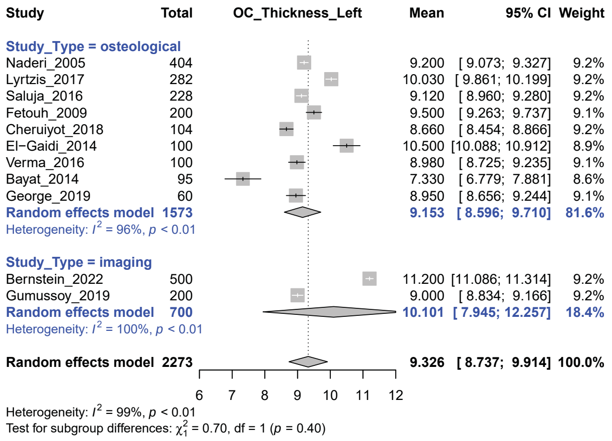

Outlier analysis: Identified outliers

Outlier studies:
"Bayat_2014", "Bernstein_2022", "El-Gaidi_2014"

Forest plot with outliers removed

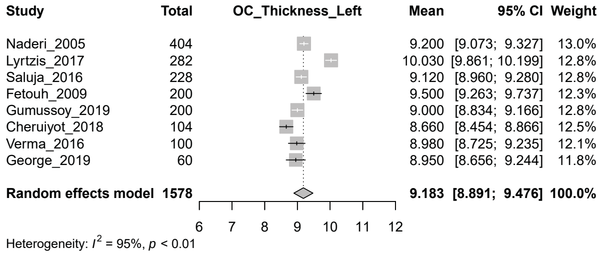

Appendix: Supplemental Figure 11: OC Thickness (Right)

Forest plot evaluating the mean thickness

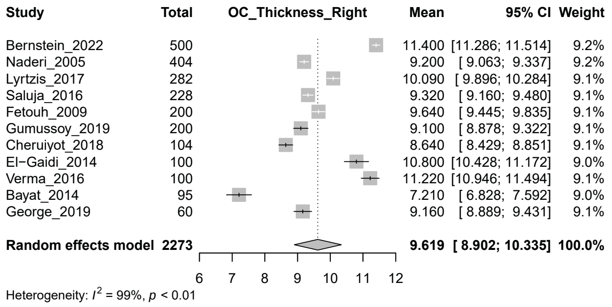

Funnel plot for the assessment of small-study effect

(Test of funnel plot asymmetry: p-value = 0.4233)

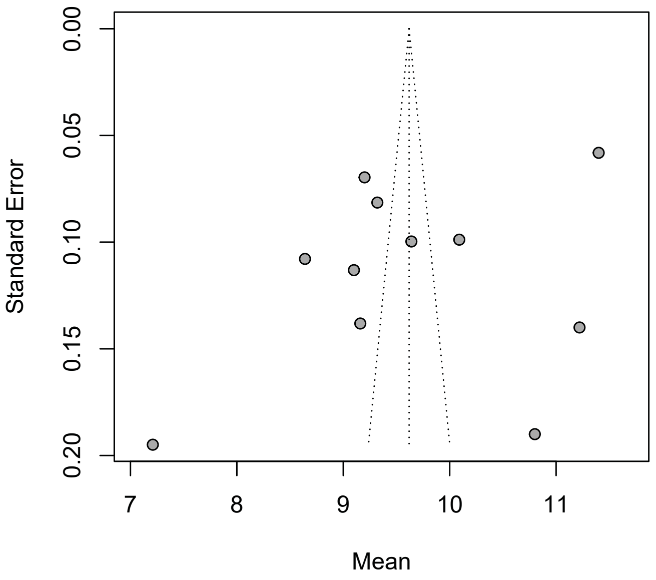

Forest plot of Subgroup analysis based on nationality

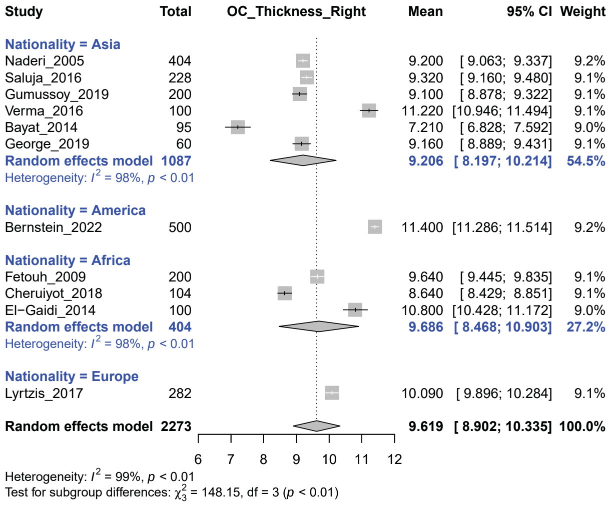

Influence analysis: Influence Diagnostics

(Identified influential studies: none)

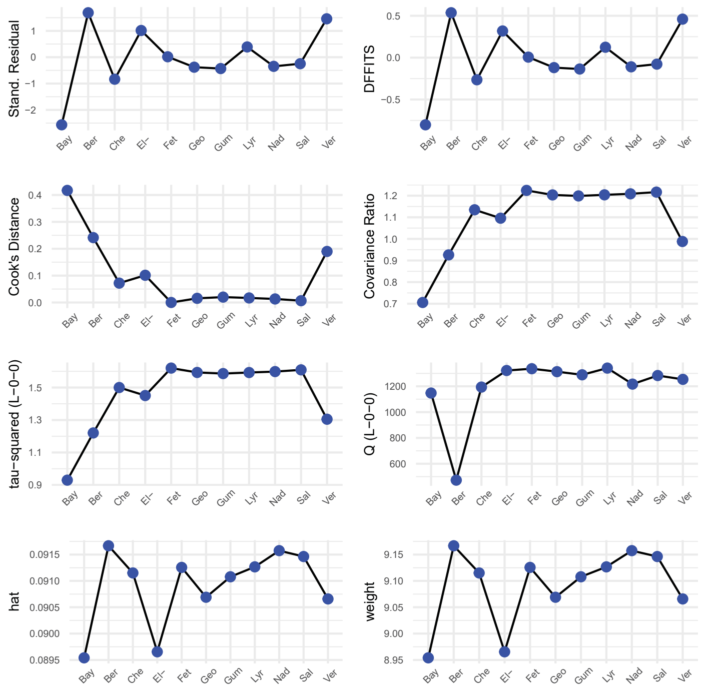

Forest plot of Subgroup analysis based on study's type

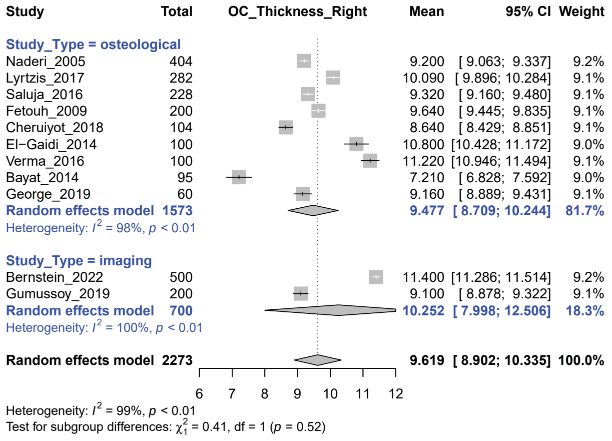

Outlier analysis: Identified outliers

Outlier studies:
"Bayat_2014", "Bernstein_2022", "Cheruiyot_2018", "El-Gaidi_2014", "Verma_2016"

Forest plot with outliers removed

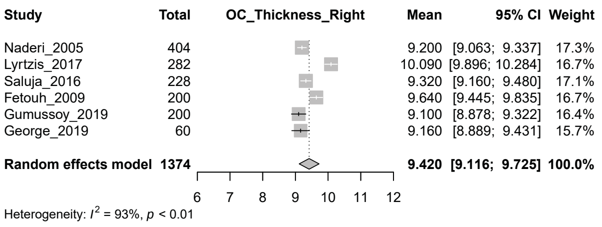

Appendix: Supplemental Figure 12: OC Length (Left vs Right)

Forest plot evaluating the mean length

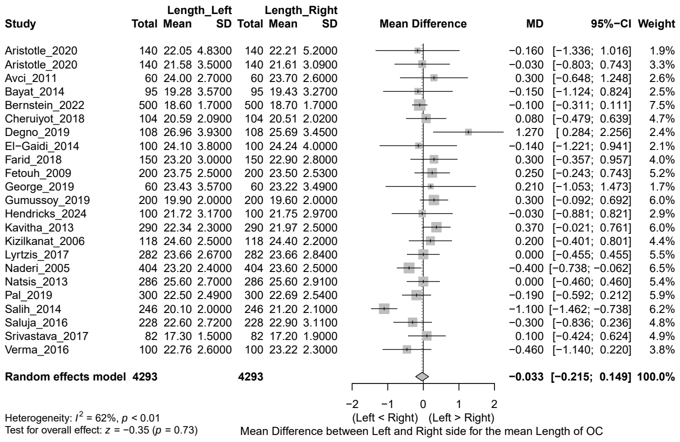

Funnel plot for the assessment of small-study effect

(Test of funnel plot asymmetry: p-value = 0.2154)

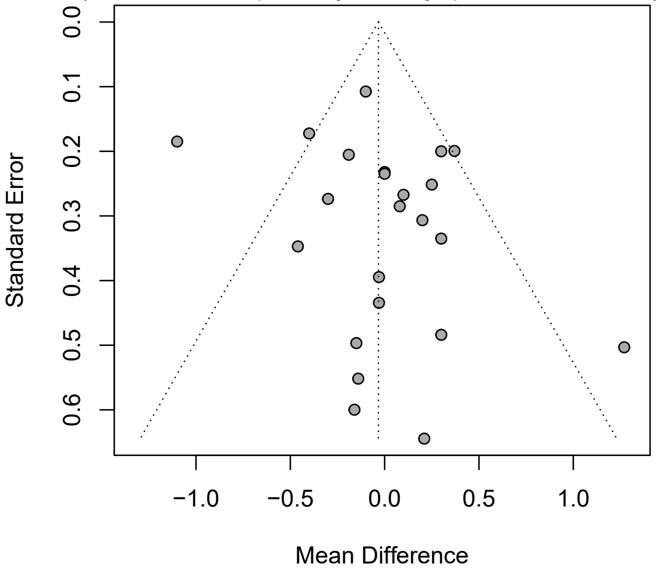

Forest plot of Subgroup analysis based on nationality

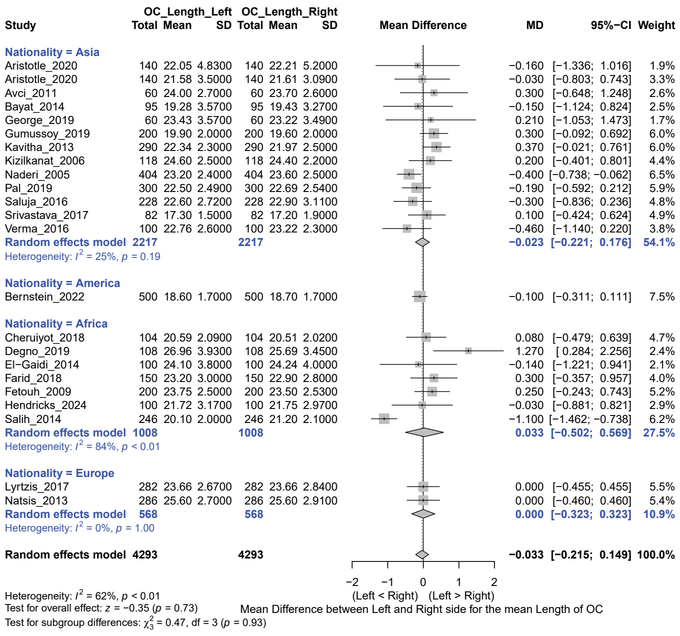

Influence analysis: Influence Diagnostics

(Identified influential studies: "Salih_2014")

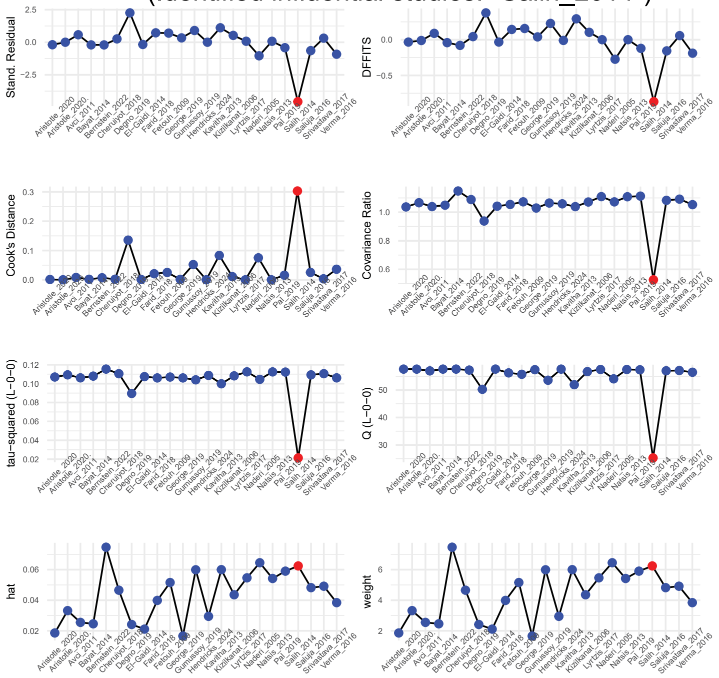

Forest plot of Subgroup analysis based on study's type

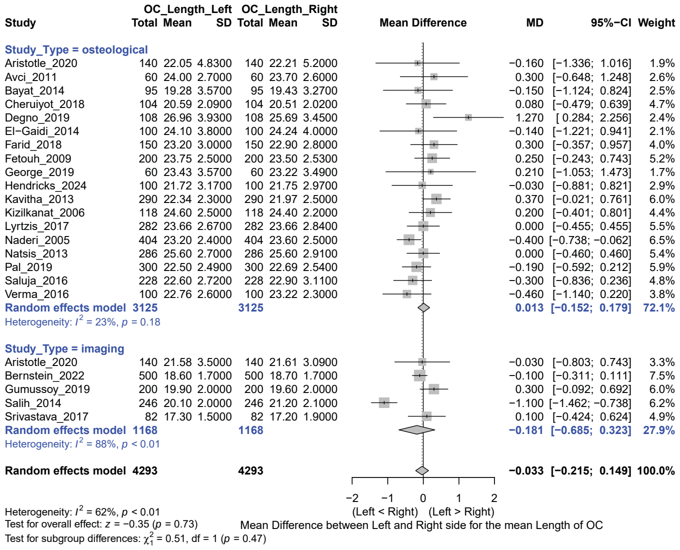

Outlier analysis: Identified outliers

Outlier studies:

"Degno_2019", "Salih_2014"

Forest plot with outliers removed

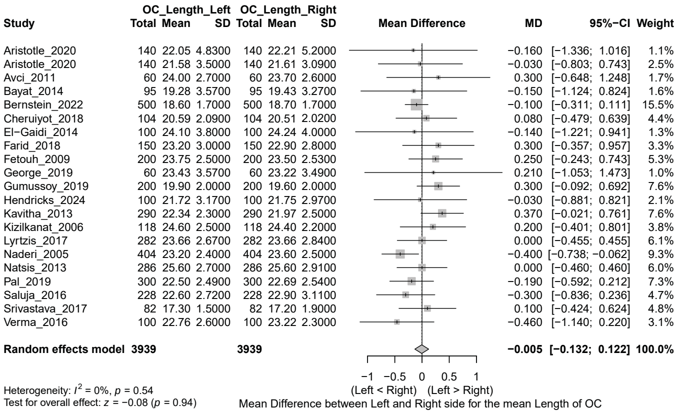

Appendix: Supplemental Figure 13: OC Width (Left vs Right)

Forest plot evaluating the mean width

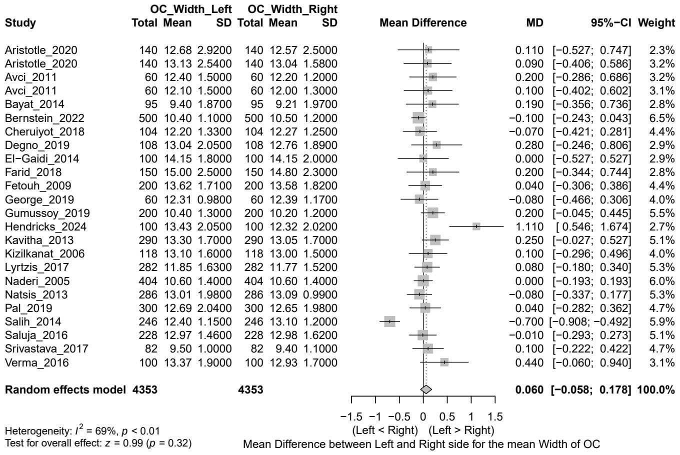

Funnel plot for the assessment of small-study effect

(Test of funnel plot asymmetry: p-value = 0.0118)

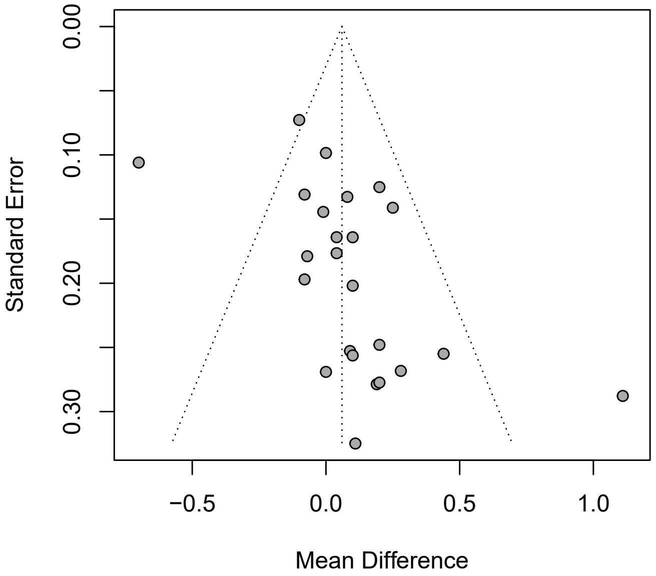

Forest plot of Subgroup analysis based on nationality

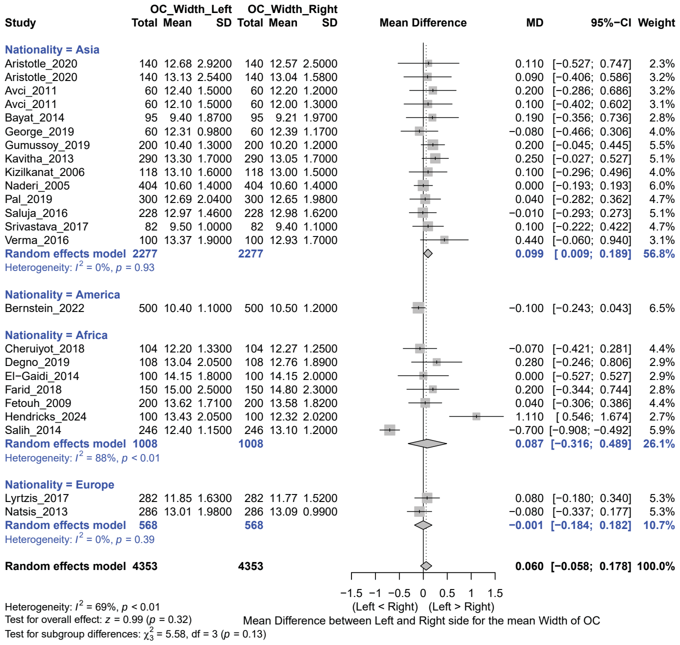

Influence analysis: Influence Diagnostics

(Identified influential studies: none)

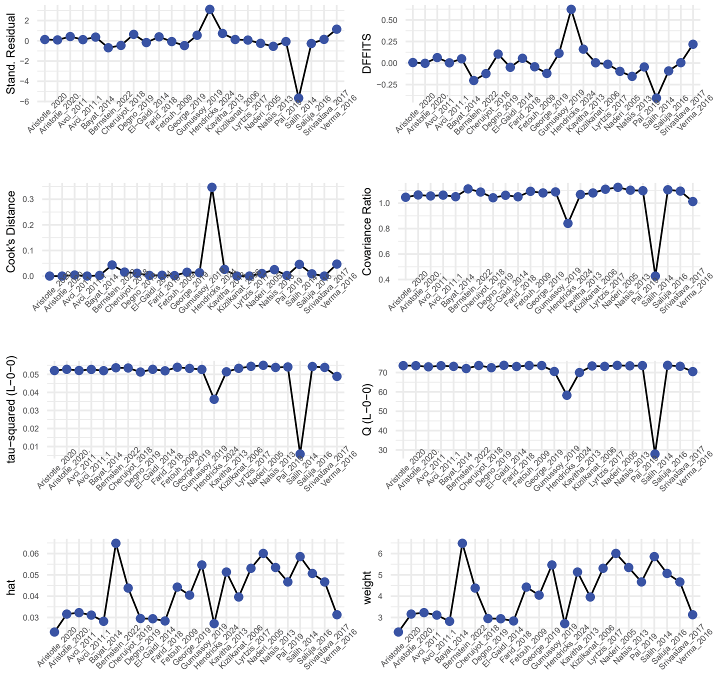

Forest plot of Subgroup analysis based on study's type

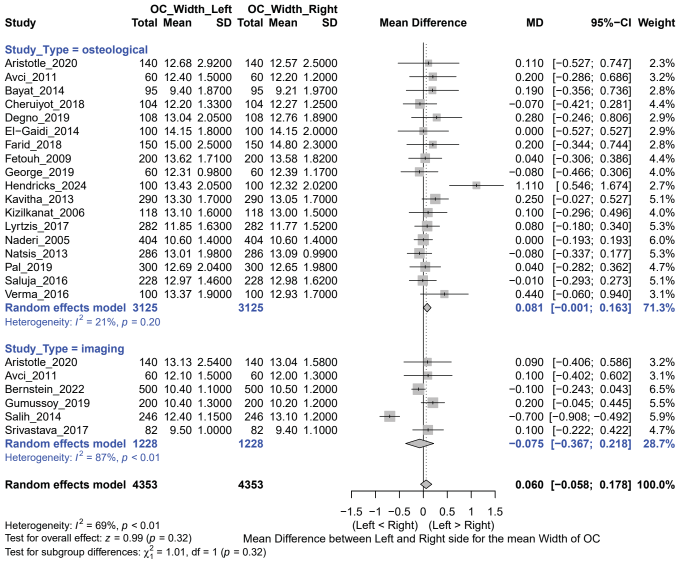

Outlier analysis: Identified outliers

Outlier studies:

"Hendricks_2024", "Salih_2014"

Forest plot with outliers removed

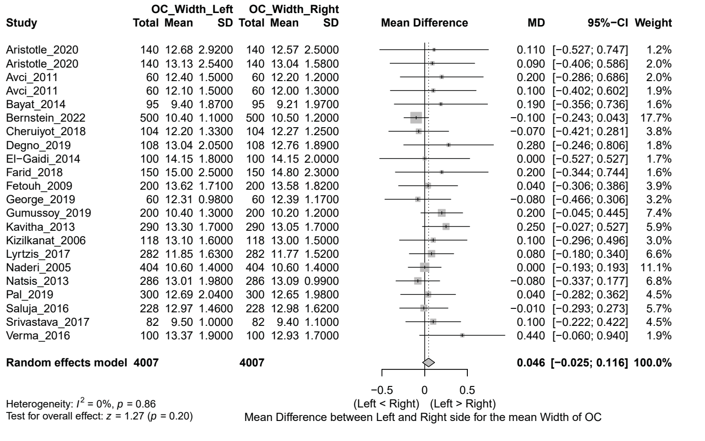

Appendix: Supplemental Figure 14: OC Thickness (Left vs Right)

Forest plot evaluating the mean thickness

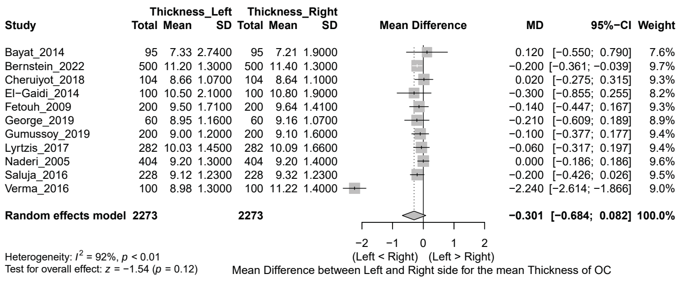

Funnel plot for the assessment of small-study effect

(Test of funnel plot asymmetry: p-value = 0.8154)

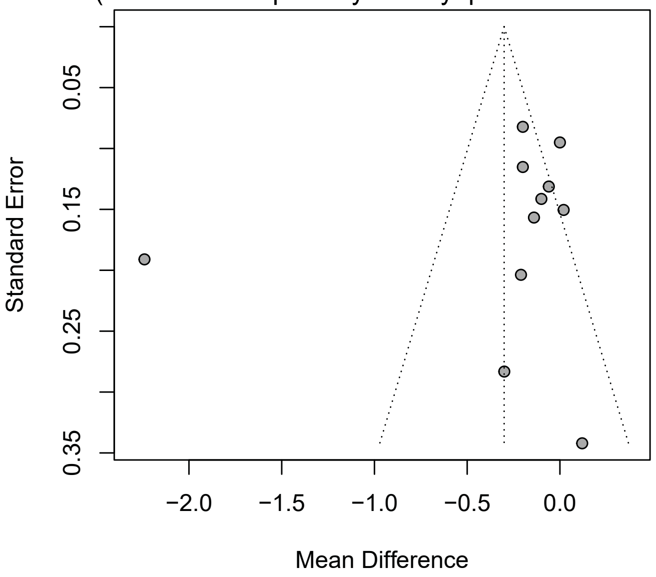

Forest plot of Subgroup analysis based on nationality

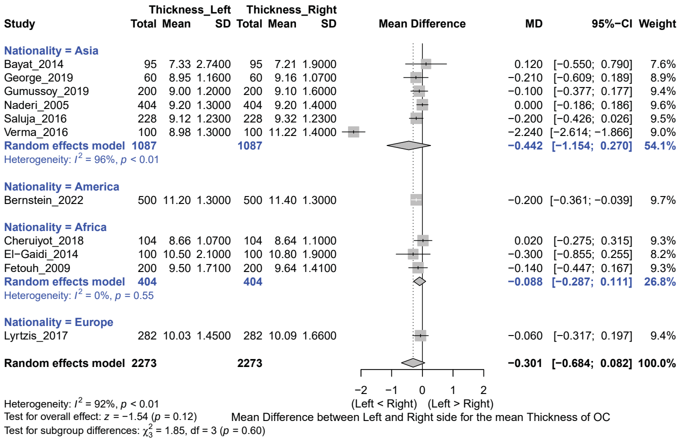

Influence analysis: Influence Diagnostics

(Identified influential studies: "Verma_2016")

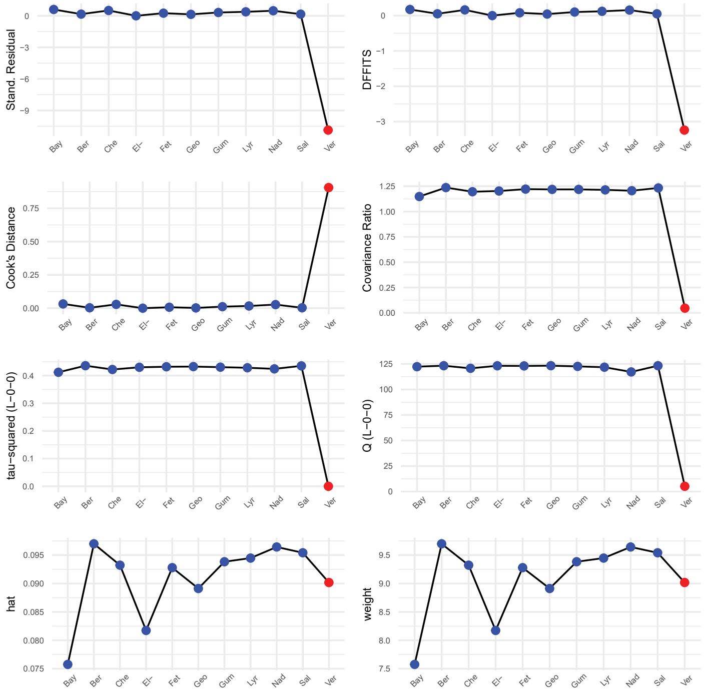

Forest plot of Subgroup analysis based on study's type

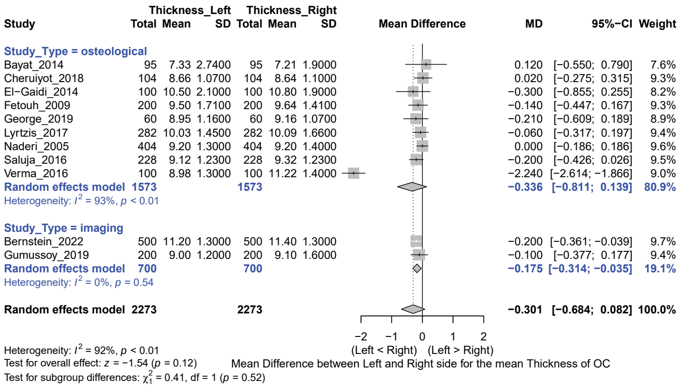

Outlier analysis: Identified outliers

Outlier studies:

"Verma_2016"

Forest plot with outliers removed

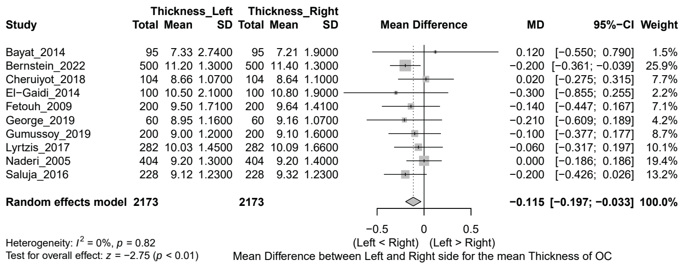

Appendix: Supplemental Figure 15: Males vs Females for OC Length, Width and Thickness; Males vs Females for Left and Right OC Thickness

Forest plot evaluating the mean OC Length: Males vs Females

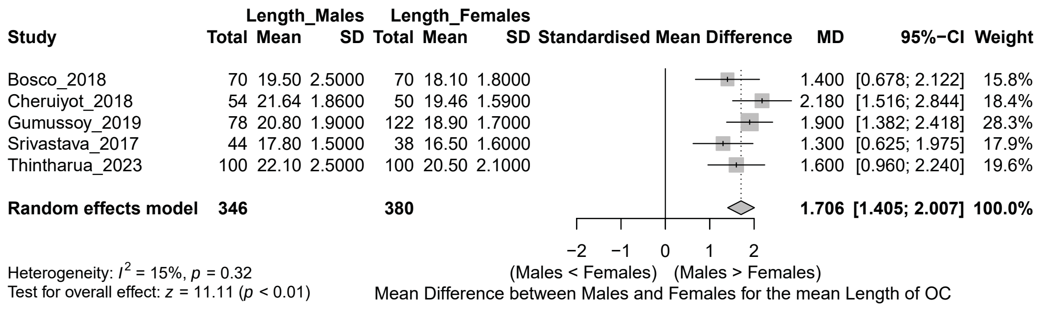

Forest plot evaluating the mean OC Width: Males vs Females

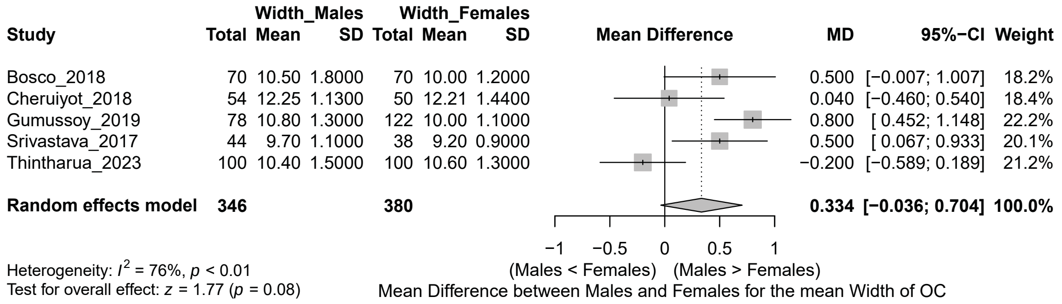

Forest plot evaluating the mean OC Thickness: Males vs Females

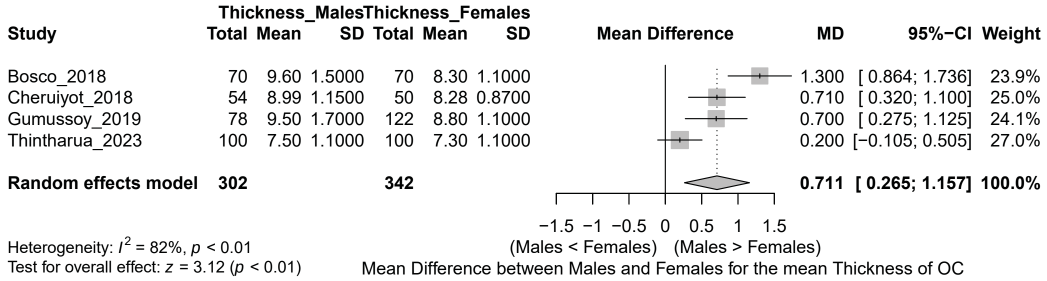

Forest plot evaluating the mean OC Thickness (Left): Males vs Females

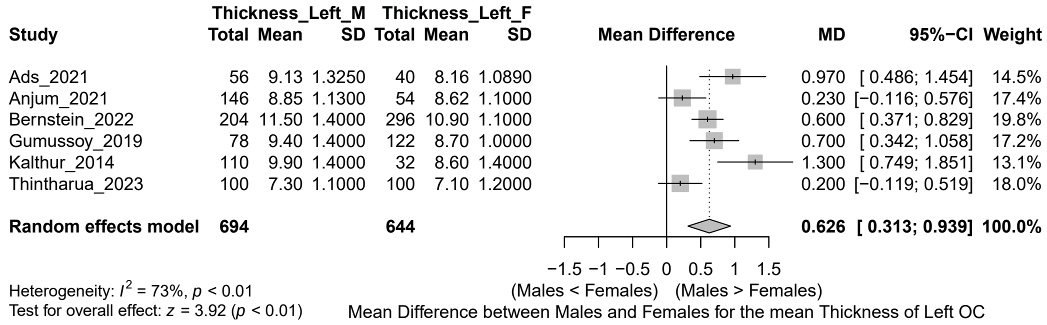

Forest plot evaluating the mean OC Thickness (Right): Males vs Females

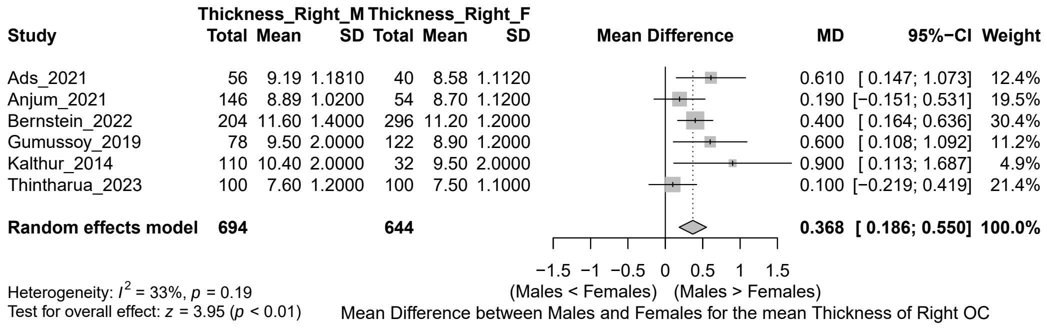

Appendix: Supplemental Figure 16: FM Length (Males vs Females)

Forest plot evaluating the mean length

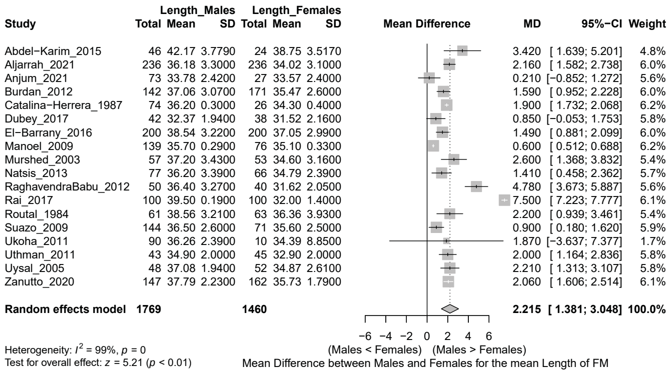

Funnel plot for the assessment of small-study effect

(Test of funnel plot asymmetry: p-value = 0.9388)

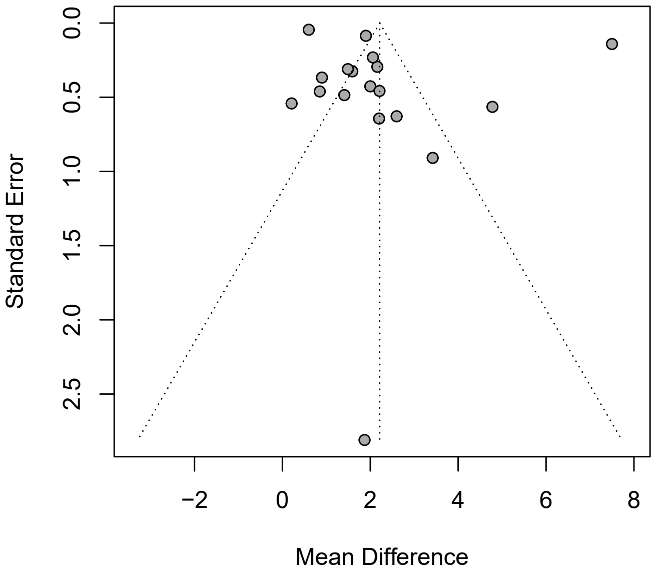

Forest plot of Subgroup analysis based on nationality

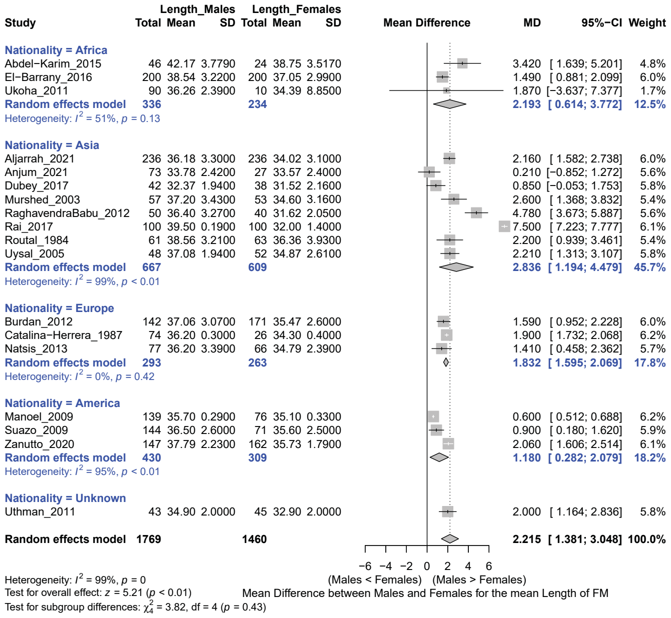

Influence analysis: Influence Diagnostics

(Identified influential studies: "Rai_2017")

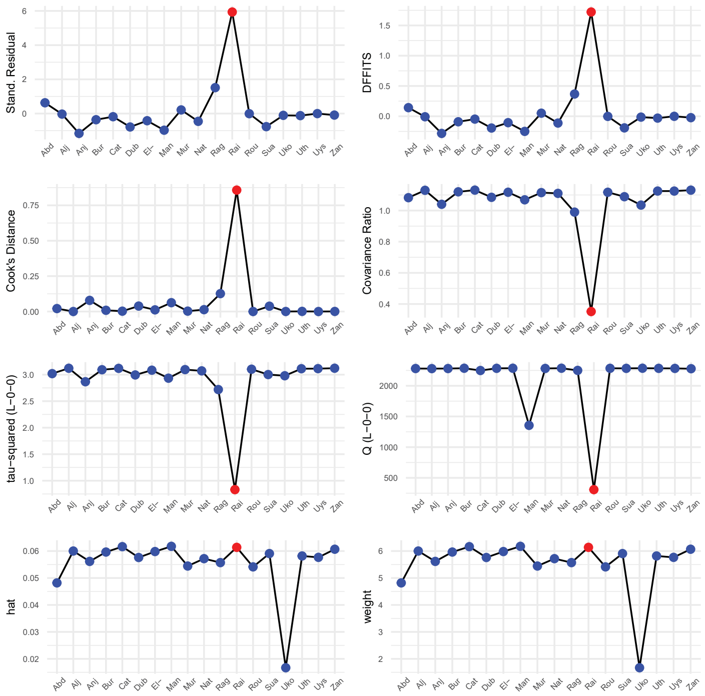

Forest plot of Subgroup analysis based on study's type

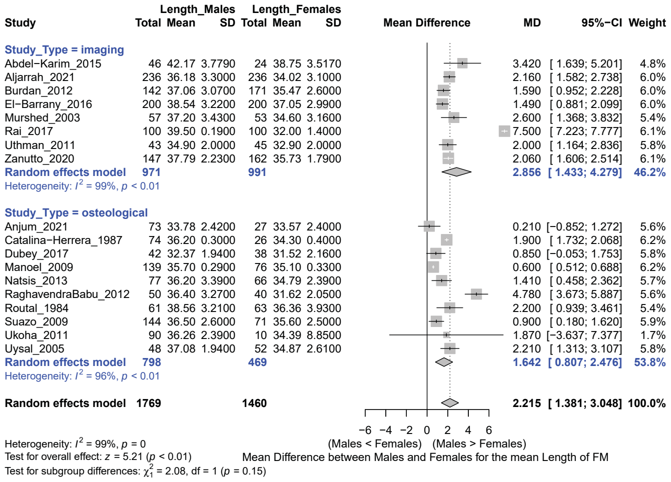

Outlier analysis: Identified outliers

Outlier studies:

"Anjum_2021", "Manoel_2009", "RaghavendraBabu_2012", "Rai_2017"

Forest plot with outliers removed

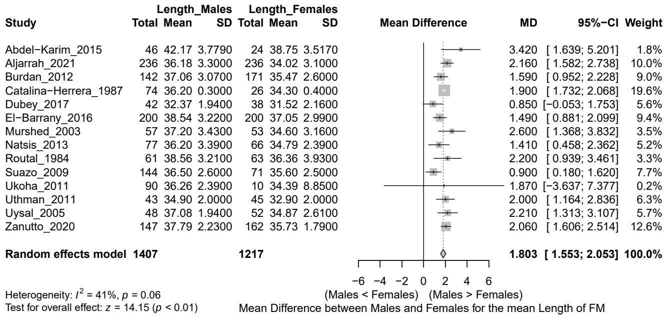

Appendix: Supplemental Figure 17: FM Width (Males vs Females)

Forest plot evaluating the mean width

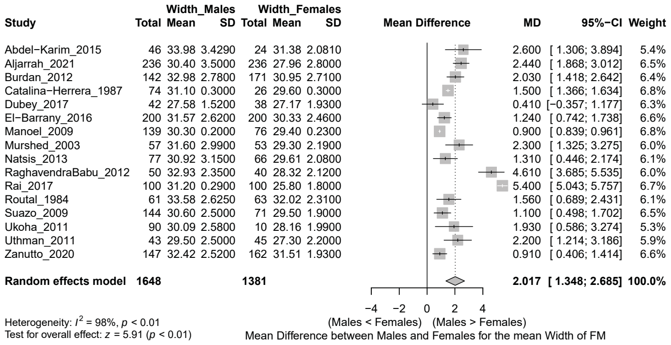

Funnel plot for the assessment of small-study effect

(Test of funnel plot asymmetry: p-value = 0.6425)

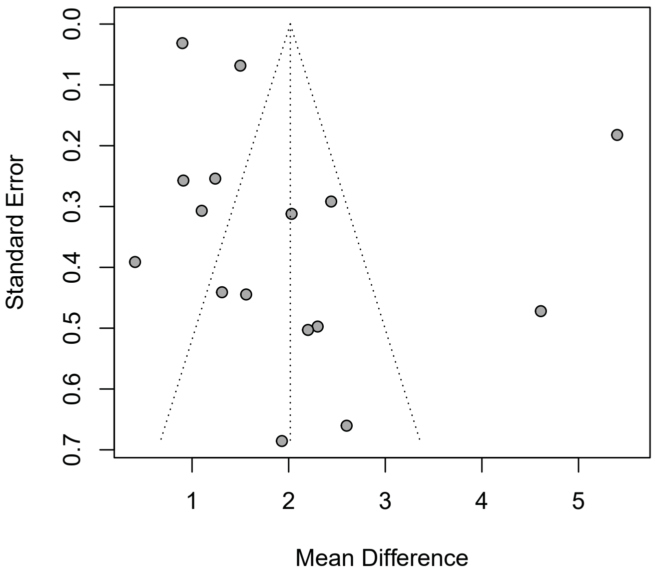

Forest plot of Subgroup analysis based on nationality

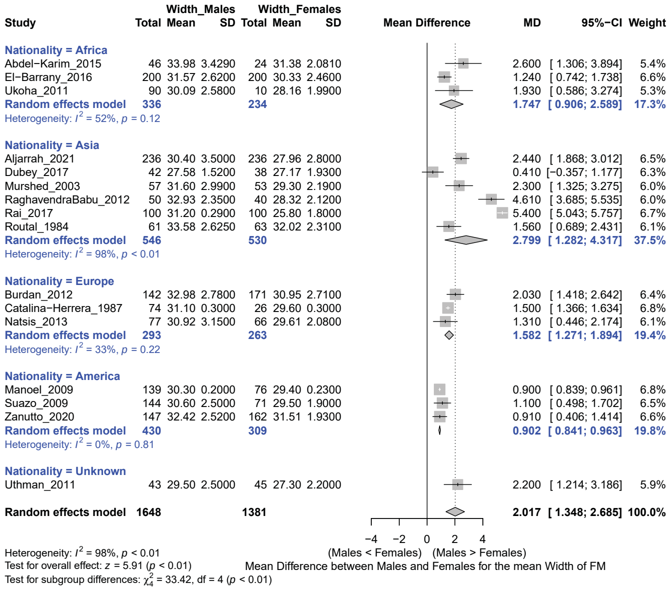

Influence analysis: Influence Diagnostics

(Identified influential studies: "Rai_2017")

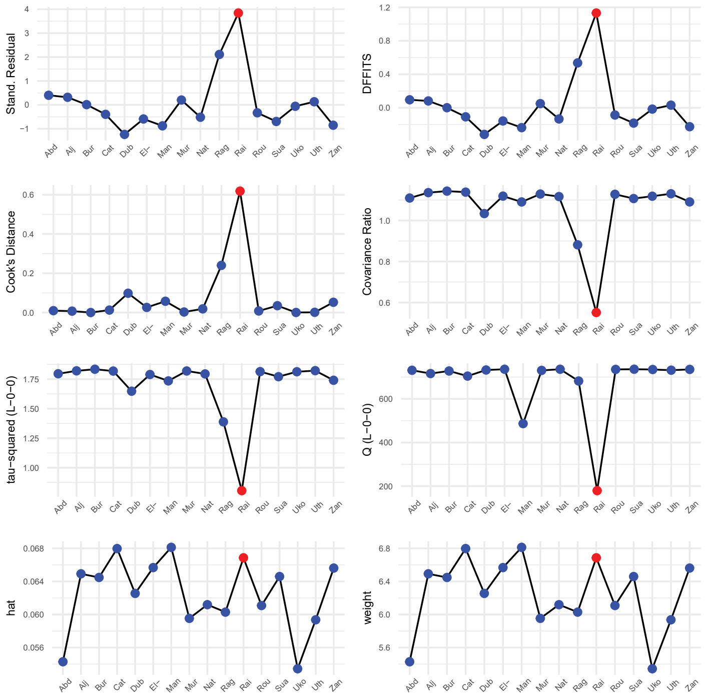

Forest plot of Subgroup analysis based on study's type

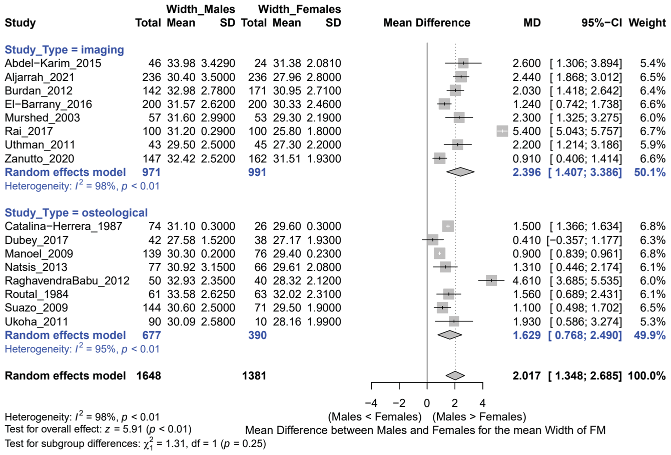

Outlier analysis: Identified outliers

Outlier studies:

"Dubey_2017", "Manoel_2009", "RaghavendraBabu_2012", "Rai_2017"

Forest plot with outliers removed

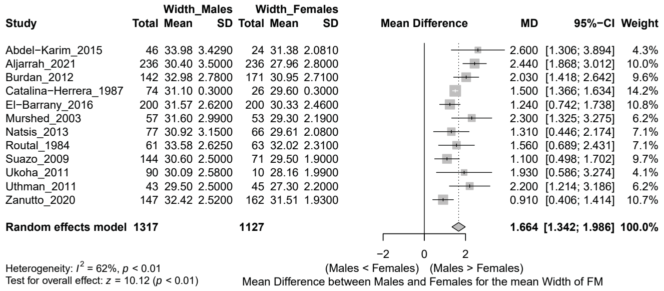

Appendix: Supplemental Figure 18: OC Length (Left): Males vs Females

Forest plot evaluating the mean length

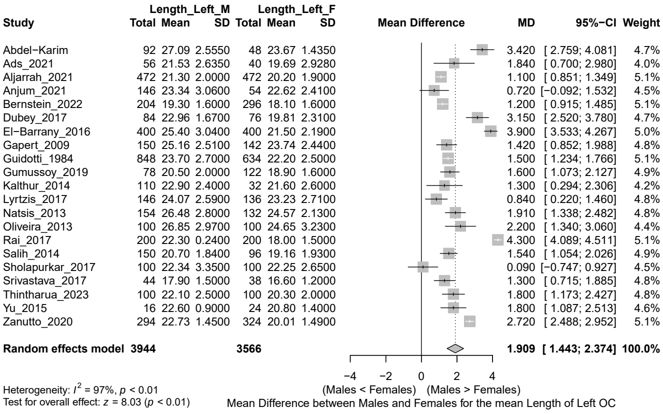

Funnel plot for the assessment of small-study effect

(Test of funnel plot asymmetry: p-value = 0.1582)

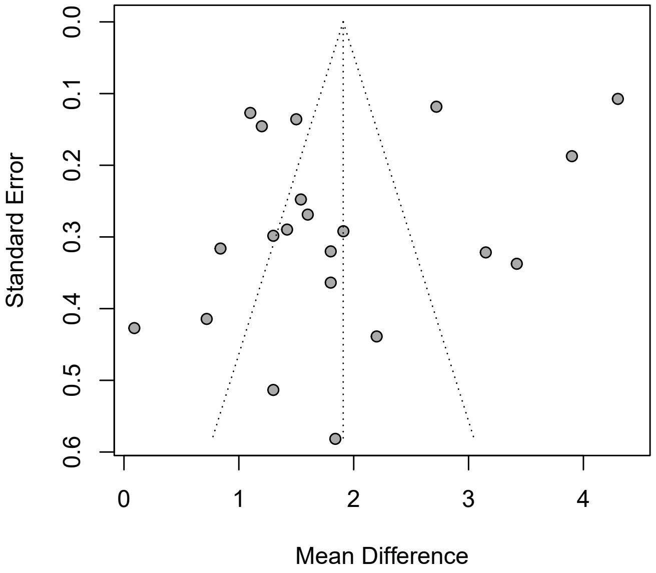

Forest plot of Subgroup analysis based on nationality

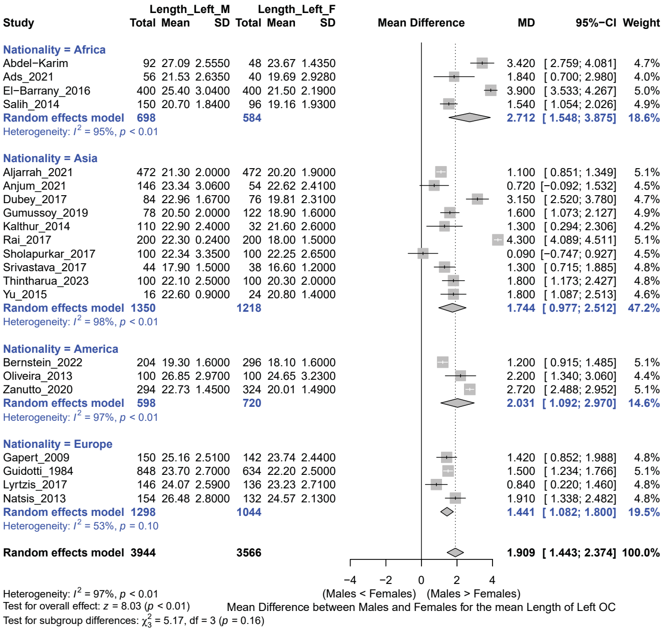

Influence analysis: Influence Diagnostics

(Identified influential studies: none)

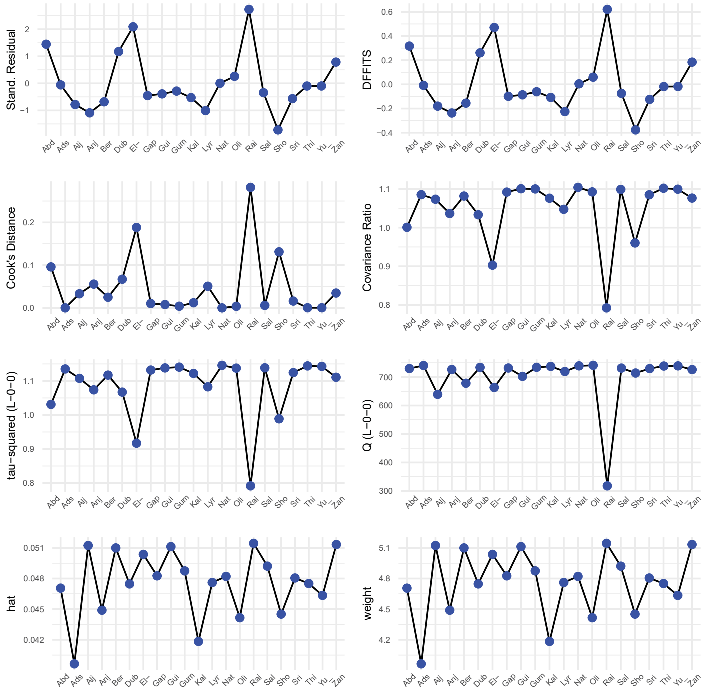

Forest plot of Subgroup analysis based on study's type

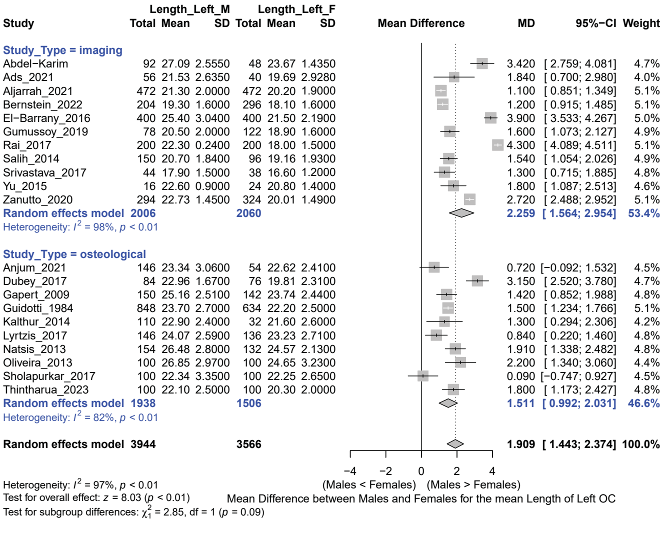

Outlier analysis: Identified outliers

Outlier studies:
"Abdel-Karim", "Aljarrah_2021", "Dubey_2017", "El-Barrany_2016", "Rai_2017", "Sholapurkar_2017", "Zanutto_2020"

Forest plot with outliers removed

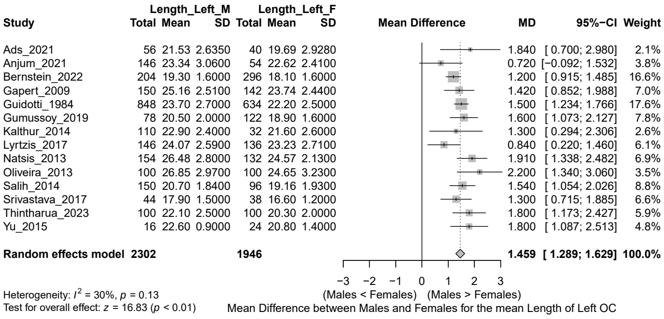

Appendix: Supplemental Figure 19: OC Length (Right): Males vs Females

Forest plot evaluating the mean length

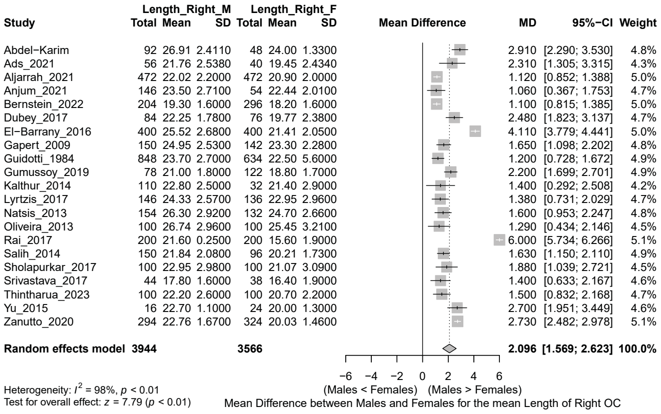

Funnel plot for the assessment of small-study effect

(Test of funnel plot asymmetry: p-value = 0.1357)

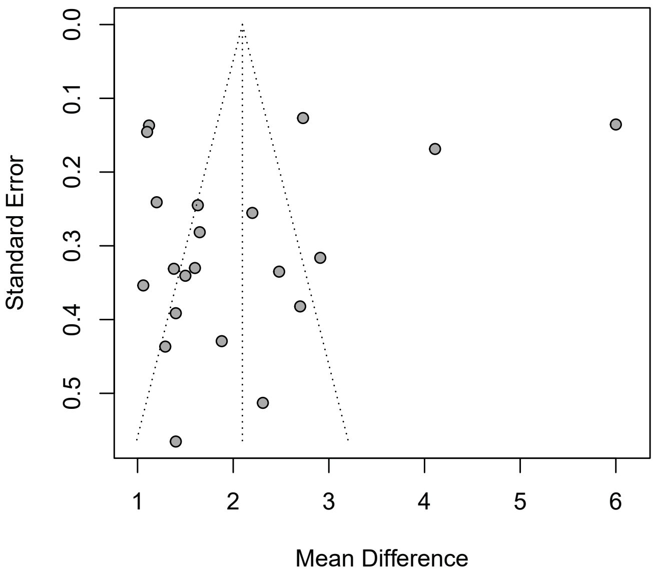

Forest plot of Subgroup analysis based on nationality

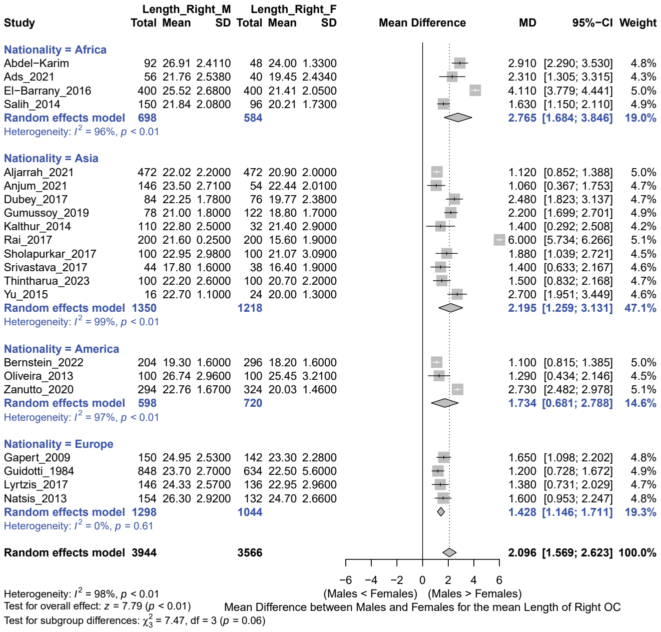

Influence analysis: Influence Diagnostics

(Identified influential studies: "Rai_2017")

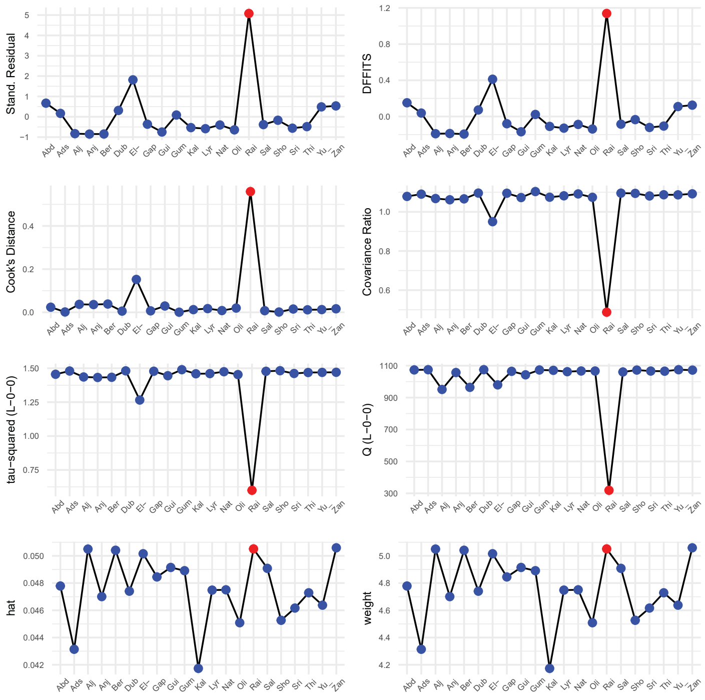

Forest plot of Subgroup analysis based on study's type

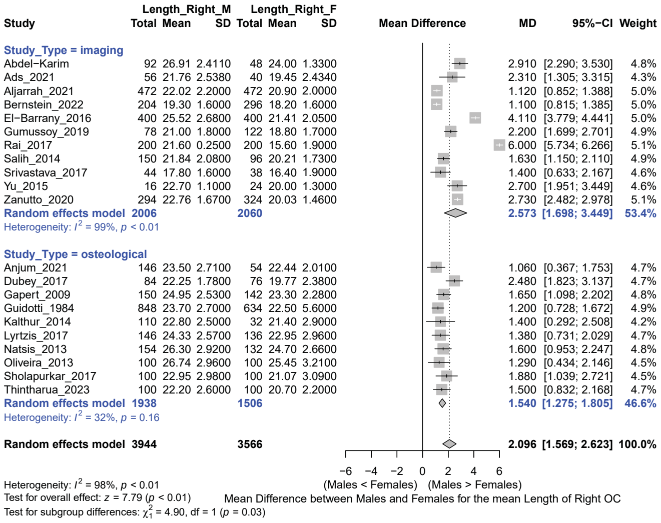

Outlier analysis: Identified outliers

Outlier studies:

"Aljarrah_2021", "Bernstein_2022", "El-Barrany_2016", "Rai_2017"

Forest plot with outliers removed

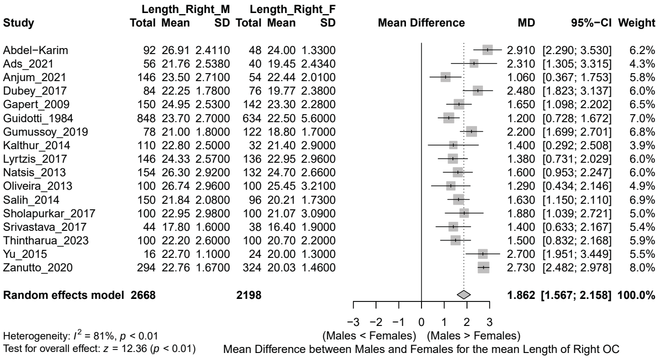

Appendix: Supplemental Figure 20: OC Width (Left): Males vs Females

Forest plot evaluating the mean width

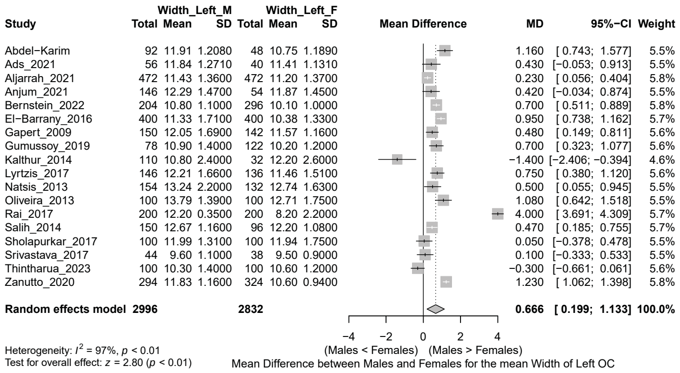

Funnel plot for the assessment of small-study effect

(Test of funnel plot asymmetry: p-value = 0.0566)

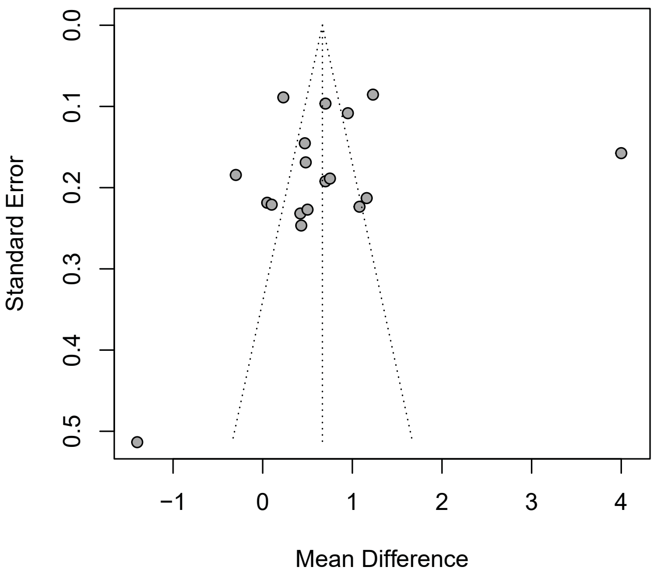

Forest plot of Subgroup analysis based on nationality

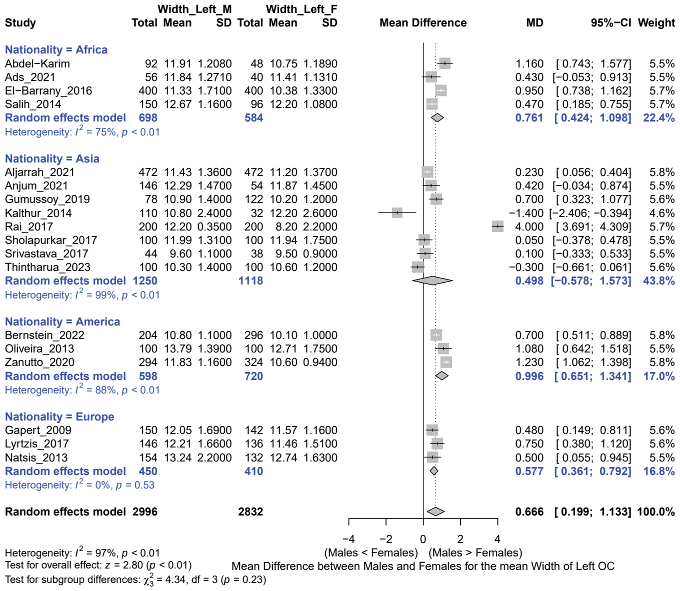

Influence analysis: Influence Diagnostics

(Identified influential studies: "Rai_2017")

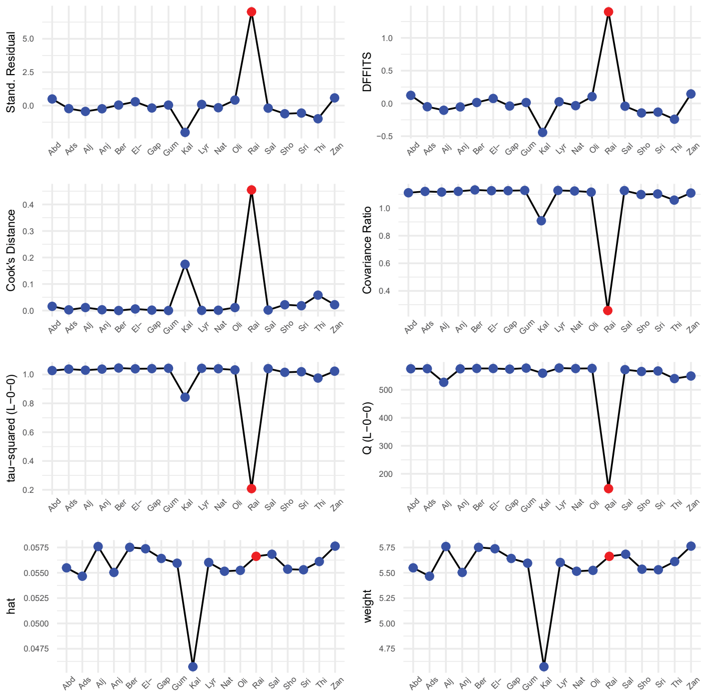

Forest plot of Subgroup analysis based on study's type

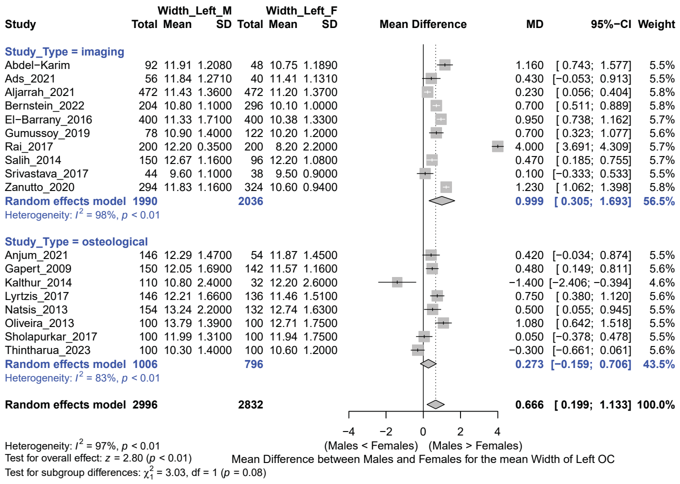

Outlier analysis: Identified outliers

Outlier studies:

"Kalthur_2014", "Rai_2017", "Thintharua_2023"

Forest plot with outliers removed

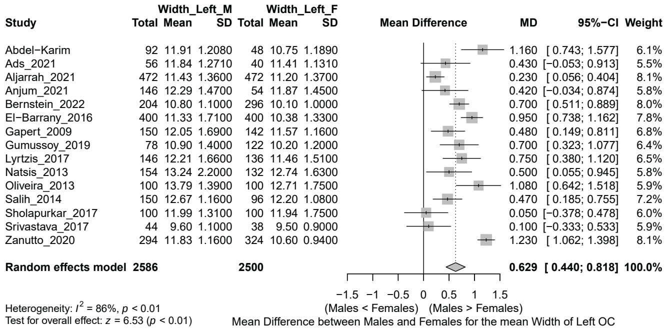

Appendix: Supplemental Figure 21: OC Width (Right): Males vs Females

Forest plot evaluating the mean width

Study	Width_Right_M Total	Mean	SD	Width_Right_F Total	Mean	SD	MD	95%−CI	Weight
Abdel−Karim	92	12.22	1.3320	48	11.13	1.1160	1.090	[0.673; 1.507]	5.9%
Aljarrah_2021	472	11.30	1.4000	472	10.99	1.4100	0.310	[0.131; 0.489]	6.1%
Anjum_2021	146	12.19	1.5300	54	11.46	1.5600	0.730	[0.246; 1.214]	5.8%
Bernstein_2022	204	11.00	1.1000	296	10.20	1.2000	0.800	[0.596; 1.004]	6.0%
El−Barrany_2016	400	11.39	1.5100	400	10.62	1.3400	0.770	[0.572; 0.968]	6.1%
Gapert_2009	150	12.01	1.4100	142	11.42	1.2100	0.590	[0.289; 0.891]	6.0%
Gumussoy_2019	78	10.70	1.2000	122	9.90	1.1000	0.800	[0.470; 1.130]	5.9%
Kalthur_2014	110	10.50	1.8000	32	12.00	2.3000	−1.500	[−2.365; −0.635]	5.1%
Lyrtzis_2017	146	12.10	1.5000	136	11.43	1.4700	0.670	[0.323; 1.017]	5.9%
Natsis_2013	154	13.13	2.0100	132	13.04	1.9900	0.090	[−0.375; 0.555]	5.8%
Oliveira_2013	100	13.51	1.3800	100	12.68	1.5600	0.830	[0.422; 1.238]	5.9%
Rai_2017	200	10.50	0.2200	200	6.60	1.1000	3.900	[3.745; 4.055]	6.1%
Salih_2014	150	13.26	1.1800	96	12.90	1.2100	0.360	[0.053; 0.667]	6.0%
Sholapurkar_2017	100	11.36	1.7700	100	11.43	1.6700	−0.070	[−0.547; 0.407]	5.8%
Srivastava_2017	44	9.80	1.1000	38	9.00	0.9000	0.800	[0.367; 1.233]	5.8%
Thintharua_2023	100	10.50	1.6000	100	10.70	1.5000	−0.200	[−0.630; 0.230]	5.8%
Zanutto_2020	294	11.73	1.1200	324	10.55	0.9400	1.180	[1.016; 1.344]	6.1%
**Random effects model**	**2940**			**2792**			**0.680**	**[0.189; 1.171]**	**100.0%**

−4 −2 0 2 4
(Males < Females) (Males > Females)
Heterogeneity: I^2 = 99%, p < 0.01
Test for overall effect: z = 2.71 (p < 0.01)
Mean Difference between Males and Females for the mean Width of Right OC

Funnel plot for the assessment of small-study effect

(Test of funnel plot asymmetry: p-value = 0.0050)

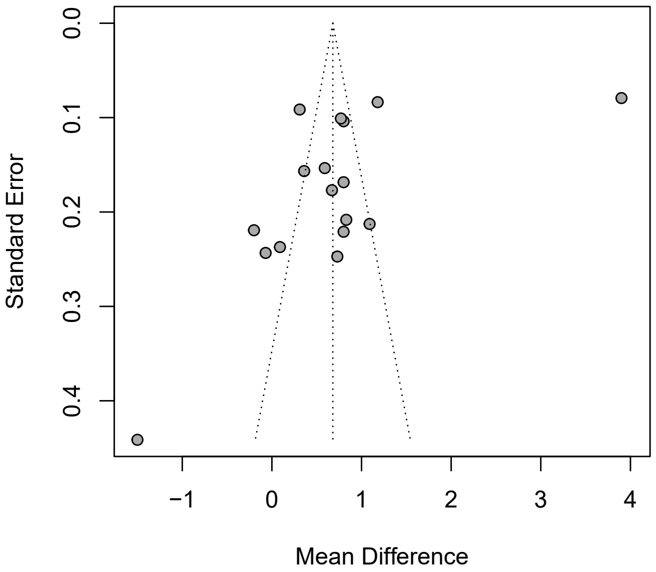

Forest plot of Subgroup analysis based on nationality

Study	Width_Right_M Total	Mean	SD	Width_Right_F Total	Mean	SD	MD	95%−CI	Weight
**Nationality = Africa**									
Abdel−Karim	92	12.22	1.3320	48	11.13	1.1160	1.090	[0.673; 1.507]	5.9%
El−Barrany_2016	400	11.39	1.5100	400	10.62	1.3400	0.770	[0.572; 0.968]	6.1%
Salih_2014	150	13.26	1.1800	96	12.90	1.2100	0.360	[0.053; 0.667]	6.0%
**Random effects model**	**642**			**544**			**0.724**	**[0.336; 1.112]**	**17.9%**
Heterogeneity: I^2 = 77%, p = 0.01									
**Nationality = Asia**									
Aljarrah_2021	472	11.30	1.4000	472	10.99	1.4100	0.310	[0.131; 0.489]	6.1%
Anjum_2021	146	12.19	1.5300	54	11.46	1.5600	0.730	[0.246; 1.214]	5.8%
Gumussoy_2019	78	10.70	1.2000	122	9.90	1.1000	0.800	[0.470; 1.130]	5.9%
Kalthur_2014	110	10.50	1.8000	32	12.00	2.3000	−1.500	[−2.365; −0.635]	5.1%
Rai_2017	200	10.50	0.2200	200	6.60	1.1000	3.900	[3.745; 4.055]	6.1%
Sholapurkar_2017	100	11.36	1.7700	100	11.43	1.6700	−0.070	[−0.547; 0.407]	5.8%
Srivastava_2017	44	9.80	1.1000	38	9.00	0.9000	0.800	[0.367; 1.233]	5.8%
Thintharua_2023	100	10.50	1.6000	100	10.70	1.5000	−0.200	[−0.630; 0.230]	5.8%
**Random effects model**	**1250**			**1118**			**0.619**	**[−0.447; 1.685]**	**46.4%**
Heterogeneity: I^2 = 99%, p < 0.01									
**Nationality = America**									
Bernstein_2022	204	11.00	1.1000	296	10.20	1.2000	0.800	[0.596; 1.004]	6.0%
Oliveira_2013	100	13.51	1.3800	100	12.68	1.5600	0.830	[0.422; 1.238]	5.9%
Zanutto_2020	294	11.73	1.1200	324	10.55	0.9400	1.180	[1.016; 1.344]	6.1%
**Random effects model**	**598**			**720**			**0.960**	**[0.690; 1.229]**	**18.0%**
Heterogeneity: I^2 = 78%, p = 0.01									
**Nationality = Europe**									
Gapert_2009	150	12.01	1.4100	142	11.42	1.2100	0.590	[0.289; 0.891]	6.0%
Lyrtzis_2017	146	12.10	1.5000	136	11.43	1.4700	0.670	[0.323; 1.017]	5.9%
Natsis_2013	154	13.13	2.0100	132	13.04	1.9900	0.090	[−0.375; 0.555]	5.8%
**Random effects model**	**450**			**410**			**0.489**	**[0.181; 0.796]**	**17.7%**
Heterogeneity: I^2 = 53%, p = 0.12									
**Random effects model**	**2940**			**2792**			**0.680**	**[0.189; 1.171]**	**100.0%**

−4 −2 0 2 4
(Males < Females) (Males > Females)
Heterogeneity: I^2 = 99%, p < 0.01
Test for overall effect: z = 2.71 (p < 0.01)
Test for subgroup differences: χ^2_3 = 5.17, df = 3 (p = 0.16)
Mean Difference between Males and Females for the mean Width of Right OC

Influence analysis: Influence Diagnostics

(Identified influential studies: "Rai_2017")

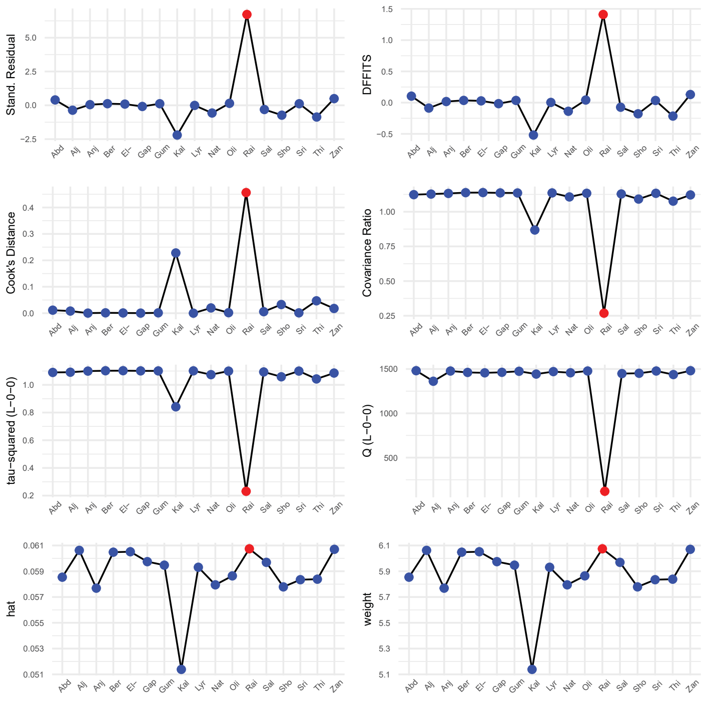

Forest plot of Subgroup analysis based on study's type

Study	Width_Right_M Total	Mean	SD	Width_Right_F Total	Mean	SD	MD	95%−CI	Weight
**Study_Type = imaging**									
Abdel−Karim	92	12.22	1.3320	48	11.13	1.1160	1.090	[0.673; 1.507]	5.9%
Aljarrah_2021	472	11.30	1.4000	472	10.99	1.4100	0.310	[0.131; 0.489]	6.1%
Bernstein_2022	204	11.00	1.1000	296	10.20	1.2000	0.800	[0.596; 1.004]	6.0%
El−Barrany_2016	400	11.39	1.5100	400	10.62	1.3400	0.770	[0.572; 0.968]	6.1%
Gumussoy_2019	78	10.70	1.2000	122	9.90	1.1000	0.800	[0.470; 1.130]	5.9%
Rai_2017	200	10.50	0.2200	200	6.60	1.1000	3.900	[3.745; 4.055]	6.1%
Salih_2014	150	13.26	1.1800	96	12.90	1.2100	0.360	[0.053; 0.667]	6.0%
Srivastava_2017	44	9.80	1.1000	38	9.00	0.9000	0.800	[0.367; 1.233]	5.8%
Zanutto_2020	294	11.73	1.1200	324	10.55	0.9400	1.180	[1.016; 1.344]	6.1%
**Random effects model**	**1934**			**1996**			**1.116**	**[0.400; 1.831]**	**53.9%**
Heterogeneity: I^2 = 99%, p < 0.01									
**Study_Type = osteological**									
Anjum_2021	146	12.19	1.5300	54	11.46	1.5600	0.730	[0.246; 1.214]	5.8%
Gapert_2009	150	12.01	1.4100	142	11.42	1.2100	0.590	[0.289; 0.891]	6.0%
Kalthur_2014	110	10.50	1.8000	32	12.00	2.3000	−1.500	[−2.365; −0.635]	5.1%
Lyrtzis_2017	146	12.10	1.5000	136	11.43	1.4700	0.670	[0.323; 1.017]	5.9%
Natsis_2013	154	13.13	2.0100	132	13.04	1.9900	0.090	[−0.375; 0.555]	5.8%
Oliveira_2013	100	13.51	1.3800	100	12.68	1.5600	0.830	[0.422; 1.238]	5.9%
Sholapurkar_2017	100	11.36	1.7700	100	11.43	1.6700	−0.070	[−0.547; 0.407]	5.8%
Thintharua_2023	100	10.50	1.6000	100	10.70	1.5000	−0.200	[−0.630; 0.230]	5.8%
**Random effects model**	**1006**			**796**			**0.202**	**[−0.259; 0.663]**	**46.1%**
Heterogeneity: I^2 = 83%, p < 0.01									
**Random effects model**	**2940**			**2792**			**0.680**	**[0.189; 1.171]**	**100.0%**

−4 −2 0 2 4
(Males < Females) (Males > Females)
Heterogeneity: I^2 = 99%, p < 0.01
Test for overall effect: z = 2.71 (p < 0.01)
Test for subgroup differences: χ^2_1 = 4.43, df = 1 (p = 0.04)
Mean Difference between Males and Females for the mean Width of Right OC

Outlier analysis: Identified outliers

Outlier studies:
"Kalthur_2014", "Rai_2017"

Forest plot with outliers removed

Study	Width_Right_M Total	Mean	SD	Width_Right_F Total	Mean	SD	MD	95%−CI	Weight
Abdel−Karim	92	12.22	1.3320	48	11.13	1.1160	1.090	[0.673; 1.507]	6.1%
Aljarrah_2021	472	11.30	1.4000	472	10.99	1.4100	0.310	[0.131; 0.489]	7.9%
Anjum_2021	146	12.19	1.5300	54	11.46	1.5600	0.730	[0.246; 1.214]	5.6%
Bernstein_2022	204	11.00	1.1000	296	10.20	1.2000	0.800	[0.596; 1.004]	7.7%
El−Barrany_2016	400	11.39	1.5100	400	10.62	1.3400	0.770	[0.572; 0.968]	7.8%
Gapert_2009	150	12.01	1.4100	142	11.42	1.2100	0.590	[0.289; 0.891]	7.0%
Gumussoy_2019	78	10.70	1.2000	122	9.90	1.1000	0.800	[0.470; 1.130]	6.8%
Lyrtzis_2017	146	12.10	1.5000	136	11.43	1.4700	0.670	[0.323; 1.017]	6.7%
Natsis_2013	154	13.13	2.0100	132	13.04	1.9900	0.090	[−0.375; 0.555]	5.7%
Oliveira_2013	100	13.51	1.3800	100	12.68	1.5600	0.830	[0.422; 1.238]	6.2%
Salih_2014	150	13.26	1.1800	96	12.90	1.2100	0.360	[0.053; 0.667]	7.0%
Sholapurkar_2017	100	11.36	1.7700	100	11.43	1.6700	−0.070	[−0.547; 0.407]	5.6%
Srivastava_2017	44	9.80	1.1000	38	9.00	0.9000	0.800	[0.367; 1.233]	6.0%
Thintharua_2023	100	10.50	1.6000	100	10.70	1.5000	−0.200	[−0.630; 0.230]	6.0%
Zanutto_2020	294	11.73	1.1200	324	10.55	0.9400	1.180	[1.016; 1.344]	7.9%
**Random effects model**	**2630**			**2560**			**0.601**	**[0.404; 0.798]**	**100.0%**

−1.5 −1 −0.5 0 0.5 1 1.5
(Males < Females) (Males > Females)
Heterogeneity: I^2 = 85%, p < 0.01
Test for overall effect: z = 5.98 (p < 0.01)
Mean Difference between Males and Females for the mean Width of Right OC
